# A photoexcited halogen-bonded EDA complex of the thiophenolate anion with iodobenzene for C(sp^3^)–H activation and thiolation†

**DOI:** 10.1039/d1sc03667j

**Published:** 2021-10-21

**Authors:** Tao Li, Kangjiang Liang, Jiaying Tang, Yuzhen Ding, Xiaogang Tong, Chengfeng Xia

**Affiliations:** Key Laboratory of Medicinal Chemistry for Natural Resource, Ministry of Education, Yunnan Provincial Center for Research & Development of Natural Products, School of Chemical Science and Technology, Yunnan University Kunming 650091 China xiacf@ynu.edu.cn

## Abstract

Thiophenol was discovered to form an EDA complex with iodobenzene through halogen bonding interactions upon treatment with KOH. A direct photochemical thiolation of C(sp^3^)–H bond-containing etheric, allylic, and benzylic substrates with thiophenol was developed. The reaction proceeded on the basis of the *in situ* generation of a thiyl radical and aryl radical through single electron transfer between the photoexcited thiophenolate anion and aryl iodide EDA complex. Then a C(sp^3^) centred-radical was formed by aryl radical-mediated hydrogen atom transfer and the thiolation products were delivered *via* a radical–radical cross-coupling with the thiyl radical.

## Abstract 2

A direct photochemical thiolation of C(sp^3^)–H bond-containing substrates with thiophenol was developed. A halogen bonding-type EDA complex was found to trigger the downstream single electron transfer and hydrogen atom transfer process.

## Graphical abstract

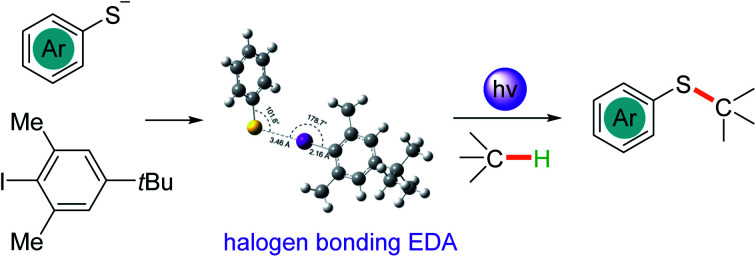

## 1. Introduction

A visible light-induced photoreaction generates electronically excited open-shell species which expand novel and unique organic chemistry. Since most organic molecules do not possess the ability to absorb visible light, an external photocatalyst (*e.g.*, ruthenium and iridium salts with pyridine-derived ligands, or organic dyes) is often required for desired chemical transformation.^1^ As an alternative, an electron donor–acceptor (EDA) complex, which is produced by ground-state association between an electron-rich donor and an electron-deficient acceptor through electrostatic interactions, can sometimes absorb light in the visible region and an electron-transfer event can occur without the need for any photocatalysts.^2^ The EDA complex can be formed by several recognition interactions, such as σ-type interactions, anion–π interactions, hydrogen bonding, and halogen bonding.^3^ Halogen bonding is a type of noncovalent interaction between a halogen atom (halogen bonding donor) and a negative site (halogen bonding acceptor) in two different molecules.^4^ The noncovalent interactions could be effectively analyzed and predicted from the electrostatic potential.^5^ It is discovered that there is a region of positive electrostatic potential surrounded by negative electrostatic potential, termed a “σ-hole”, on the outermost portions of the halogen atom surface.^6^ This electron deficiency area is responsible for the formation of halogen bonding interactions with a nucleophilic entity. Meanwhile, the halogen bonding interactions feature a near-linear structure with angles primarily between 160° and 180° because the “σ-hole” is centered on the *C*–*X* axis.^7^ Therefore, greater directionality in halogen bonding interactions is the advantage compared with other intermolecular non-covalent interactions, such as hydrogen bonding. In particular, the iodine atom participates in noncovalent interactions with electron-rich atoms or groups to form more efficient halogen bonding in the order I > Br > Cl.^6*b*^ The halogen bonding EDA complexes have been exploited as efficient protocol for the photochemical transformation.^8^

Organosulfur compounds have received great attention in the fields of pharmaceutical chemistry, chemical biology, and advanced functional materials.^9^ Among those established synthetic protocols, the methods for formation of C–S bonds *via* functionalization of the C–H bond mainly focused on the C(sp^2^)–H bonds.^10^ The thiolation of C(sp^3^)–H bonds normally relied on the cross-coupling of oxidatively generated radicals with disulfides^11^ or *in situ* generated disulfides.^12^ The disulfides were also applied to the nickel-catalyzed thiolation of β-methyl C(sp^3^)–H bonds of aliphatic carboxamides.^13^ Very recently, Wu reported the first direct use of thiophenol for the allylic C(sp^3^)–H thiolation.^14^ Under visible light irradiation, both the allylic radical and thiyl radical were generated on the surface of photocatalyst quantum dots *via* hydrogen evolution followed by direct radical–radical cross-coupling to afford the allylic C(sp^3^)–H thiolation products.

## 2. Results and discussion

**Scheme 1:**
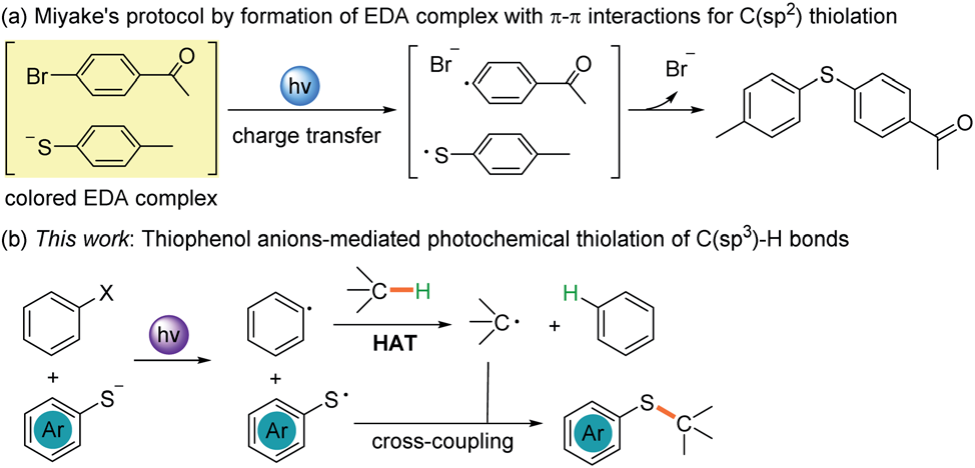
Thiophenolate anions involved in photochemical C(sp^2^) thiolation (a) and C(sp^3^) thiolation (b).

**Fig. 1:**
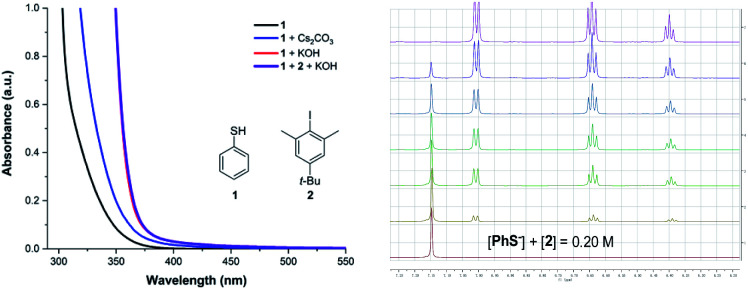
UV-vis absorption spectrum of the thiophenolate anion (left) and ^1^H NMR determination of the binding stoichiometry of the thiophenolate anion–PhI complex (right).

Herein, we report the photochemical etheric, allylic, benzylic, and even cycloalkyl C(sp^3^)–H thiolation *via* direct coupling with thiophenol under mild conditions. Miyake reported that the thiophenolate anion formed an electron donor–acceptor (EDA) complex *via* π–π interactions with aryl halides in the presence of Cs_2_CO_3_ and was excited under visible light irradiation (Scheme 1a).^15^ We discovered that when thiophenol 1 was treated with KOH instead of Cs_2_CO_3_ in THF, the spectrum of the thiophenolate anion showed a significant bathochromic shift with absorption tailing to the 400–450 nm region (Fig. 1a, red line). No significant shift was observed after addition of iodobenzene 2 (Fig. 1a, violet line). Similar results were obtained when the UV-visible absorption spectra were recorded in DMSO (see ESI Note 2†), and they were evidently different from Miyake's spectra of EDA complex formation. However, we observed the formation of a 1 : 1 complex between the thiophenolate anion and 2 with a binding constant *K*_a_ of 1.13 M^−1^ in DMSO-d6 using a ^1^H NMR titration method and a Job's plot analysis (Fig. 1b). We postulated that the thiophenolate anion and PhI formed an EDA complex through halogen bonding interactions^8*c*,16^ instead of π–π interactions^2*b*,25^ in Miyake's case.^15^ Therefore, the photo-excited thiophenolate anion–PhI EDA complex might generate the respective radicals *via* a single electron transfer (SET) process. We proposed that the photoexcited thiophenolate anion–PhI complex would be ideal for the C(sp^3^)–H thiolation by converting the aryl halide to an aryl radical, which could abstract a hydrogen from C(sp^3^)–H to give an alkyl radical.^17^ Then the alkyl radical would cross-couple with a thiyl radical to afford the C(sp^3^)–H thiolation products (Scheme 1b). Furthermore, we reasoned that a bulky aryl halide, such as the 4-*tert*-butyl-2,6-dimethyl-1-iodobenzene 2,^17*j*^ would efficiently prevent the undesired coupling between the aryl radical and thiyl radical, thus avoiding the formation of C(sp^2^)–H thiolation products.^15^

To test the feasibility of our hypothesis, we initiated an investigation by reacting thiophenol 1 and iodobenzene 2 in THF as a model photoreaction. After carefully screening the reaction conditions, we found that upon irradiating the reaction mixture with two 7 W purple LEDs (*λ* = 405 nm) at room temperature for 24 hours with KOH as a base, the thiolation product 3 was obtained in 84% yield with 5% yield of a C(sp^2^)–H thiolation byproduct (Table 1, entry 1). We also performed a 2.0 mmol scale reaction to prove the practicality of the thiolation reaction and product 3 was harvested in 72% yield after 36 hours. The formation of the thiolate anion formed was essential for this transformation because the reaction was completely inhibited in the absence of a base (entry 2). Controlled experiments revealed that no thiolation product formation was detected without light (entry 3), indicating the photochemical nature of this thiolation. The bulky phenyl iodide 2 was found to be the ideal electron acceptor while iodobenzene or 4-iodoanisole afforded lower yields with generation of the C(sp^2^)–H thiolation byproducts (8% and 12% yields of the byproduct formed, respectively, entries 4 and 5). Other bases, such as NaOH, CsOH·*x*H_2_O, KH, Cs_2_CO_3_, K_2_CO_3_, DBU, and TMG, were also evaluated (entries 6–12). It was found that NaOH was also an efficient base for the photochemical thiolation, albeit with a little lower yield. CsOH·*x*H_2_O only delivered the product in 28% yield. When KH was used instead of KOH, similar yield was obtained. As shown in Fig. S5 of ESI Note 2,† no significant bathochromic shift was observed in the presence of K_2_CO_3_. And we did not identify any thiolation products when the reaction mixture containing K_2_CO_3_ was irradiated under purple light (entry 10). The organic bases resulted in low yields too.

**Table 1:** Optimization of the photochemical thiolation conditions

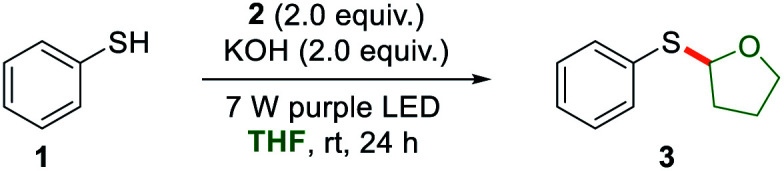		
Entry^a^	Variation from the reaction conditions	Yield^b^ [%]
1	None	84
2	Without base	n.r.
3	Without light	n.r.
4	Iodobenzene	66
5	4-Iodoanisole	78
6	NaOH	75
7	CsOH·*x*H_2_O	28
8	KH	81
9	Cs_2_CO_3_	43
10	K_2_CO_3_	n.r.
11	DBU	34
12	TMG	28

a Reaction conditions: a mixture of thiophenol 1 (0.1 mmol), 4-(*tert*-butyl)-iodo-2,6-dimethyliodobenzene 2 (0.2 mmol), and KOH (0.2 mmol) in 1.0 mL of THF was irradiated with two 7 W purple LEDs for 24 h at room temperature.

b Isolated yield. n.r. = no reaction.

**Scheme 2:**
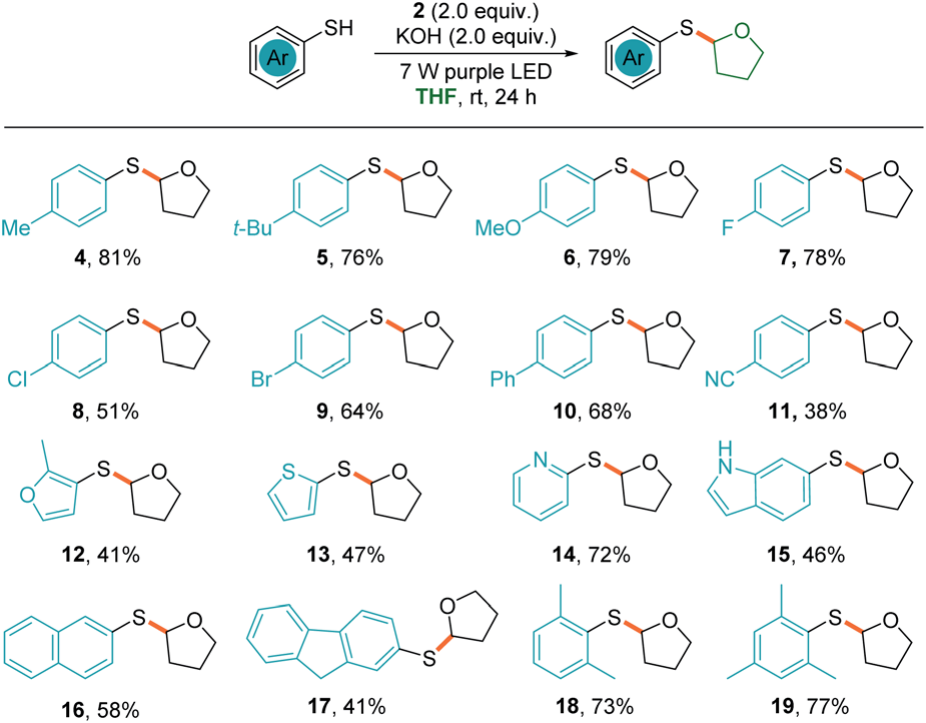
Substrate scope of thiophenols. Reaction conditions: a mixture of thiophenol (0.1 mmol), 2 (0.2 mmol), and KOH (0.2 mmol) in 1.0 mL of THF was irradiated with two 7 W purple LEDs for 24 h at room temperature.

With the optimized reaction conditions in hand, we sought to evaluate the substrate scope of the thiophenolate anion induced photochemical thiolation. Different arylthiols were first examined as shown in Scheme 2. The thiophenols with different electronic nature of substitution patterns were found to be compatible and afforded the corresponding products in moderate to good yields. Under the standard conditions, arylthiols with electron-donating groups, such as alkyl and alkoxy substituted thiophenols, gave the related products in good yields (4–6). Halogenated thiophenols were also tolerated and afforded thiolation products in 51–78% yields (7–9). In addition, moderate yield was obtained when there is a phenyl substitution (10). It was discovered that the cyano group was not compatible for this thiolation and only 38% yield was delivered (11). Subsequently, several heteroarylthiols were exploited for the photochemical thiolation and gave the corresponding products in 41–72% yields (12–15). We next evaluated polycyclic thiophenols and found that naphthalene (16) and fluorene (17) were efficient coupling partners. In addition, the *ortho*-steric substitutions on the thiophenols did not affect the cross-coupling reaction (18 and 19).

**Scheme 3:**
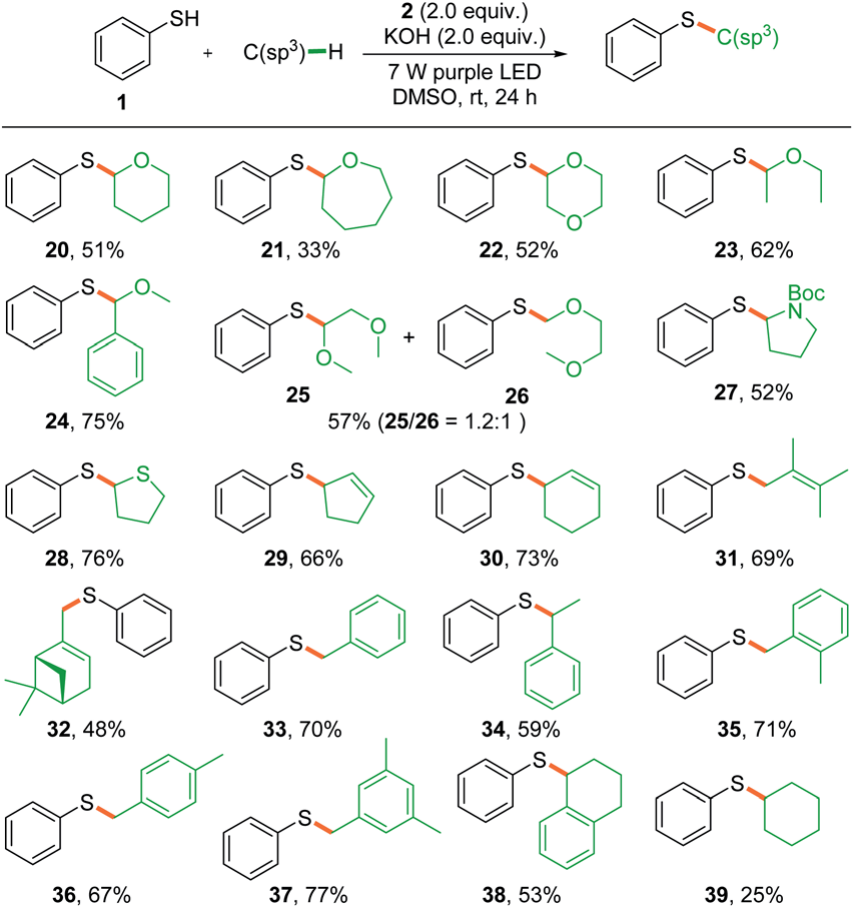
Substrate scope for C(sp^3^)–H bond activation. Reaction conditions: a mixture of thiophenol 1 (0.1 mmol), 2 (0.2 mmol), C(sp^3^)–H bond-containing substrate (1.0 mmol), and KOH (0.2 mmol) in 1.0 mL of DMSO was irradiated with two 7 W purple LEDs for 24 h at room temperature.

To explore the scope of the photochemical thiolation in cross-coupling with different C(sp^3^)–H bond-containing substrates, alternative solvents instead of THF should be exploited. We then performed the photoreaction in DMSO, DMF, MeCN, and acetone with 10 eq. of THF as a substrate. The experimental results showed that 65% yield was delivered in DMSO, while 13–27% yields were obtained in other solvents. As shown in Scheme 3, a series of ether compounds were first evaluated for the thiolation (20–26). Moderate yields were obtained for most of the ether compounds except the oxepane-derived product (21). We observed excellent regioselectivity for benzyl methyl ether (24) where the bond dissociation energies (BDEs) of two different C(sp^3^)–H bonds are significantly different. However, both of the two thiolation products 25 and 26 were generated when the 1,2-dimethoxyethane was subjected to photoreaction. Next, pyrrolidine and tetrahydrothiophene were also tested for the reaction, and thiolation products were obtained in moderate yields (27 and 28).

The direct allylic C(sp^3^)–H thiolation is found to be difficult for the transition-metal catalyzed π-allyl metal pathway.^18^ Wu's pioneering photochemical radical–radical cross-coupling pathway provided an efficient method for the direct allylic C–H thiolation.^14^ We conjectured that the *in situ* generated phenyl radical would also be efficient in abstracting a hydrogen from allylic C(sp^3^)–H to give the allylic radical and enable direct allylic thiolation. Several substrates with allylic C(sp^3^)–H, such as cyclopentene, cyclohexene, 2,3-dimethyl-2-butene, and β-pinene, were tested under the standard conditions and the corresponding thiolation products were obtained in moderate yields (29–32). Moreover, the toluene derivatives were also proved to be suitable substrates for the radical–radical cross-coupling thiolation (33–38). Owing to the high BDEs, this method was found to be not effective in the thiolation of alkanes and only 25% yield was delivered for the cyclohexane (39).

**Scheme 4:**
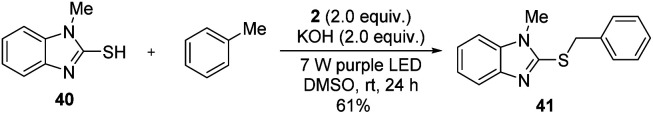
The photochemical synthesis of thiobenzimidazole.

The generated thiolation products, such as compound 3, are widely used in allylation, ketonylation, and other chemical conversions. To show the utility of the photochemical coupling, we also synthesized thiobenzimidazole, a selective CRTh2 receptor antagonist.^19^ As shown in Scheme 4, irradiation of the complex of thiol 40 with toluene in the presence of iodide 2 under basic conditions for 24 h efficiently delivered the target product 41 in 61% yield in one step.

**Scheme 5:**
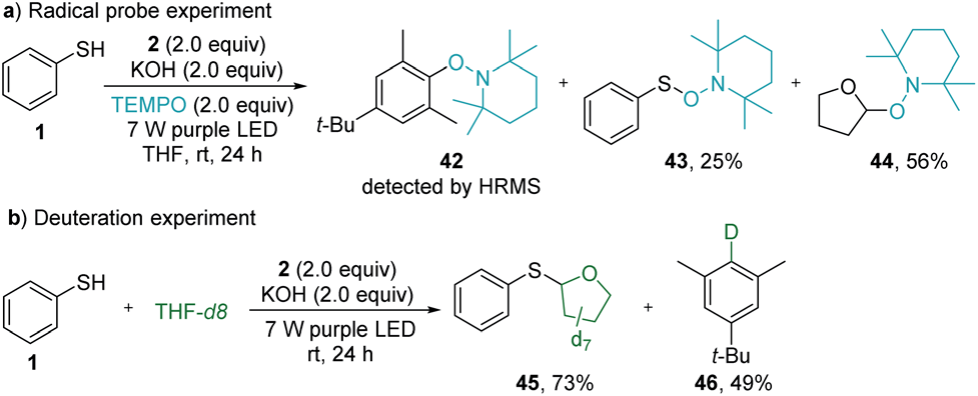
Controlled experiments. (a) Trapping radical intermediates with TEMPO. (b) Deuteration experiment for verifying the hydrogen atom transfer process.

The radical pathway of the photochemical thiolation was confirmed by the controlled experiments (Scheme 4). When the radical scavenger TEMPO was added to the reaction mixture under standard reaction conditions, the thiolation was completely inhibited. Instead, the TEMPO-trapped compounds 42–44 were detected from the reaction (Scheme 5a), indicating the formation of the phenyl radical, thiophenolic radical, and the etheric radical. To verify that the etheric radical was generated through phenyl radical-mediated hydrogen atom transfer (HAT), a deuteration experiment was performed. The isolation of deuterated arene 46 undeniably validated this process (Scheme 5b). The quantum yield for the model reaction was determined to be *Φ* = 0.23 (see ESI Note 7†), indicating that the reported thiolation reaction was a non-chain process^20^ or an inefficient chain process.^21^

**Fig. 2:**
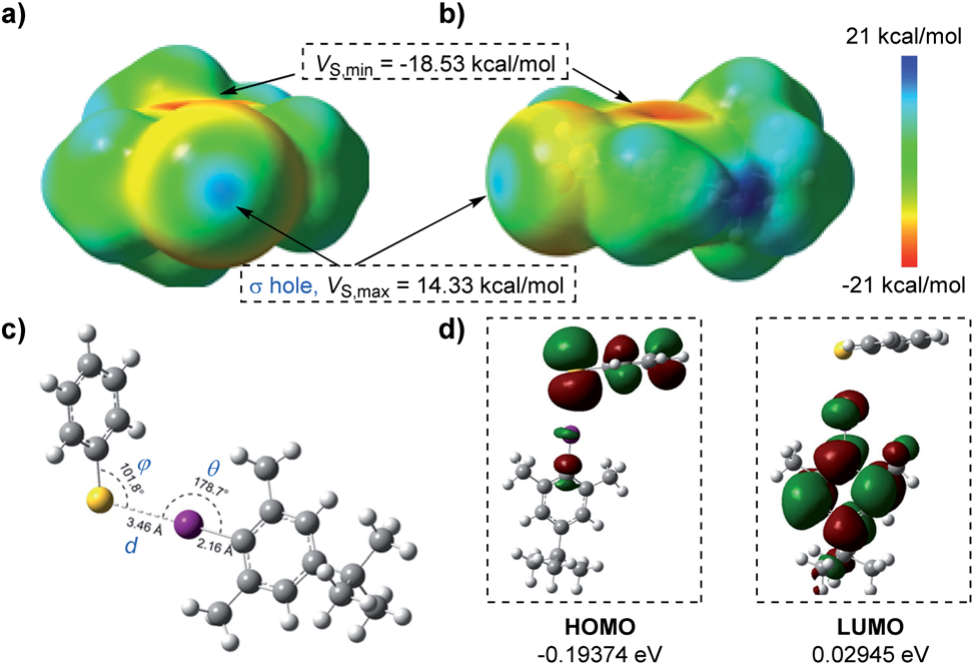
(a) and (b) Electrostatic potentials mapped on the molecular surface (electron density *ρ* = 0.001 electrons per bohr^3^, kcal mol^−1^) calculated at the M06-2X/def2-TZVP level; the iodine atom is pointing out of the page (a) and to the left (b). (c) Calculated geometries, bond distances, and bond angles of the thiophenolate anion and aryl iodide complex. (d) Calculated highest occupied molecular orbitals (HOMOs) and lowest unoccupied molecular orbitals (LUMOs) of the EDA complex.

To better visualize the interactions of the thiophenolate anion and aryl iodide complex, we carried out DFT calculations as shown in Fig. 2. The electrostatic potential surfaces (ESPs) of the iodine atom contained a positive area along the extension of the Ar–I bond that was surrounded by negative electrostatic potential with a distance of 2.19 Å as mapped in GaussView 6.0.16 (ref. 22) (ESI Fig. 10†), indicating the existence of a “σ-hole” (Fig. 2a and b).^4*a*^ This region was necessary for the formation of halogen bonding with negative parts of the thiophenolate anion. We noticed that the most positive value of electrostatic potential (*V*_s,max_, 14.33 kcal mol^−1^) of the “σ-hole”^23^ was a reliable indicator for analyzing and predicting the character of a non-covalent bond.^24^ Its sign determined whether the positive contribution of nuclei or the negative contribution of electrons was dominant in any area of space. Since there were three parameters, *d*, *θ*, and *φ*, which were essential in the properties of halogen bonding, we subsequently conducted geometry optimization and interaction energy calculations of the complex intermediate. In the equilibrium structure shown in Fig. 2c, the S^−^⋯I interactions had a distance *d* of 3.46 Å which was shorter than the sum of van der Waals radii of the two interacting atoms (3.78 Å).^25^ Thus the S^−^⋯I interactions should be responsible for the production of the EDA complex. The calculated angle *θ* of Ar–I⋯S was 178.7°, indicating that the interactions were highly directional along the extension of the covalent Ar–I bond and were in good agreement with the nature of halogen bonding.^4*a*^ The binding energy calculated was −8.61 kcal mol^−1^ (Table S1 in the ESI†) which demonstrated that the interaction of the thiophenolate anion and aryl iodide complex was relatively stable. The lower HOMO–LUMO energy gaps (0.22319 eV) revealed that the electron transfer process occurred favourably under visible light irradiation (Fig. 2d).

At the same time, the electrostatic potential over the aromatic ring was found to be extremely negative (*V*_s_,_min_ = −18.53 kcal mol^−1^) (Fig. 2a and b). These results implied that the iodobenzene 2 was not suited for establishing interactions with the thiophenolate anion (*V*_s,min_ = −124.17 kcal mol^−1^) (Fig. S10 in the ESI†) *via* a π–π system where the existence of both electron-rich and electron-deficient π systems was necessary,^2*b*,15,26^ or for establishing anion–π interactions.^27^ Moreover, the possible hydrogen bonding was also excluded due to the fact that no reactions were observed with the weak base Cs_2_CO_3_ (ESI Note 1†). Meanwhile, the attempted optimization for conformations of possible π–π interactions, anion–π interactions, or σ-type interactions indeed led to dissociation of the complex due to the mutual electrostatic repulsion of the two negative systems.

In order to elaborate on the interactions more clearly, we performed a Quantum Theory of Atoms in Molecules (QTAIM) analysis,^28^ which has been widely utilized to determine the bond critical points (BCPs) of halogen bonding interactions.^27*a*,29^ The properties at the BCPs provide a lot of information that can be used to characterize the chemical structure and nature of chemical bonds. From QTAIM calculations performed using the Multiwfn 3.8 package,^30^ we found the existence of BCPs that had two negative and one positive eigenvalues of Hessian (Table S2 in the ESI†). Based on the relatively low value of electron densities *ρ* and the positive value of Laplacian of electron density ∇^2^*ρ* at BCPs (Table 2), this kind of halogen bond can be classified as a “closed-shell” bond. Furthermore, the positive sign of energy density *H* meant that the interactions were not covalent but dominantly electrostatic. Additionally, the absolute value for the ratio of eigenvalues of Hessian *λ*_1_ and *λ*_3_, and that of potential energy density *V* and Lagrangian kinetic energy *G* were less than 0.25 and 1, respectively. These results illuminated explicitly the fact that the interactions of the thiophenolate anion and aryl iodide complex were closed-shell non-covalent interactions.

**Table 2:** M06-2X/def2-TZVP calculated QTAIM topological parameters at the BCPs of thiophenolate anion and aryl iodide interactions^a^

Interactions	*ρ*	∇^2^*ρ*	*V*	*H*	*G*
S^−^⋯I	0.0134	0.0326	−0.0067	0.0007	0.0074

a BCPs = bond critical points. *ρ* = electron density. ∇^2^*ρ* = Laplacian of electron density. *V* = potential energy density. *H* = energy density. *G* = Lagrangian kinetic energy. *ρ*, ∇^2^*ρ*, *H*, *V*, and *G* are in atomic units.

**Scheme 6:**
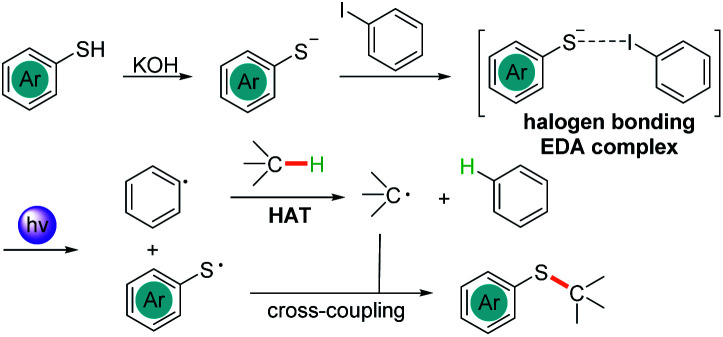
Proposed photochemical mechanism.

On the basis of the above results, we proposed the mechanism of photoexcited C(sp^3^)–H bond activation and thiolation as depicted in Scheme 6. By deprotonation with KOH, the generated thiophenolate anion formed an EDA complex with iodobenzene 2*via* halogen bonding interactions. The photoexcited EDA complex underwent a SET process to afford two radicals, a thiyl radical and aryl radical, as shown in Scheme 6. The aryl radical then abstracted a hydrogen atom from the C(sp^3^)–H in the HAT pathway to deliver the alkyl radical. Finally, the radical–radical cross-coupling between the thiyl radical and the alkyl radical achieved the C(sp^3^)–H thiolation products.

## 3. Conclusions

In summary, we discovered that the thiophenolate anion and iodobenzene could form an EDA complex through halogen bonding interactions and be excited under visible light irradiation. A transition-metal-free photochemical thiolation of C(sp^3^)–H bond-containing compounds with thiophenol was successfully developed. Mechanistic studies indicated that the excited thiolate anion–PhI EDA complex underwent SET to generate a thiyl radical and aryl radical followed by a HAT between the aryl radical and C(sp^3^)–H bond-containing substrates to give a C(sp^3^) centred-radical. The radical–radical cross-coupling between the thiyl radical and C(sp^3^)-centred radical delivered thiolation products with high structural diversity.

## Data availability

All relevant experimental and computational details are provided in the ESI.†

## Author contributions

T. Li conducted most of the experiments and wrote the initial manuscript draft. K. Liang, J. Tang, and X. Tong performed some of the experiments. Y. Ding performed the DFT calculations. C. Xia conceptualized and directed the project and finalized the manuscript draft. All authors contributed to discussions. We are grateful for the computational resources provided by the High-Performance Computing Platform of Yunnan University.

## Conflicts of interest

There are no conflicts to declare.

## Supplementary Material

SC-012-D1SC03667J-s001
