# Correction: A photoexcited halogen-bonded EDA complex of the thiophenolate anion with iodobenzene for C(sp^3^)–H activation and thiolation

**DOI:** 10.1039/d1sc90238e

**Published:** 2021-11-23

**Authors:** Tao Li, Kangjiang Liang, Jiaying Tang, Yuzhen Ding, Xiaogang Tong, Chengfeng Xia

**Affiliations:** Key Laboratory of Medicinal Chemistry for Natural Resource, Ministry of Education, Yunnan Provincial Center for Research & Development of Natural Products, School of Chemical Science and Technology, Yunnan University Kunming 650091 China xiacf@ynu.edu.cn

## Abstract

Correction for ‘A photoexcited halogen-bonded EDA complex of the thiophenolate anion with iodobenzene for C(sp^3^)–H activation and thiolation’ by Tao Li *et al.*, *Chem. Sci.*, 2021, DOI: 10.1039/d1sc03667j.

Upon publication of the original article, the authors became aware of another work in this area that reported a related transformation, given below as ref. 1. This reference should also be considered when reading the original *Chemical Science* article.

The Royal Society of Chemistry apologises for these errors and any consequent inconvenience to authors and readers.

## Supplementary Material
