# Supplementary material for: A photoexcited halogen-bonded EDA complex of the thiophenolate anion with iodobenzene for C(sp3)–H activation and thiolation
Source: Chem Sci. 2021 Oct 21;12(47):15655–61. doi: 10.1039/d1sc03667j (PMC8654056; doi:10.1039/d1sc03667j)

Electronic Supplementary Material (ESI) for Chemical Science.
This journal is © The Royal Society of Chemistry 2021

Supplementary Information

A Photoexcited Halogen Bonding EDA Complex of Thiophenolate Anion with Iodobenzene for C(sp^3)–H Activation and Thiolation

Tao Li, Kangjiang Liang, Jiaying Tang, Yuzhen Ding, Xiaogang Tong, and Chengfeng Xia*

Key Laboratory of Medicinal Chemistry for Natural Resource, Ministry of Education, Yunnan Provincial Center for Research & Development of Natural Products, School of Chemical Science and Technology, Yunnan University, Kunming 650091, China

Table of Contents

General Information S2
General Procedure for Photochemical Thiolation S2
Supplementary Note 1. ^{1}H NMR Spectroscopic Studies S5
Supplementary Note 2. UV-Vis Spectroscopic Measurements S6
Supplementary Note 3. Determination of Binding Stoichiometry S9
Supplementary Note 4. Determination of the Association Constant (K_a). S11
Supplementary Note 5. Radical Trapping Experiments S12
Supplementary Note 6. Deuterium Labelling Experiments S14
Supplementary Note 7. Quantum Yield Measurements S15
Supplementary Note 8. Density Functional Theory Calculations S18
Identification of Compounds S25
References S43
Copies of NMR Spectra S45

General Information

^{1}H and ^{13}C NMR were recorded on a Bruker Avance 600 MHz spectrophotometer. Chemical shifts (δ) are expressed in ppm., and *J*-values are given in Hz. The residual solvent protons (^{1}H) or the solvent carbons (^{13}C) were used as internal standards. HR-ESI-MS were taken on Agilent 6540 Q-TOF spectrometer. All reagents were used as received without further purification unless indicated otherwise. 405 nm LED lamps (7 W) were used to irradiate the reaction mixtures. Thin layer chromatographies were carried out on GF254 plates. Flash chromatography was performed with 200-300 mesh silica gels. Visualization of the developed chromatogram was performed by fluorescence quenching or by ceric ammonium molybdate, or $KMnO_4$ stain. UV-Vis measurements were carried out on a Hitachi UV-1900 UV-Visible spectrophotometer. Cyclic voltammetry studies were carried out on a CHI 760E electrochemical workstation (Shanghai CH Instruments Co., China). Optical rotations were recorded on a JASCO P-2000 polarimeter.

General Procedure for Photochemical Thiolation

General Procedure A

To an oven dried 10 mL glass tube with a magnetic stirring bar was added thiophenol **1** (0.10 mmol), KOH (0.20 mmol), 5-(*t*ert-butyl)-2-iodo-1,3-dimethylbenzene **2** (0.20 mmol) and tetrahydrofuran (1.0 mL) under an argon atmosphere in a glove box. The reaction mixture was stirred under two 7 W 405 nm light emitting diode (LED) lamps (the distance was about 10 cm) irradiation at room temperature. After 24 hours, the reaction was quenched by water (15 mL), extracted with ethyl acetate (15 mL × 3). The combined organic layers were washed with brine, dried over anhydrous Na_2SO_4 and concentrated. The crude product was subjected to column chromatography (dichloromethane/petroleum ether) or (ethyl

acetate/petroleum ether) on silica gel to afford the product.

General Procedure B

To an oven dried 10 mL glass tube with a magnetic stirring bar was added thiophenol **1** (0.10 mmol), tetrahydropyran (1.00 mmol), KOH (0.20 mmol), 5-(*tert*-butyl)-2-iodo-1,3-dimethylbenzene **2** (0.20 mmol) and DMSO (1.0 mL) under an argon atmosphere in a glove box. The reaction mixture was stirred under two 7 W 405 nm light emitting diode (LED) lamps (the distance was about 10 cm) irradiation at room temperature. After 24 hours, the reaction was quenched by water (15 mL), extracted with ethyl acetate (15 mL × 3). The combined organic layers were washed with brine, dried over anhydrous Na_2SO_4 and concentrated. The crude product was subjected to column chromatography (dichloromethane/petroleum ether) or (ethyl acetate/petroleum ether) on silica gel to afford the product.

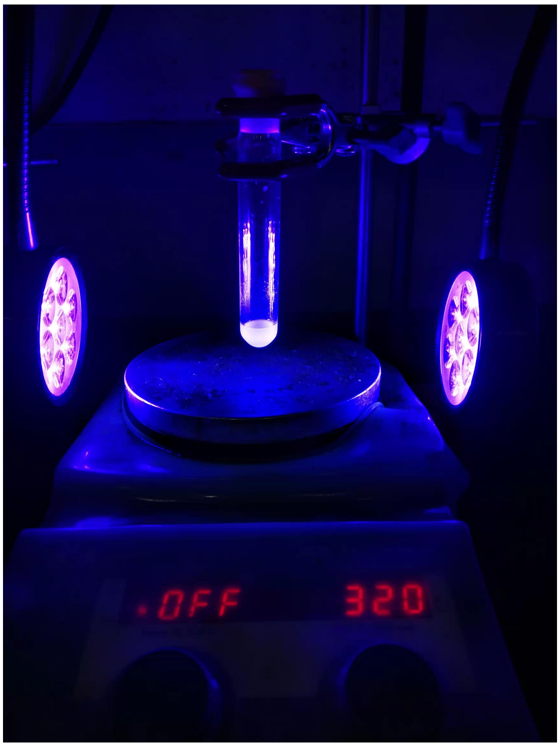

Supplementary Figure 1. Experimental setup

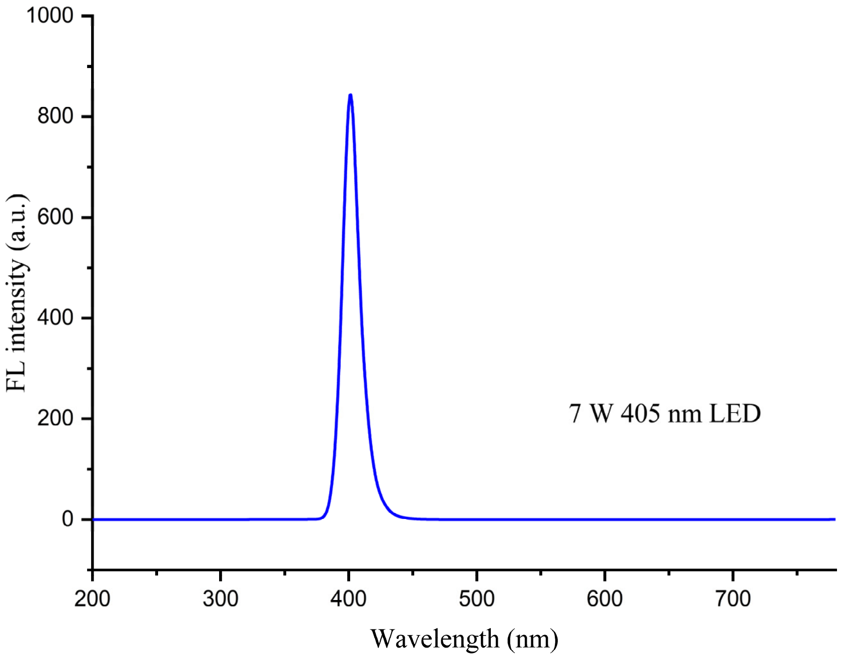

Supplementary Figure 2. Emission spectra of the 7 W 405 nm LED lamp.

Supplementary Note 1. ^{1}H NMR Spectroscopic Studies

The ^{1}H NMR analysis was made on a solution containing **1** (5.5 mg, 0.05 mmol)**,** mixtures of **1** (5.5 mg, 0.05 mmol) and Cs_2CO_3 (32.6 mg, 0.1 mmol), and mixtures of **1** (5.5 mg, 0.05 mmol) and KOH (5.6 mg, 0.1 mmol) in 0.5 mL of THF-d_8. Under these conditions, **1** was completely deprotonated by KOH and significant upfield peak shifting of hydrogens were observed. Meanwhile, we also discovered that the Cs_2CO_3 was not basic enough to deprotonate the thiophenol. Therefore, we conjectured that the formation of EDA complex was necessary to facilitate the electron transfer and a proton-coupled electron transfer (PCET) process was involved in Miyake's protocol.

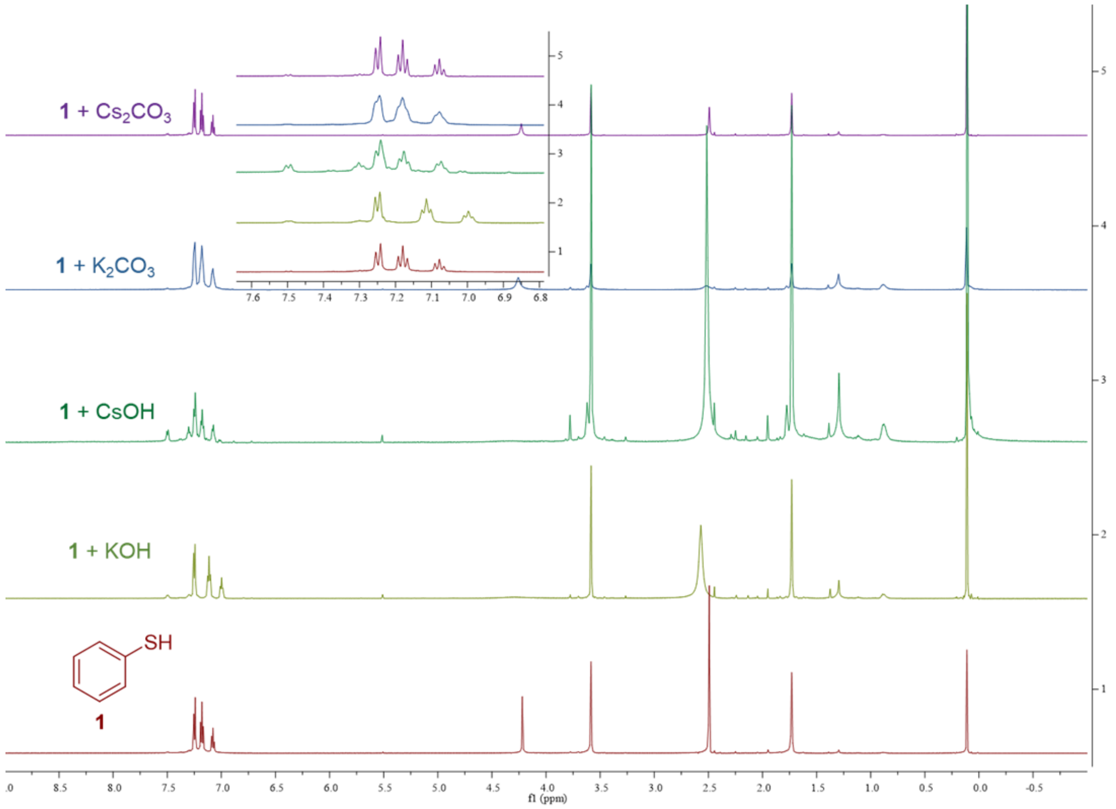

Supplementary Figure 3. Comparison of ^{1}H NMR spectra of **1** and the thiolate anion of **1** in THF-d_8.

Supplementary Note 2. UV-Vis Spectroscopic Measurements

1. The UV-Vis spectroscopic studies excluding the formation of EDA complex

The UV-Vis absorption spectra of THF solutions (0.1 M) of **1**, **2**, mixtures of **1** and KOH, and mixtures of **1**, **2** and KOH were recorded on Hitachi UV-1900 UV-Visible spectrophotometer (1 mm short light path cuvettes have been employed in order to avoid fast signal saturation). The colorless solution of thiophenol **1** (orange line) has no color change upon addition of KOH (blue line) and no new color change after the 5-(*t*ert-butyl)-2-iodo-1,3-dimethylbenzene **2** was added to the solution of the thiolate anion of **1** (red line).

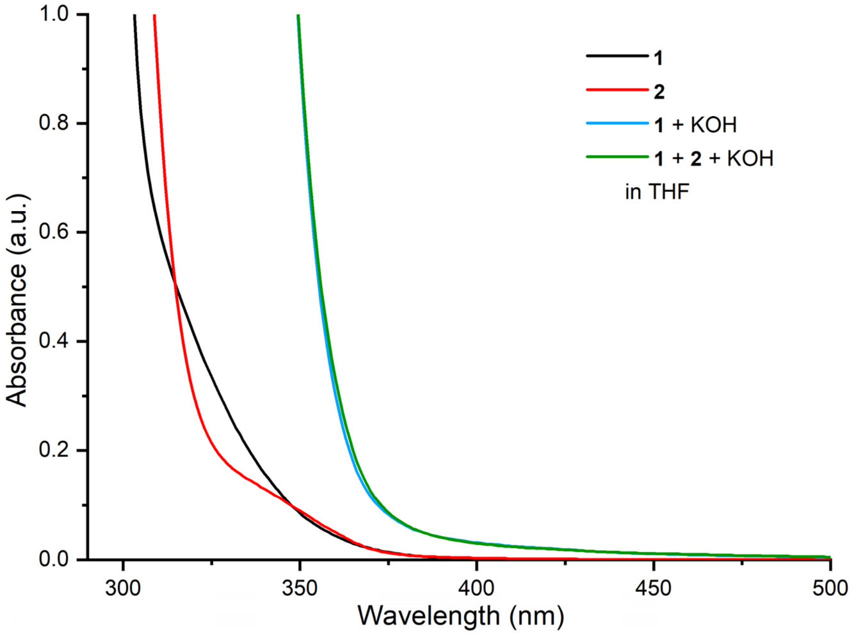

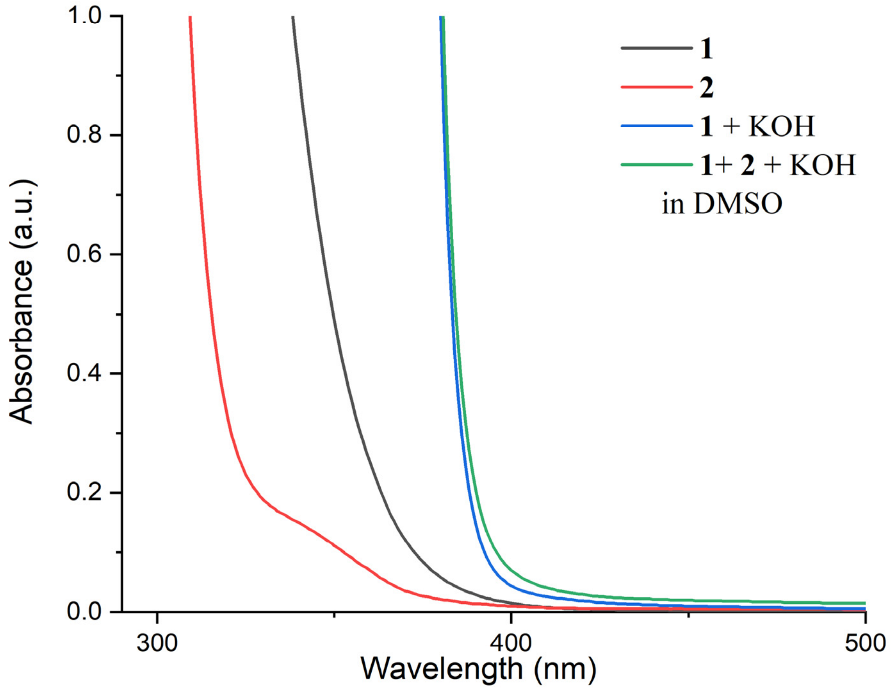

Supplementary Figure 4. UV-Vis absorption spectra of mixtures of **1**, **2**, and KOH in THF (top) and DMSO (bottom) at concentrations of 0.1 M.

2. The UV-Vis absorption spectra of thiophenol under the treatment of different bases

The UV-Vis absorption spectra of THF solutions (0.1 M) of thiophenol **1** mixed with different bases were recorded on Hitachi UV-1900 UV-Visible spectrophotometer (1 mm short light path cuvettes have been employed in order to avoid fast signal saturation).

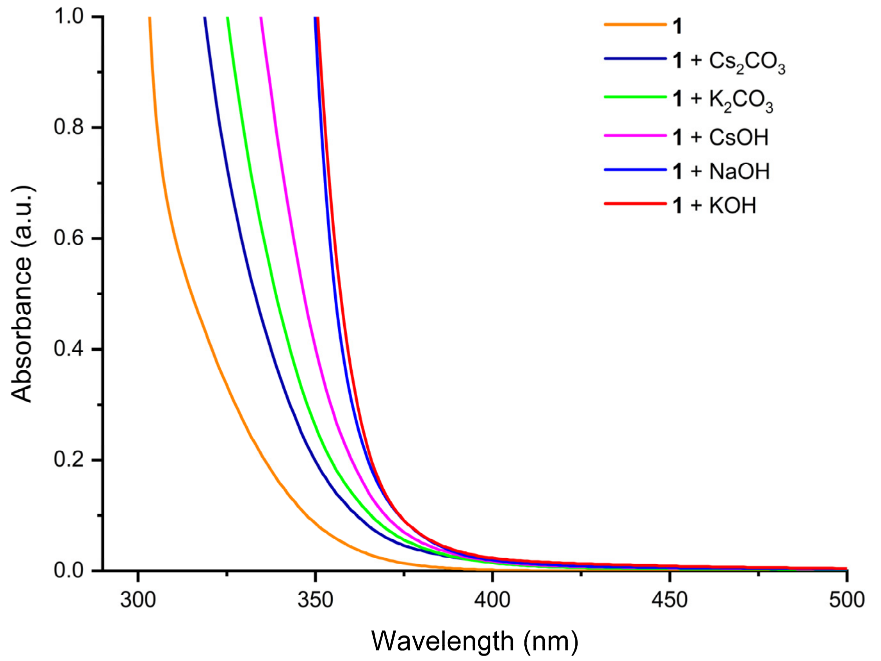

Supplementary Figure 5. UV-Vis absorption spectra of mixtures of **1** and different bases in THF at concentrations of 0.1 M.

Supplementary Note 3. Determination of Binding Stoichiometry

The binding stoichiometry between thiolate anion of **1** and **2** were evaluated using Job's plot analysis. ^{1}H NMRspectra of seven samples of mixtures of thiolate anion of **1** and **2** in DMSO-d6 were recorded at 298 K. DMSO (δ = 2.50 ppm.) was used as internal standard. The total volume of the mixture was 0.5 mL, and the total amount of thiolate anion of **1** and **2** was kept constant at 0.1 mmol (0.2 M), while the amount of thiolate anion of **1** was varied from 0 to 0.1 mmol (0 - 0.2 M). The molar ratios of thiolate anion of **1**/ (thiolate anion of **1** + **2**) were 0.0, 0.2, 0.4, 0.5, 0.6, 0.8, 0 1.0. ^{1}H NMR for each sample was recorded and the chemical shifts differences (Δδ) for *p*-H of thiolate anion of **1** were used to draw the plot. The stoichiometry was determined by plotting ratios of [thiolate anion of **1**] × Δδ against ratios of [thiolate anion of **1**]/ [thiolate anion of **1** + **2**] to afford a maximum at ratio [thiolate anion of **1**]/ [thiolate anion of **1** + **2**] ≈ 0.5, which meant a 1:1 complex ratio between thiolate anion of **1** and **2**.

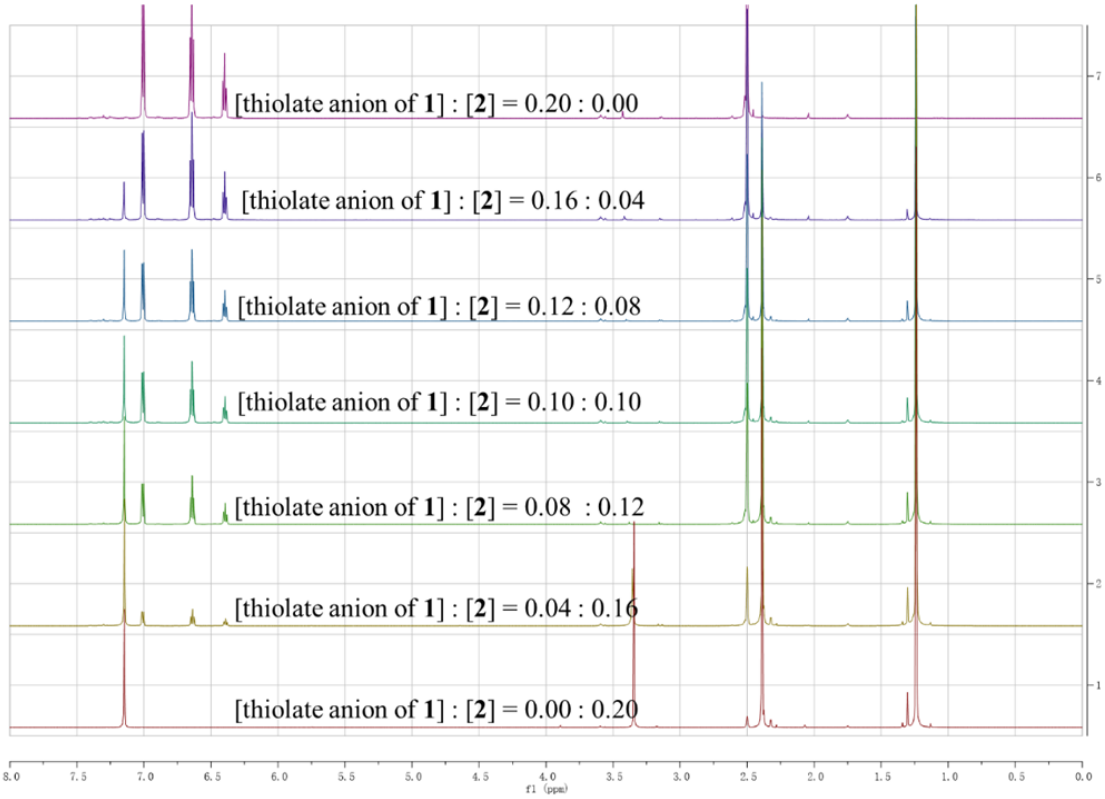

Supplementary Figure 6. ^{1}H NMR shift of thiolate anion of **1** with **2**.

	[thiolate anion of **1**] (M)	Δδ (ppm)	[thiolate anion of **1**]/ [thiolate anion of **1** + **2**]	[thiolate anion of **1**] × Δδ
1	0	0	0	0
2	0.04	0.00917	0.2	0.0003668
3	0.08	0.00554	0.4	0.0004432
4	0.10	0.00406	0.5	0.000406
5	0.12	0.00296	0.6	0.0003552
6	0.16	0.00133	0.8	0.0002128
7	0.20	0	1.0	0

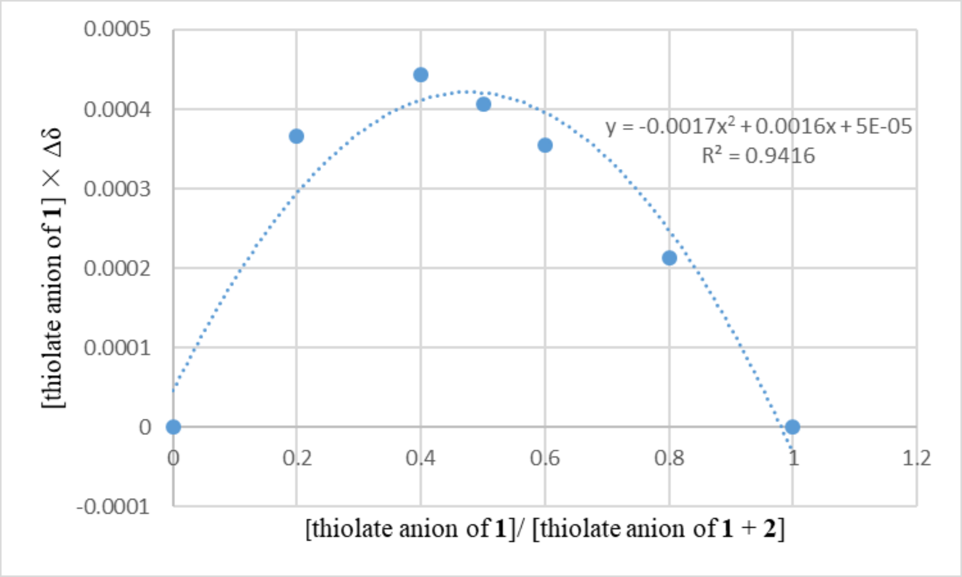

Supplementary Figure 7. Job's plot analysis. $y = -0.0017x^2 + 0.0016x + 5E\text{-}05$, $x_{max} = 0.0016/(-2\times(-0.0017)) \approx 0.5$.

Supplementary Note 4. Determination of the Association Constant (K_a).

In an NMR tube, a DMSO-d6 solution of thiolate anion of **1** (50 mM, 0.6 mL, 0.03 mmol) was added at 298 K. To the tube, **2** was added from 0.1 to 0.34 mmol. ^{1}H NMR for each sample was recorded to measure the change in chemical shift for the *p*-H of thiolate anion of **1**. DMSO was used as internal standard and δ = 2.50 ppm.

	[**2**] (mol/L)	1/[**2**]	Δδ (ppm)	1/Δδ
1	0.34	2.9	0.01500	66.67
2	0.25	4.0	0.00976	102.46
3	0.2	5.0	0.00806	124.67
4	0.15	6.7	0.00639	156.49
5	0.1	10	0.00481	207.90

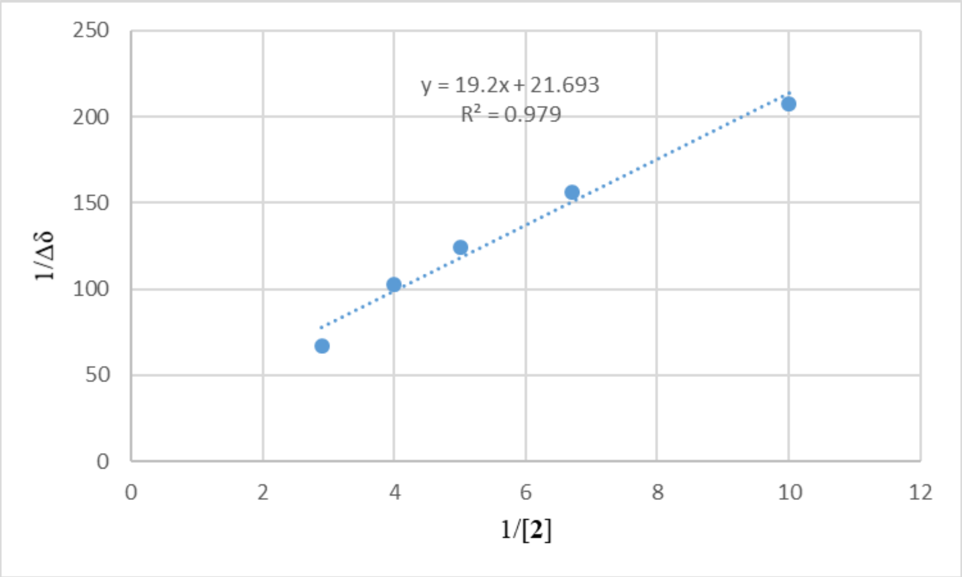

Supplementary Figure 8. Association constant determination.

$$Ka = \frac{\text{intercept}}{slope} = \frac{21.693}{19.200} = 1.13\ M^{-1}$$

The association constant between thiolate anion of **1** and **2** was calculated to be 1.13 M^{-1} in DMSO-d6.

Supplementary Note 5. Radical Trapping Experiments

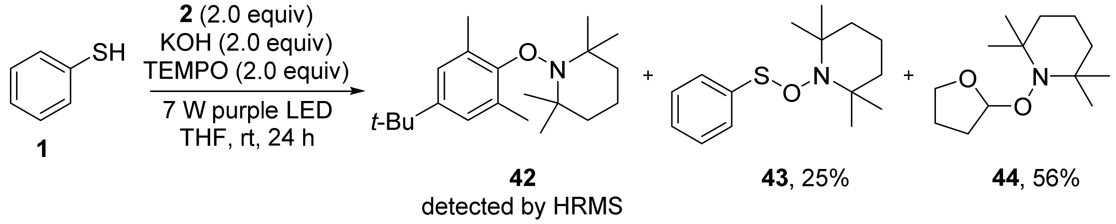

To an oven dried 10 mL glass tube with a magnetic stirring bar was added thiophenol **1** (11.0 mg, 0.10 mmol), KOH (11.2 mg, 0.20 mmol), 5-(*t*ert-butyl)-2-iodo-1,3-dimethylbenzene **2** (57.6 mg, 0.20 mmol), TEMPO (31.2 mg, 0.20 mmol) and tetrahydrofuran (1.0 mL) under an argon atmosphere in a glove box. The reaction mixture was stirred under two 7 W 405 nm light emitting diode (LED) lamps (the distance was about 10 cm) irradiation at room temperature. After 24 hours, the reaction was quenched by water (15 mL), extracted with ethyl acetate (15 mL × 3). The combined organic layers were washed with brine, dried over anhydrous Na_2SO_4 and concentrated. The crude product was subjected to column chromatography (ethyl acetate/petroleum ether = 1:10) on silica gel to afford **43** (6.6 mg, 25% yield) and **44** (12.6 mg, 56% yield).

1-(4-(*tert*-Butyl)-2,6-dimethylphenoxy)-2,2,6,6-tetramethylpiperidine (42)

HR-ESI-MS (m/z): calcd. for $C_{21}H_{36}NO$ $[M + H]^+$: 318.2791, found 318.2788.

2,2,6,6-Tetramethyl-1-((phenylthio)oxy)piperidine (43)[1]

^{1}H NMR (600 MHz, $CDCl_3$) δ 7.72 – 7.64 (m, 2H), 7.48 – 7.42 (m, 2H), 7.41 – 7.36 (m, 1H), 1.90 – 1.81 (m, 1H), 1.66 (s, 5H), 1.63 – 1.58 (m, 2H), 1.55 –1.35(m, 7H), 0.90 (s, 3H).

^{13}C NMR (150 MHz, $CDCl_3$) δ 150.5, 129.4, 128.7, 126.2, 61.5, 59.0, 43.6, 41.6, 35.5, 32.8, 28.9, 28.1, 27.8, 17.4.

HR-ESI-MS (m/z): calcd. for $C_{15}H_{24}NOS$ $[M + H]^+$: 266.1573, found 266.1573.

2,2,6,6-Tetramethyl-1-((tetrahydrofuran-2-yl)oxy)piperidine (44)[2]

^{1}H NMR (600 MHz, $CDCl_3$) δ 5.35 (d, J = 4.8 Hz, 1H), 3.89 – 3.80 (m, 2H), 2.01 – 1.89 (m, 3H), 1.81 – 1.75 (m, 1H), 1.56 – 1.44 (m, 5H), 1.31 (d, J = 12.8 Hz, 1H),

1.21 (s, 3H), 1.10 (s, 3H), 1.06 (s, 3H), 1.03 (s, 3H).

^{13}C NMR (150 MHz, $CDCl_3$) δ 109.8, 66.8, 60.3, 58.8, 40.3, 39.9, 34.1, 33.5, 31.4, 24.1, 20.6, 20.2, 17.4.

HR-ESI-MS (m/z): calcd. for $C_{16}H_{26}NO_2$ $[M + H]^+$: 228.1958, found 228.1955.

Supplementary Note 6. Deuterium Labelling Experiments

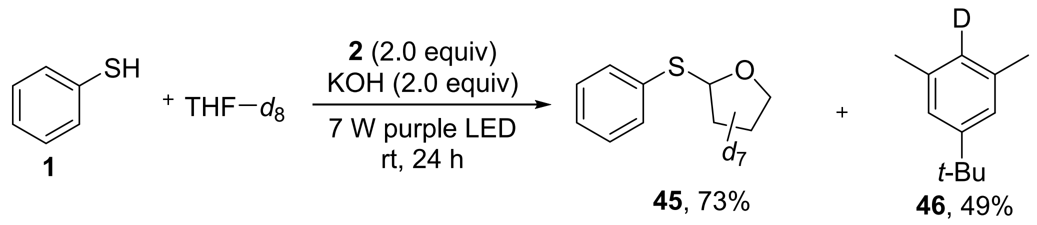

To an oven dried 10 mL glass tube with a magnetic stirring bar was added thiophenol **1** (11.0 mg, 0.10 mmol), KOH (11.2 mg, 0.20 mmol), 5-(*t*ert-butyl)-2-iodo-1,3-dimethylbenzene **2** (57.6 mg, 0.20 mmol) and tetrahydrofuran-d_8 (1.0 mL) under an argon atmosphere in a glove box. The reaction mixture was stirred under two 7 W 405 nm light emitting diode (LED) lamps (the distance was about 10 cm) irradiation at room temperature. After 24 hours, the reaction was quenched by water (15 mL), extracted with ethyl acetate (15 mL × 3). The combined organic layers were washed with brine, dried over anhydrous Na_2SO_4 and concentrated. The crude product was subjected to column chromatography (dichloromethane/petroleum ether = 1:2) on silica gel to afford **45** (13.6 mg, 73% yield) and **46** (14.3 mg, 49% yield).

2-(Phenylthio)tetrahydrofuran-2,3,3,4,4,5,5-d_7 (45)

^{1}H NMR (600 MHz, $CDCl_3$) δ 7.53 – 7.48 (m, 2H), 7.30 (t, J = 7.7 Hz, 2H), 7.24 – 7.21 (m, 1H).

^{13}C NMR (150 MHz, $CDCl_3$) δ 135.8, 131.3, 129.0, 126.9.

HR-EI-MS (m/z): calcd. for $C_{10}H_5D_7OS$ [M]: 187.1048, found 187.1049.

1-(*tert*-Butyl)-3,5-dimethylbenzene-4-*d* (46)[3]

^{1}H NMR (600 MHz, $CDCl_3$) δ 7.01 (s, 2H), 2.32 (s, 6H), 1.30 (s, 9H).

^{13}C NMR (150 MHz, $CDCl_3$) δ 151.3, 137.4, 123.3, 34.6, 31.6, 21.6.

HR-EI-MS (m/z): calcd. for $C_{12}H_{17}D$ [M]: 163.1471, found 163.1473.

Supplementary Note 7. Quantum Yield Measurements

The quantum yield for the model reaction was measured by using Melchiorre's procedure.[4]

A standard ferrioxalate actinometer solution was prepared by following the Hammond variation of the Hatchard and Parker procedure outlined in *Handbook of Photochemistry*.[5] The ferrioxalate actinometer solution measures the decomposition of ferric ions to ferrous ions, which are complexed by 1,10-phenanthroline and monitored by UV/Vis absorbance at 510 nm. The moles of iron-phenanthroline complex $Fe(phen)_3^{2+}$ formed are related to moles of photons absorbed.

The solutions were prepared and stored in dark:

1. Potassium ferrioxalate solution: 589.5 mg of potassium ferrioxalate (commercially available from Alfa Aesar) and 278 μL of sulfuric acid (96%) were added to a 100 mL volumetric flask, and filled to the mark with water (HPLC grade).

2. Phenantroline solution: 0.2% by weight of 1,10-phenanthroline in water (200 mg in 100 mL volumetric flask).

3. Buffer solution: to a 100 mL volumetric flask, 4.94 g of NaOAc and 1 mL of sulfuric acid (96%) were added and filled to the mark with water (HPLC grade).

Procedure: 1 mL of the actinometer solution and 1 mL of the degassed model reaction were added to two identical quartz cuvettes (l = 10 mm). The cuvettes were placed 10 cm away from the light source and irradiated at λ = 390 nm (emission slit width at 10.0 nm) without stirring. This procedure was repeated 3 times, quenching the reactions after different time intervals: 20, 40, 60, and 80 seconds.

The actinometer measurements were done as follows:

1. After irradiation, the actinometer solution was removed and placed in a 10 mL volumetric flask containing 0.5 mL of 1,10-phenanthroline solution and 2 mL of buffer solution. This flask was filled to the mark with water (HPLC grade).

2. The UV-Vis spectra of the complexed actinometer samples were recorded for each time interval. The absorbance of the complexed actinometer solution was

monitored at 510 nm.

The moles of Fe^{2+} formed for each sample are determined according to the Beer's Law:

$$\text{mol Fe}^{2+} = \frac{V_1 \cdot V_3 \cdot \Delta A\,(510\text{ nm})}{10^3 \cdot V_2 \cdot l \cdot \varepsilon\,(510\text{ nm})}$$

where V_1 is the irradiated volume (1 mL), V_2 is the aliquot of the irradiated solution taken for the determination of the ferrous ions (1 mL), V_3 is the final volume after complexation with phenanthroline (10 mL), l is the optical path-length of the irradiation cell (1 cm), ΔA (510 nm) the optical difference in absorbance between the irradiated solution and the one stored in the dark, ε (510 nm) is that of the complex $Fe(phen)_3^{2+}$ (11100 L mol^{-1} cm^{1}).

The moles of Fe^{2+} formed (x) are plotted as a function of time (t). The slope of this line was correlated to the moles of incident photons by unit of time ($q^0_{n,p}$) by the use of the following Equation:[6]

$$\Phi\,(\lambda) = \frac{dx/dt}{q^0_{n,p}[1 - 10^{-A(\lambda)}]}$$

Where dx/dt is the rate of change of a measurable quantity (spectral or any other property), the quantum yield (Φ) for Fe^{2+} at 390 nm is 1.13,[5] and the absorbance A(λ) of the actinometer at λ = 390 nm was measured by UV/Vis spectroscopy to be 0.437. $q^0_{n,p}$, which is the photon flux, was determined to be 4.41 × 10^{-9} einstein s^{-1}.

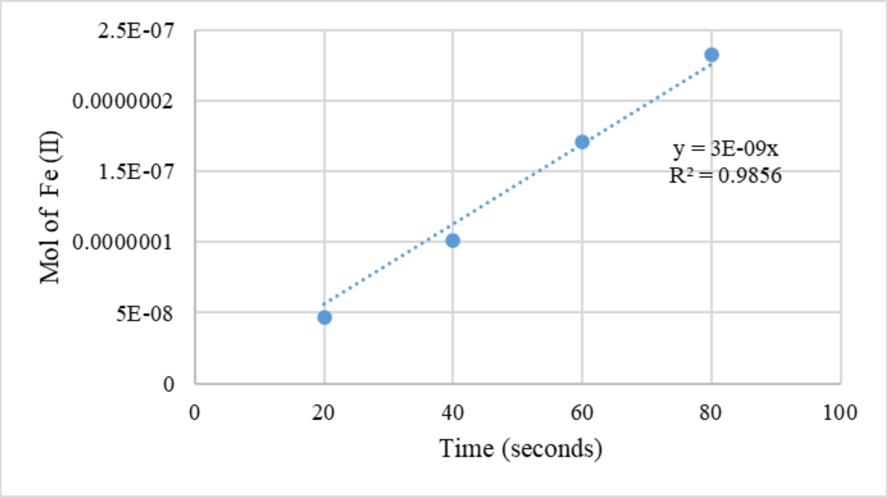

Model reaction solution: To an oven dried quartz cuvettes (l = 10 mm) with a magnetic stirring bar was added thiophenol **1** (11.0 mg, 0.10 mmol), KOH (11.2 mg, 0.20 mmol), 5-(*tert*-butyl)-2-iodo-1,3-dimethylbenzene **2** (57.6 mg, 0.20 mmol) and tetrahydrofuran (1.0 mL) under an argon atmosphere in a glove box. The cuvettes were placed 10 cm away from the light source and irradiated at λ = 390 nm (emission slit width at 10.0 nm). Quench the reaction after different time intervals: 10, 15, 20, and 25 hours.

The measurements for the reaction under study were done as follows: the moles of product **3** formed were determined by ^{1}H NMR spectroscopy. The moles of product per unit of time are related to the number of photons absorbed. The photons absorbed are correlated to the number of incident photons by the use of the equation displayed in the previous point. According to equation the slope (dx/dt) is equal to: $\Phi \cdot (1-10^{-A(390\ nm)}) \cdot q^0_{n,p}$, where Φ is the quantum yield to be determined and the absorption A(390 nm) of the reaction was determined by UV/Vis spectroscopy to be 0.041, thus $(1-10^{-A(390\ nm)})$ = 0.09. The calculation yields the quantum yield (Φ) of the photoreaction = 0.23.

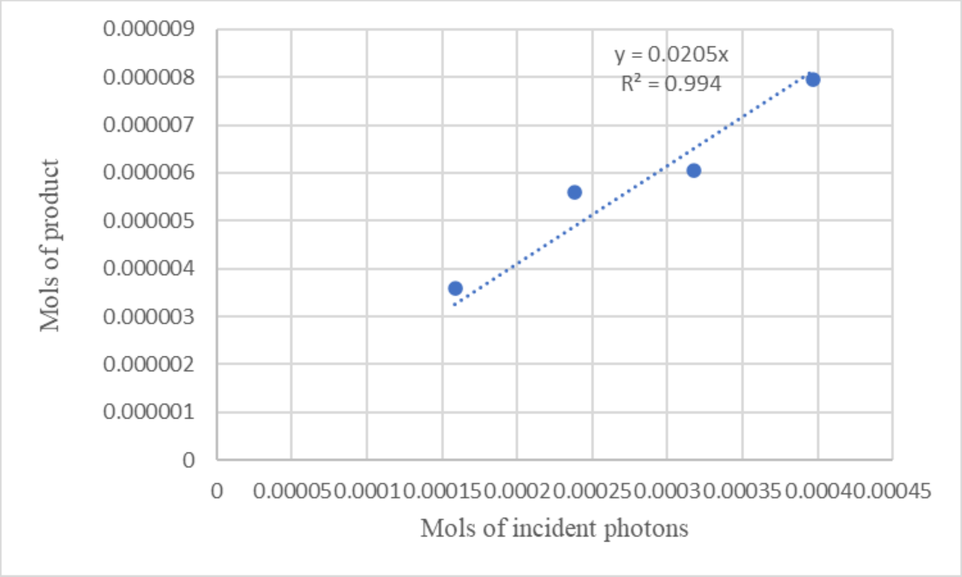

Supplementary Note 8. Density Functional Theory Calculations

DFT calculations were performed with the Gaussian 16 software package, C.01 version.[7] (Unrestricted) B3PW91 was used along with split-valence basis sets[8] 6-31+G(d,p), with diffuse functions[9] and polarization functions[10] added. The effective core potential double-ζ basis set LANL2DZ[11] was used to describe I atom. While optimizations for all other species were carried out in solution-phase with THF as the solvent using the SMD[12] solvation model. Vibrational frequency were also calculated at the same level to confirm that each stationary points found were either minima or transition states and to evaluate thermal corrections at standard states, including zero-point energy corrections. Based on the optimized structure, the single point energies (E_{SPE}) were calculated at M06-2X[13]/def2-TZVP[14] level in gas phase while applying Boys and Bernard's counterpoise (CP) technique[15] to analyze the effect of the basis-set superposition error (BSSE) for the complex at the same level with single point energies used. Binding energies (E_{BE}) is defined as $E_{BE} = E_{SPE}(AB) - E_{SPE}(A) - E_{SPE}(B) + E_{BSSE}$.[16] The electrostatic potential surfaces (ESPs) was mapped by GaussView 6.0.16.[17] The most positive value $V_{S,max}$ and topological analysis were calculated by Multiwfn 3.8 package[18] using the wave function derived from Gaussian. The calculations of distance and angle between σ-hole and Ar-I were the combination of Multiwfn 3.8 package and VMD 1.9.4a53.[19]

Table S1. Single-point energies (SPEs) of Monomers, Counterpoise corrected energy ($E_{corrected}$), BSSE energy (E_{BSSE}) and Binding energy(E_{BE}) for the complex.

	E_{SPEs}[a](a.u.)	$E_{corrected}$[a] (a.u.)	E_{BSSE}[a] (a.u.)	E_{BE}[b] (kcal/mol)
thiophenolate anion(A)	-629.862807	-	-	-
PhI(B)	-765.111805	-	-	-
Adduct(AB)	-1394.989447	-1394.988329	0.001118	**-8.61**

[a]Computed at the M06-2X/def2-TZVP//SMD(THF)/B3PW91/6-31+g(d,p)/LANL2DZ Level. [b]E_{BE} is defined as $E_{BE} = E_{SPEs}(AB) - E_{SPE}(A) - E_{SPE}(B) + E_{BSSE}$ or $E_{corrected}(AB) - E_{SPE}(A) - E_{SPE}(B)$.

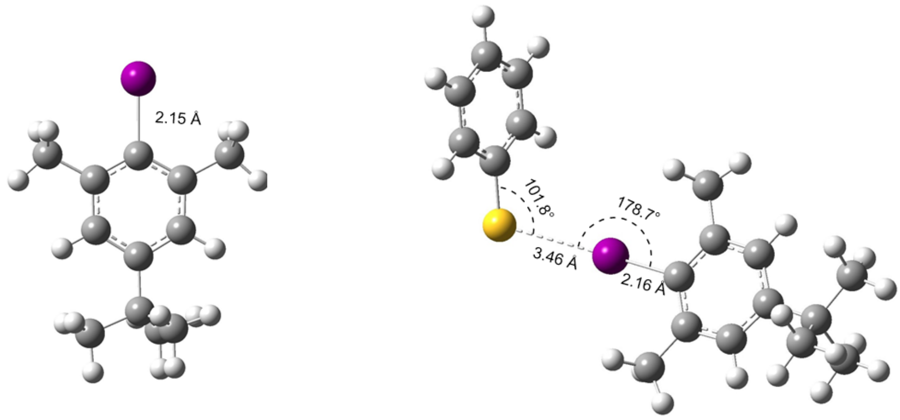

Supplementary Figure 9. The optimized structure of thiophenolate anion and aryl iodide adduct

Table S2. M06-2X/def2-TZVP calculated QTAIM topological parameters at the BCPs of thiophenolate anion and aryl iodide complex.

Interactions	ρ	∇^2 ρ	λ 1	λ 2	λ 3	*V*	*H*	*G*
PhS^-···IAr	0.0134	0.0326	-0.0071	-0.0063	0.0460	-0.0067	0.0007	0.0074

[a] BCPs = bond critical points. ρ = electron density. ∇^2 ρ = Laplacian of electron density. λ = eigenvalues of hessian. *V* = Potential energy density. *H* = Energy density. *G* = Lagrangian kinetic energy. ρ, ∇^2 ρ, *H*, *V*, and *G* are in atomic units. λ is dimensionless.

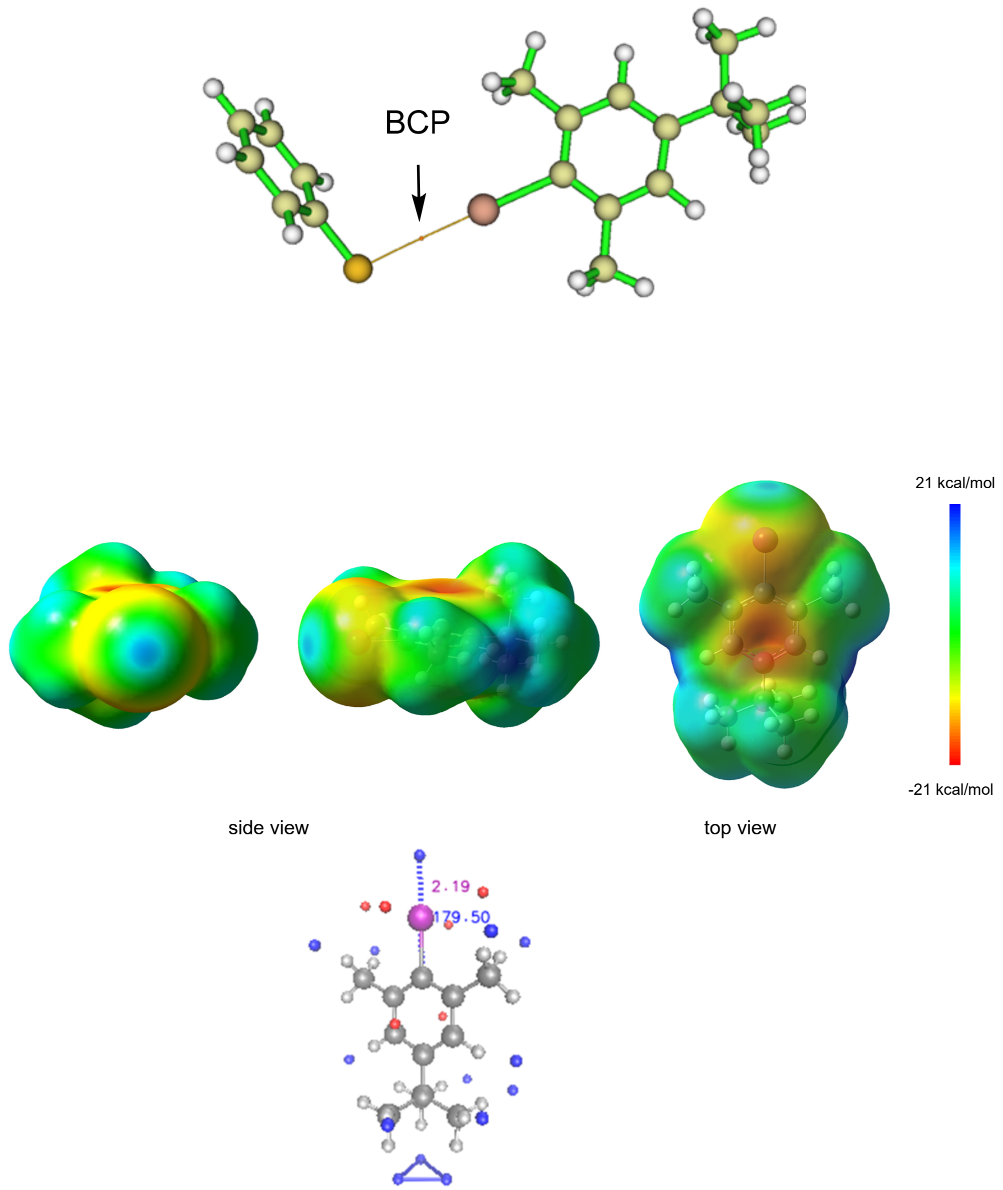

Supplementary Figure 10. Electrostatic potentials mapped on the molecular surface (electron density ρ = 0.001 electrons/bohr^3, kcal/mol) calculated on M06-2X/def2-TZVP level.

Cartesian Coordinates for the Stationary Points

Ar-I 2

0 1

C	-0.02982400	1.24119100	-0.00004200
C	1.37488500	1.21883700	-0.00015900
C	2.11101200	0.03173100	-0.00019500
C	1.38926200	-1.16928100	-0.00009500
C	-0.00994800	-1.21525100	-0.00002600
C	-0.69574500	0.00978000	-0.00000400
C	-0.70578800	-2.54780200	-0.00001900
C	-0.74337900	2.56452000	0.00002500
C	3.64600200	0.00500600	-0.00001500
C	4.25440900	1.41404100	-0.00066200
C	4.14521000	-0.73444400	1.25735400
C	4.14583900	-0.73577400	-1.25633600
H	1.88472100	2.17700800	-0.00025300
H	1.91921900	-2.11870000	-0.00002000
H	0.02383500	-3.36220500	0.00015400
H	-1.34791700	-2.66486300	0.88066700
H	-1.34762000	-2.66499300	-0.88090900
H	-0.02503700	3.38887200	0.00018600
H	-1.38689600	2.67324900	-0.88077300
H	-1.38707500	2.67305400	0.88071200
H	5.34754800	1.33815100	-0.00111800
H	3.96402700	1.98658000	-0.88896700
H	3.96487500	1.98708400	0.88758600
H	5.24160800	-0.75927300	1.27017600
H	3.78868400	-1.76921300	1.29156800
H	3.80740700	-0.23105200	2.17067600
H	3.80804900	-0.23366200	-2.17036800

H	5.24225000	-0.76004900	-1.26882600
H	3.78995800	-1.77079100	-1.28944100
I	-2.84111800	-0.00725500	0.00000000

Thiophenolate anion

-1 1

C	-1.55661200	-1.20148600	0.00001800
C	-0.16223800	-1.20196100	-0.00000600
C	0.58833200	-0.00002000	-0.00009700
C	-0.16222300	1.20194700	-0.00000800
C	-1.55658200	1.20150700	0.00002100
C	-2.27364100	0.00000900	-0.00000800
H	-2.08880000	-2.15141400	0.00004500
H	0.37369600	-2.14851100	0.00001900
H	0.37376200	2.14846900	0.00001600
H	-2.08877800	2.15142900	0.00004700
H	-3.36076000	0.00003500	0.00000800
S	2.34554200	0.00000100	0.00002100

Thiophenolate anion and aryl iodide adduct

-1 1

C	1.37760500	-0.28625800	-0.08693200
C	1.76240100	1.04812800	-0.27564300
C	3.12970000	1.36157900	-0.19399900
C	4.10837900	0.39909600	0.06586000
C	3.67336100	-0.92081500	0.24503300
C	2.32500500	-1.29113700	0.17480100

I	-0.71342400	-0.81263200	-0.19615700
C	5.60388200	0.73411400	0.16232600
C	5.88521300	2.22485800	-0.07085100
C	6.12251300	0.36046300	1.56545200
C	6.38133800	-0.07341200	-0.89617000
C	1.94733400	-2.73297700	0.38052200
C	0.77642700	2.14881300	-0.55899600
S	-4.05458500	-1.71325500	-0.32036800
C	-4.85310700	-0.17913100	-0.00153300
C	-5.05282300	0.78563800	-1.01842600
C	-5.69351400	1.99861700	-0.76577100
C	-6.16555600	2.30609800	0.51487500
C	-5.97921100	1.36924000	1.53728300
C	-5.33742700	0.15656200	1.28577200
H	3.41150400	2.39962300	-0.34145400
H	4.39911700	-1.70476600	0.44891600
H	6.96277500	2.40921500	0.00728600
H	5.56653900	2.55185100	-1.06722200
H	5.38836100	2.85767700	0.67332600
H	7.19100400	0.59416300	1.64976800
H	5.99760600	-0.70710700	1.77510700
H	5.59079500	0.92026700	2.34366800
H	6.03769400	0.17026800	-1.90823500
H	7.45227300	0.15684500	-0.83741200
H	6.26499600	-1.15276500	-0.75264500
H	2.83569600	-3.34121800	0.57451200
H	1.26085400	-2.85148700	1.22688700
H	1.43784600	-3.14401400	-0.49883900
H	0.04023400	2.24655700	0.24725800
H	1.28997000	3.10837800	-0.66955700
H	0.21235100	1.95589300	-1.47897700
H	-4.69501200	0.56655500	-2.02197600

H	-5.82541800	2.71056400	-1.57904100
H	-6.66550100	3.25144900	0.71114100
H	-6.33627800	1.58444000	2.54318300
H	-5.20168300	-0.55765100	2.09491700

Identification of Compounds

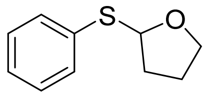

3

2-(Phenylthio)tetrahydrofuran (3)[20]

Prepared according to the general procedure A. The crude mixture was purified by silica gel chromatography (dichloromethane/petroleum ether =1:2) to afford the product **3** as a colorless oil (15.2 mg, 84% yield).

^{1}H NMR (600 MHz, $CDCl_3$) δ 7.55 – 7.48 (m, 2H), 7.30 (t, *J* = 7.7 Hz, 2H), 7.25 – 7.21 (m, 1H), 5.66 (dd, *J* = 7.3, 4.0 Hz, 1H), 4.05 – 4.02 (m, 1H), 3.98 – 3.95 (m, 1H), 2.40 – 2.35 (m, 1H), 2.06 – 1.96 (m, 2H), 1.92 – 1.85 (m, 1H).

^{13}C NMR (150 MHz, $CDCl_3$) δ 135.8, 131.2, 129.0, 126.9, 87.3, 67.4, 32.8, 25.0.

HR-ESI-MS (m/z): calcd. for $C_{10}H_{12}OSNa$ $[M + Na]^+$: 203.0501, found 203.0502.

Scale up reaction for compound (3)

To an oven dried 100 mL glass tube with a magnetic stirring bar was added thiophenol **1** (2.0 mmol), KOH (4.0 mmol), 5-(*t*ert-butyl)-2-iodo-1,3-dimethylbenzene **2** (4.0 mmol) and tetrahydrofuran (20.0 mL) under an argon atmosphere in a glove box. The reaction mixture was stirred under two 7 W 405 nm light emitting diode (LED) lamps (the distance was about 10 cm) irradiation at room temperature. After 36 hours, the reaction was quenched by water (50 mL), extracted with ethyl acetate (50 mL × 3). The combined organic layers were washed with brine, dried over anhydrous Na_2SO_4 and concentrated. The crude product was subjected to column chromatography (dichloromethane/petroleum ether = 1:2) on silica gel to afford 259.0 mg of product **3** in 72% yield.

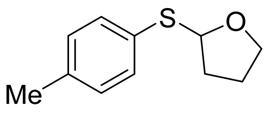

4

2-(*p*-Tolylthio)tetrahydrofuran (4)[20]

Prepared according to the general procedure A. The crude mixture was purified by silica gel chromatography (dichloromethane/petroleum ether =1:2) to afford the product **4** as a colorless oil (15.7 mg, 81% yield).

^{1}H NMR (600 MHz, $CDCl_3$) δ 7.41 (d, J = 8.1 Hz, 2H), 7.11 (d, J = 7.9 Hz, 2H), 5.58 (dd, J = 7.2, 4.0 Hz, 1H), 4.02 (q, J = 7.9 Hz, 1H), 3.95 (td, J = 8.0, 4.0 Hz, 1H), 2.38 – 2.32 (m, 4H), 2.04 – 1.93 (m, 2H), 1.91 – 1.84 (m, 1H).

^{13}C NMR (150 MHz, $CDCl_3$) δ 137.2, 132.0, 131.9, 129.7, 87.7, 67.4, 32.7, 25.0, 21.2.

HR-ESI-MS (m/z): calcd. for $C_{11}H_{14}OSNa$ $[M + Na]^+$: 217.0658, found 203.0655.

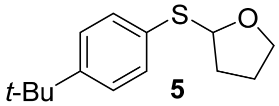

5

2-((4-(*tert*-Butyl)phenyl)thio)tetrahydrofuran (5)[20]

Prepared according to the general procedure A. The crude mixture was purified by silica gel chromatography (dichloromethane/petroleum ether = 1:2) to afford the product **5** as a colorless oil (17.9 mg, 76% yield).

^{1}H NMR (600 MHz, $CDCl_3$) δ 7.46 (d, J = 8.0 Hz, 2H), 7.34 (d, J = 8.0 Hz, 2H), 5.62 (dd, J = 7.4, 4.0 Hz, 1H), 4.05 (q, J = 7.8 Hz, 1H), 3.96 (td, J = 8.1, 4.0 Hz, 1H), 2.40 – 2.32 (m, 1H), 2.06 – 1.95 (m, 2H), 1.88 (m, 1H), 1.32 (s, 9H).

^{13}C NMR (150 MHz, $CDCl_3$) δ 150.2, 132.1, 131.5, 126.0, 87.5, 77.4, 77.2, 76.9, 67.3, 34.6, 32.7, 31.4, 25.0.

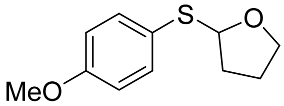

6

2-((4-Methoxyphenyl)thio)tetrahydrofuran (6)[20]

Prepared according to the general procedure A. The crude mixture was purified by silica gel chromatography (dichloromethane/petroleum ether = 1:2) to afford the product **6** as a colorless oil (16.6 mg, 79% yield).

^{1}H NMR (600 MHz, CDCl$_3$) δ 7.46 (d, J = 8.7 Hz, 2H), 6.85 (d, J = 8.7 Hz, 2H), 5.47 (dd, J = 7.2, 3.9 Hz, 1H), 4.01 (q, J = 7.8 Hz, 1H), 3.93 (td, J = 8.0, 4.1 Hz, 1H), 3.79 (s, 3H), 2.34 – 2.28 (m, 1H), 2.02 – 1.91 (m, 2H), 1.89 – 1.82 (m, 1H).

^{13}C NMR (150 MHz, CDCl$_3$) δ 159.5, 134.7, 125.7, 114.5, 88.3, 67.3, 55.4, 32.6, 24.9.

HR-ESI-MS (m/z): calcd. for $C_{11}H_{14}O_2SNa$ [M + Na]$^+$: 233.0607, found 233.0611.

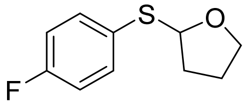

7

2-((4-Fluorophenyl)thio)tetrahydrofuran (7)[20]

Prepared according to the general procedure A. The crude mixture was purified by silica gel chromatography (dichloromethane/petroleum ether = 1:2) to afford the product **7** as a colorless oil (15.4 mg, 78% yield).

^{1}H NMR (600 MHz, CDCl$_3$) δ 7.52 – 7.47 (m, 2H), 7.02 – 6.97 (m, 2H), 5.54 (dd, J = 7.2, 4.0 Hz, 1H), 4.04 – 3.99 (m, 1H), 3.97 – 3.93 (m, 1H), 2.38 – 2.32 (m, 1H), 2.04 – 1.98 (m, 1H), 1.98 – 1.92 (m, 1H), 1.91 – 1.83 (m, 1H).

^{13}C NMR (150 MHz, CDCl$_3$) δ 162.5 (d, J = 246 Hz), 134.1 (d, J = 7.5 Hz), 136.58 (d, J = 4.5 Hz), 116.0 (d, J = 22.5 Hz), 88.0, 67.3, 32.7, 25.0.

HR-ESI-MS (m/z): calcd. for $C_{10}H_{11}FOSNa$ [M + Na]$^+$: 221.0407, found 221.0404.

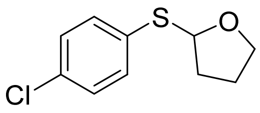

8

2-((4-Chlorophenyl)thio)tetrahydrofuran (8)[20]

Prepared according to the general procedure A. The crude mixture was purified by silica gel chromatography (dichloromethane/petroleum ether = 1:2) to afford the product **8** as a colorless oil (10.9 mg, 51% yield).

^{1}H NMR (600 MHz, $CDCl_3$) δ 7.45 – 7.41 (m, 2H), 7.28 – 7.24 (m, 2H), 5.60 (dd, *J* = 7.3, 4.1 Hz, 1H), 4.01 (q, *J* = 7.9 Hz, 1H), 3.96 (td, *J* = 8.1, 4.2 Hz, 1H), 2.40 – 2.34 (m, 1H), 2.06 – 2.00 (m, 1H), 1.98 – 1.93 (m, 1H), 1.92 – 1.85 (m, 1H).

^{13}C NMR (150 MHz, $CDCl_3$) δ 134.4, 133.1, 132.6, 129.1, 87.4, 67.4, 32.7, 24.9.

HR-ESI-MS (m/z): calcd. for $C_{10}H_{11}ClOSNa$ $[M + Na]^+$: 237.0111, found 237.0109.

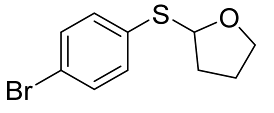

9

2-((4-Bromophenyl)thio)tetrahydrofuran (9)[20]

Prepared according to the general procedure A. The crude mixture was purified by silica gel chromatography (dichloromethane/petroleum ether = 1:2) to afford the product **9** as a colorless oil (16.6 mg, 64% yield).

^{1}H NMR (600 MHz, $CDCl_3$) δ 7.42 – 7.39 (m, 2H), 7.39 – 7.36 (m, 2H), 5.61 (dd, *J* = 7.2, 4.1 Hz, 1H), 4.01 (td, *J* = 8.3, 6.9 Hz, 1H), 3.97 (td, *J* = 8.1, 4.2 Hz, 1H), 2.30 – 2.34 (m, 1H), 2.06 – 1.99 (m, 1H), 1.98 – 1.93 (m, 1H), 1.92 – 1.85 (m, 1H).

^{13}C NMR (150 MHz, $CDCl_3$) δ 135.1, 132.7, 132.0, 121.1, 87.3, 67.5, 32.8, 24.9.

HR-ESI-MS (m/z): calcd. for $C_{10}H_{11}BrOSNa$ $[M + Na]^+$: 280.9606, found 280.9606.

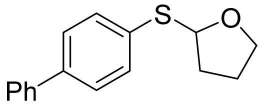

10

2-([1,1'-Biphenyl]-4-ylthio)tetrahydrofuran (10)[20]

Prepared according to the general procedure A. The crude mixture was purified by silica gel chromatography (dichloromethane/petroleum ether = 1:2) to afford the product **10** as a white solid (17.3 mg, 68% yield).

^{1}H NMR (600 MHz, $CDCl_3$) δ 7.62 – 7.56 (m, 4H), 7.53 (d, *J* = 8.4 Hz, 2H), 7.44 (t, *J* = 7.8 Hz, 2H), 7.37 – 7.33 (m, 1H), 5.70 (dd, *J* = 7.3, 4.0 Hz, 1H), 4.06 (q, *J* = 7.9 Hz, 1H), 3.99 (td, *J* = 8.0, 4.0 Hz, 1H), 2.43 – 2.37 (m, 1H), 2.09 – 1.98 (m, 2H), 1.94 – 1.87 (m, 1H).

^{13}C NMR (150 MHz, $CDCl_3$) δ 140.7, 139.9, 134.9, 131.6, 128.9, 127.7, 127.5, 127.1, 87.3, 67.5, 32.8, 25.0.

HR-ESI-MS (m/z): calcd. for $C_{16}H_{16}OSNa$ $[M + Na]^+$: 279.0814, found 279.0811.

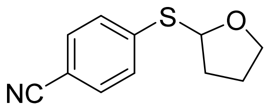

11

4-((Tetrahydrofuran-2-yl)thio)benzonitrile (11)[20]

Prepared according to the general procedure A. Using 4-iodoanisole instead of 5-(*t*ert-butyl)-2-iodo-1,3-dimethylbenzene **2**. The crude mixture was purified by silica gel chromatography (ethyl acetate /petroleum ether = 1:100) to afford the product **11** as pale yellow solid (7.8 mg, 38% yield).

^1H NMR (600 MHz, $CDCl_3$) δ 7.56 – 7.49 (m, 4H), 5.78 (dd, *J* = 7.3, 4.0 Hz, 1H), 4.00 (dd, *J* = 7.9, 5.6 Hz, 2H), 2.46 – 2.40 (m, 1H), 2.09 – 2.03 (m, 1H), 2.02 – 1.96 (m, 1H), 1.95 – 1.88 (m, 1H).

^{13}C NMR (150 MHz, $CDCl_3$) δ 144.2, 132.3, 129.0, 119.0, 109.2, 85.9, 67.7, 32.7, 24.9.

HR-ESI-MS (m/z): calcd. for $C_{11}H_{11}NOSNa$ $[M + Na]^+$: 228.0454, found 228.0459.

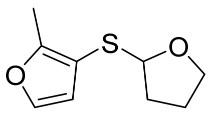

12

2-Methyl-3-((tetrahydrofuran-2-yl)thio)furan (12)

Prepared according to the general procedure A. The crude mixture was purified by silica gel chromatography (dichloromethane/petroleum ether = 1:5) to afford the product **12** as pale yellow oil (7.5 mg, 41% yield).

^1H NMR (600 MHz, $CDCl_3$) δ 7.27 (d, *J* = 1.9 Hz, 1H), 6.41 (d, *J* = 1.9 Hz, 1H), 5.32 (dd, *J* = 7.1, 3.8 Hz, 1H), 3.99 (q, *J* = 7.8 Hz, 1H), 3.90 (td, *J* = 8.0, 4.4 Hz, 1H), 2.35 (s, 3H), 2.29 – 2.23 (m, 1H), 2.03 – 1.96 (m, 1H), 1.96 – 1.90 (m, 1H), 1.89 – 1.81 (m,

1H).

^{13}C NMR (150 MHz, $CDCl_3$) δ 155.5, 140.5, 115.7, 109.5, 87.9, 67.4, 32.5, 24.9, 12.0.

HR-ESI-MS (m/z): calcd. for $C_9H_{12}O_2SNa$ [M + Na]$^+$: 207.0450, found 207.0455.

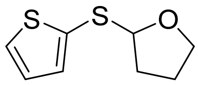

13

2-(Thiophen-2-ylthio)tetrahydrofuran (13)[21]

Prepared according to the general procedure A. The crude mixture was purified by silica gel chromatography (dichloromethane/petroleum ether = 1:2) to afford the product **13** as a colorless oil (8.7 mg, 47% yield).

^{1}H NMR (600 MHz, $CDCl_3$) δ 7.37 (d, *J* = 5.7 Hz, 1H), 7.17 (d, *J* = 3.6 Hz, 1H), 7.00 (dd, *J* = 5.4, 3.5 Hz, 1H), 5.43 (dd, *J* = 7.1, 3.1 Hz, 1H), 4.03 (q, *J* = 8.0 Hz, 1H), 3.95 (td, *J* = 8.2, 4.2 Hz, 1H), 2.33 – 2.25 (m, 1H), 2.02 – 1.95 (m, 2H), 1.91 – 1.84 (m, 1H).

^{13}C NMR (150 MHz, $CDCl_3$) δ 134.4, 132.6, 130.0, 127.6, 89.6, 67.6, 32.3, 24.8.

HR-ESI-MS (m/z): calcd. for $C_8H_{10}OS_2$ [M + Na]$^+$: 209.0065, found 209.0062.

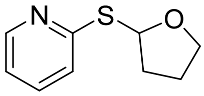

14

2-((Tetrahydrofuran-2-yl)thio)pyridine (14)[21]

Prepared according to the general procedure A. The crude mixture was purified by silica gel chromatography (dichloromethane/petroleum ether = 1:2) to afford the product **14** as a colorless oil (13.0 mg, 72% yield).

^{1}H NMR (600 MHz, $CDCl_3$) δ 8.46 (dd, *J* = 5.0, 2.1 Hz, 1H), 7.51 – 7.48 (m, 1H), 7.30 (d, *J* = 8.0 Hz, 1H), 7.01 – 6.99 (m, 1H), 6.26 – 6.24 (m, 1H), 4.00 – 3.97 (m, 2H), 2.51 – 2.36 (m, 1H), 2.08 – 2.01 (m, 2H), 1.95 – 1.88 (m, 1H).

^{13}C NMR (150 MHz, $CDCl_3$) δ 159.1, 149.8, 136.4, 123.1, 120.1, 84.2, 67.8, 32.3, 25.1.

HR-ESI-MS (m/z): calcd. for $C_9H_{12}NOS$ [M + H]$^+$: 182.0634, found 182.0630.

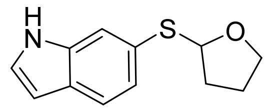

15

6-((Tetrahydrofuran-2-yl)thio)-1*H*-indole (15)

Prepared according to the general procedure A. The crude mixture was purified by silica gel chromatography (dichloromethane/petroleum ether = 1:2) to afford the product **15** as a red oil (10.1 mg, 46% yield).

^{1}H NMR (600 MHz, $CDCl_3$) δ 8.21 (s, 1H), 7.63 (s, 1H), 7.57 (d, *J* = 8.2 Hz, 1H), 7.27 (dd, *J* = 8.2, 1.6 Hz, 1H), 7.17 (t, *J* = 2.8 Hz, 1H), 6.51 (s, 1H), 5.60 (dd, *J* = 7.1, 3.8 Hz, 1H), 4.06 (td, *J* = 8.1, 6.5 Hz, 1H), 3.96 (td, *J* = 8.1, 3.8 Hz, 1H), 2.40 – 2.33 (m, 1H), 2.05 – 1.97 (m, 2H), 1.91 – 1.84 (m, 1H).

^{13}C NMR (150 MHz, $CDCl_3$) δ 136.3, 127.7, 127.6, 124.9, 124.8, 121.0, 115.5, 102.8, 88.5, 67.4, 32.7, 25.1.

HR-ESI-MS (m/z): calcd. for $C_{12}H_{13}NOSNa$ [M + Na]$^+$: 242.0610, found 242.0607.

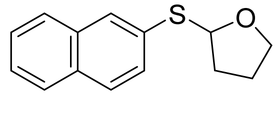

16

2-(Naphthalen-2-ylthio)tetrahydrofuran (16)[20]

Prepared according to the general procedure A. The crude mixture was purified by silica gel chromatography (dichloromethane/petroleum ether = 1:2) to afford the product **16** as a pale yellow oil (13.3 mg, 58% yield).

^{1}H NMR (600 MHz, $CDCl_3$) δ 8.01 (s, 1H), 7.82 – 7.75 (m, 3H), 7.58 (dd, *J* = 8.5, 1.8 Hz, 1H), 7.49 – 7.41 (m, 2H), 5.77 (dd, *J* = 7.1, 3.8 Hz, 1H), 4.08 (td, *J* = 8.1, 6.6 Hz, 1H), 4.01 (td, *J* = 8.2, 3.8 Hz, 1H), 2.46 – 2.39 (m, 1H), 2.10 – 2.01 (m, 2H), 1.96 – 1.87 (m, 1H).

^{13}C NMR (150 MHz, $CDCl_3$) δ 133.9, 133.4, 132.3, 129.5, 128.9, 128.4, 127.8, 127.6,

126.5, 126.0, 87.3, 67.5, 32.9, 25.0.

HR-ESI-MS (m/z): calcd. for $C_{14}H_{14}OSNa$ $[M + Na]^+$: 253.0658, found 253.0655.

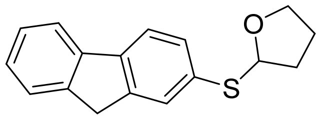

17

2-((9*H*-Fluoren-2-yl)thio)tetrahydrofuran (17)

Prepared according to the general procedure A. The crude mixture was purified by silica gel chromatography (dichloromethane/petroleum ether = 1:2) to afford the product **17** as a yellow solid (11.0 mg, 41% yield).

^1H NMR (600 MHz, $CDCl_3$) δ 7.76 (d, *J* = 7.6 Hz, 1H), 7.72 (s, 1H), 7.71 (d, *J* = 7.9 Hz, 1H), 7.56 – 7.50 (m, 2H), 7.37 (t, *J* = 7.5 Hz, 1H), 7.30 (t, *J* = 7.4 Hz, 1H), 5.69 (dd, *J* = 7.2, 3.9 Hz, 1H), 4.07 (td, *J* = 8.2, 6.8 Hz, 1H), 3.99 (td, *J* = 8.1, 3.8 Hz, 1H), 3.89 (s, 2H), 2.45 – 2.35 (m, 1H), 2.08 – 1.98 (m, 2H), 1.95 – 1.86 (m, 1H).

^{13}C NMR (150 MHz, $CDCl_3$) δ 144.1, 143.3, 141.4, 141.0, 133.8, 130.3, 128.2, 126.9, 125.2, 120.2, 120.0, 87.8, 67.5, 36.9, 32.8, 25.0.

HR-ESI-MS (m/z): calcd. for $C_{17}H_{16}OSNa$ $[M + Na]^+$: 291.0814, found 291.0812.

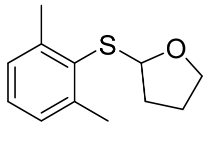

18

2-((2,6-Dimethylphenyl)thio)tetrahydrofuran (18)

Prepared according to the general procedure A. The crude mixture was purified by silica gel chromatography (dichloromethane/petroleum ether = 1:2) to afford the product **18** as a colorless oil (15.2 mg, 73% yield).

^1H NMR (600 MHz, $CDCl_3$) δ 7.16 – 7.08 (m, 3H), 5.38 (dd, *J* = 7.1, 3.6 Hz, 1H), 4.03 (q, *J* = 7.7 Hz, 1H), 3.90 (td, *J* = 8.0, 4.4 Hz, 1H), 2.56 (s, 6H), 2.34 – 2.28 (m, 1H), 2.12 – 1.99 (m, 2H), 1.91 – 1.81 (m, 1H).

^{13}C NMR (150 MHz, $CDCl_3$) δ 143.6, 132.7, 128.6, 128.2, 88.2, 77.4, 33.1, 25.0, 22.5.

HR-ESI-MS (m/z): calcd. for $C_{12}H_{16}OSNa$ [M + Na]$^+$: 231.0814, found 231.0816.

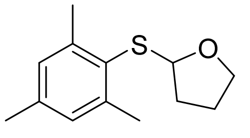

19

2-(Mesitylthio)tetrahydrofuran (19)

Prepared according to the general procedure A. The crude mixture was purified by silica gel chromatography (dichloromethane/petroleum ether = 1:2) to afford the product **19** as a pale yellow oil (17.1 mg, 77% yield).

^{1}H NMR (600 MHz, $CDCl_3$) δ 6.94 (s, 2H), 5.34 (dd, *J* = 7.1, 3.7 Hz, 1H), 4.01 (q, *J* = 7.7 Hz, 1H), 3.88 (td, *J* = 8.1, 4.4 Hz, 1H), 2.51 (s, 6H), 2.32 – 2.27 (m, 1H), 2.26 (s, 3H), 2.10 – 2.03 (m, 1H), 2.02 – 1.97 (m, 1H), 1.88 – 1.81 (m, 1H).

^{13}C NMR (150 MHz, $CDCl_3$) δ 143.4, 138.5, 129.3, 129.1, 88.2, 77.4, 77.2, 76.9, 67.4, 33.0, 25.1, 22.4, 21.1.

HR-ESI-MS (m/z): calcd. for $C_{13}H_{18}OSNa$ [M + Na]$^+$: 245.0971, found 245.0975.

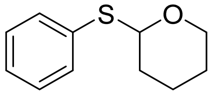

20

2-(Phenylthio)tetrahydro-2*H*-pyran (20)[20]

Prepared according to the general procedure B. The crude mixture was purified by silica gel chromatography (dichloromethane/petroleum ether = 1:2) to afford the product **20** as a pale yellow oil (9.9 mg, 51% yield).

^{1}H NMR (600 MHz, $CDCl_3$) δ 7.48 (d, *J* = 7.4 Hz, 2H), 7.29 (t, *J* = 7.6 Hz, 2H), 7.25 – 7.18 (m, 1H), 5.21 (dd, *J* = 5.9, 3.8 Hz, 1H), 4.20 – 4.16 (m, 1H), 3.64 – 3.55 (m, 1H), 2.07 – 2.00 (m, 1H), 1.90 – 1.81 (m, 2H), 1.68 – 1.59 (m, 3H).

^{13}C NMR (150 MHz, $CDCl_3$) δ 135.6, 131.0, 128.9, 126.9, 85.4, 77.4, 77.2, 76.9, 64.7, 31.7, 25.7, 21.8.

HR-ESI-MS (m/z): calcd. for $C_{11}H_{14}OSNa$ [M + Na]$^+$: 217.0658, found 217.0653.

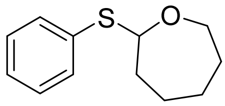

21

2-(Phenylthio)oxepane (21)

Prepared according to the general procedure B. The crude mixture was purified by silica gel chromatography (dichloromethane/petroleum ether = 1:2) to afford the product **21** as a colorless oil (6.9 mg, 33% yield).

^{1}H NMR (600 MHz, $CDCl_3$) δ 7.49 (d, *J* = 7.8 Hz, 2H), 7.28 (t, *J* = 7.5 Hz, 2H), 7.24 – 7.20 (m, 1H), 5.34 (dd, *J* = 11.1, 5.5 Hz, 1H), 3.96 – 3.92 (m, 1H), 3.66 – 3.62 (m, 1H), 2.37 – 2.31 (m, 1H), 1.90 – 1.85 (m, 1H), 1.81 – 1.73 (m, 3H), 1.69 – 1.61 (m, 1H), 1.58 – 1.52 (m, 1H), 1.37 – 1.29 (m, 1H).

^{13}C NMR (150 MHz, $CDCl_3$) δ 135.9, 131.3, 128.9, 126.9, 87.9, 63.4, 36.1, 30.5, 29.3, 25.3.

HR-ESI-MS (m/z): calcd. for $C_{12}H_{16}OSNa$ $[M + Na]^+$: 231.0814, found 231.0817.

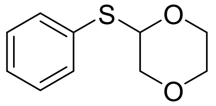

22

2-(Phenylthio)-1,4-dioxane (22)[20]

Prepared according to the general procedure B. The crude mixture was purified by silica gel chromatography (dichloromethane/petroleum ether = 1:2) to afford the product **22** as a colorless oil (10.2 mg, 52% yield).

^{1}H NMR (600 MHz, $CDCl_3$) δ 7.51 (d, *J* = 7.0 Hz, 2H), 7.34 – 7.30 (m, 2H), 7.29 – 7.27 (m, 1H), 5.13 (dd, *J* = 5.7, 2.9 Hz, 1H), 4.24 (dt, *J* = 11.6, 4.4 Hz, 1H), 4.00 (dd, *J* = 11.8, 3.0 Hz, 1H), 3.75 – 3.67 (m, 4H).

^{13}C NMR (150 MHz, $CDCl_3$) δ 134.1, 131.7, 129.1, 127.5, 83.4, 70.1, 66.6, 64.0.

HR-ESI-MS (m/z): calcd. for $C_{10}H_{12}O_2SNa$ $[M + Na]^+$: 219.0450, found 219.0447.

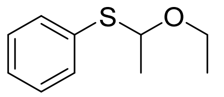

23

(1-Ethoxyethyl)(phenyl)sulfane (23)[20]

Prepared according to the general procedure B. The crude mixture was purified by silica gel chromatography (dichloromethane/petroleum ether = 1:100) to afford the product **23** as a colorless oil (11.3 mg, 62% yield).

^{1}H NMR (600 MHz, $CDCl_3$) δ 7.50 – 7.46 (m, 2H), 7.33 – 7.27 (m, 3H), 4.90 (q, J = 6.3 Hz, 1H), 3.98 – 3.93 (m, 1H), 3.53 – 3.48 (m, 1H), 1.51 (d, J = 6.3 Hz, 3H), 1.23 (t, J = 7.0 Hz, 3H).

^{13}C NMR (150 MHz, $CDCl_3$) δ 133.9, 133.2, 128.8, 127.7, 127.6, 84.7, 63.5, 22.8, 15.1.

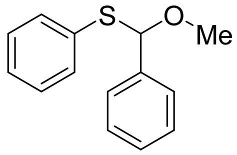

24

(Methoxy(phenyl)methyl)(phenyl)sulfane (24)[22]

Prepared according to the general procedure B. The crude mixture was purified by silica gel chromatography (dichloromethane/petroleum ether = 1:2) to afford the product **24** as a colorless oil (17.3 mg, 75% yield).

^{1}H NMR (600 MHz, $CDCl_3$) δ 7.31 – 7.29 (m, 2H), 7.26 – 7.23 (m, 3H), 7.22 – 7.18 (m, 5H), 5.69 (s, 1H), 3.50 (s, 3H).

^{13}C NMR (150 MHz, $CDCl_3$) δ 139.4, 133.9, 133.1, 128.7, 128.2, 128.0, 127.9, 126.4, 91.3, 56.6.

HR-ESI-MS (m/z): calcd. for $C_{14}H_{14}OSNa$ $[M + Na]^{+}$: 253.0658, found 253.0654.

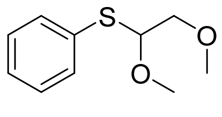

25

(1,2-Dimethoxyethyl)(phenyl)sulfane (25)[20]

Prepared according to the general procedure B. The crude mixture was purified by silica gel chromatography (dichloromethane/petroleum ether = 1:2) to afford the product **25** as a colorless oil (6.1 mg, 31% yield).

^{1}H NMR (600 MHz, $CDCl_3$) δ 7.52 – 7.47 (m, 2H), 7.34 – 7.28 (m, 3H), 4.75 (dd, J = 7.9, 4.0 Hz, 1H), 3.62 – 3.59 (m, 1H), 3.57 (s, 3H), 3.53 – 3.50 (m, 1H), 3.36 (s, 3H).

^{13}C NMR (150 MHz, $CDCl_3$) δ 134.1, 132.5, 129.0, 128.0, 89.1, 74.9, 59.2, 56.5.

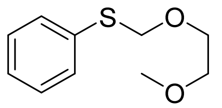

26

((2-Methoxyethoxy)methyl)(phenyl)sulfane (26)[20]

Prepared according to the general procedure B. The crude mixture was purified by silica gel chromatography (dichloromethane/petroleum ether = 1:2) to afford the product **26** as a colorless oil (5.2 mg, 26% yield).

^{1}H NMR (600 MHz, $CDCl_3$) δ 7.50 – 7.47 (m, 2H), 7.30 – 7.27 (m, 2H), 7.24 – 7.20 (m, 1H), 5.07 (s, 2H), 3.80 – 3.77 (m, 2H), 3.59 – 3.57 (m, 2H), 3.39 (s, 3H).

^{13}C NMR (150 MHz, $CDCl_3$) δ 136.1, 130.3, 129.1, 126.8, 76.6, 71.7, 67.6, 59.2.

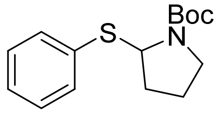

27

***tert*-Butyl 2-(phenylthio)pyrrolidine-1-carboxylate (27)**[23]

Prepared according to the general procedure B. The crude mixture was purified by silica gel chromatography (ethyl acetate/petroleum ether = 1:50) to afford the product **27** as a colorless oil (14.5 mg, 52% yield).

^{1}H NMR (600 MHz, $CDCl_3$) δ 7.60 – 7.40 (m, 2H), 7.35 – 7.25 (m, 3H), 5.49 – 5.15 (m, 1H), 3.49 – 3.25 (m, 2H), 2.20 – 1.86 (m, 4H), 1.46 – 1.30 (m, 9H).

^{13}C NMR (150 MHz, $CDCl_3$) δ 153.7, 134.7, 134.4, 134.2, 129.0, 128.1, 127.8, 80.2, 79.8, 77.4, 77.2, 76.9, 67.2, 66.9, 46.4, 45.7, 34.1, 33.4, 28.5, 28.3, 23.1, 22.3.

HR-ESI-MS (m/z): calcd. for $C_{15}H_{21}NO_2SNa$ $[M + Na]^+$: 302.1185, found 302.1181.

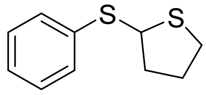

28

2-(Phenylthio)tetrahydrothiophene (28)[24]

Prepared according to the general procedure B. The crude mixture was purified by silica gel chromatography (dichloromethane/petroleum ether = 1:2) to afford the product **28** as a white solid (14.9 mg, 76% yield).

^{1}H NMR (600 MHz, $CDCl_3$) δ 7.45 – 7.39 (m, 2H), 7.34 – 7.28 (m, 2H), 7.26 – 7.23 (m, 1H), 4.86 (t, *J* = 4.6 Hz, 1H), 3.10 – 3.01 (m, 1H), 2.92 – 2.82 (m, 1H), 2.27 – 2.19 (m, 1H), 2.19 – 2.14 (m, 2H), 2.13 – 2.06 (m, 1H).

^{13}C NMR (150 MHz, $CDCl_3$) δ 136.4, 131.5, 129.1, 127.3, 55.8, 38.5, 33.3, 28.9.

HR-ESI-MS (m/z): calcd. for $C_{10}H_{12}S_2Na$ $[M + Na]^+$: 219.0273, found 219.0276.

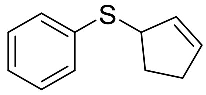

29

Cyclopent-2-en-1-yl(phenyl)sulfane (29)[25]

Prepared according to the general procedure B. The crude mixture was purified by silica gel chromatography (dichloromethane/petroleum ether = 1:200) to afford the product **29** as a pale yellow oil (11.6 mg, 66% yield).

^{1}H NMR (600 MHz, $CDCl_3$) δ 7.42 – 7.38 (m, 2H), 7.31 – 7.27 (m, 2H), 7.24 – 7.18 (m, 1H), 5.96 – 5.86 (dq, *J* = 5.8, 2.0 Hz, 1H), 5.86 – 5.77 (m, 1H), 4.38 – 4.25 (ddd, *J* = 8.7, 3.2, 1.6 Hz, 1H), 2.48 – 2.28 (m, 3H), 2.06 – 1.97 (m, 1H).

^{13}C NMR (150 MHz, $CDCl_3$) δ 136.4, 134.0, 131.2, 131.1, 128.9, 126.5, 52.8, 31.7, 31.5.

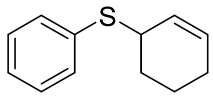

30

Cyclohex-2-en-1-yl(phenyl)sulfane (30)[25]

Prepared according to the general procedure B. The crude mixture was purified by silica gel chromatography (dichloromethane/petroleum ether = 1:200) to afford the product **30** as a colorless oil (13.8 mg, 73% yield).

^{1}H NMR (600 MHz, $CDCl_3$) δ 7.45 – 7.40 (m, 2H), 7.31 – 7.28 (m, 2H), 7.25 – 7.17 (m, 1H), 5.88 – 5.81 (m, 1H), 5.81 – 5.74 (m, 1H), 3.90 – 3.83 (m, 1H), 2.08 – 2.00 (m, 2H), 1.99 – 1.87 (m, 2H), 1.83 – 1.74 (m, 1H), 1.66 – 1.58 (m, 1H).

^{13}C NMR (150 MHz, $CDCl_3$) δ 136.0, 131.4, 130.5, 129.0, 127.1, 126.7, 44.0, 29.0, 25.1, 19.6.

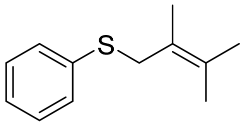

31

(2,3-Dimethylbut-2-en-1-yl)(phenyl)sulfane (31)[25]

Prepared according to the general procedure B. The crude mixture was purified by silica gel chromatography (dichloromethane/petroleum ether = 1:200) to afford the product **31** as a colorless oil (13.2 mg, 69% yield).

^{1}H NMR (600 MHz, $CDCl_3$) δ 7.35 – 7.30 (m, 2H), 7.26 – 7.22 (m, 2H), 7.20 – 7.13 (m, 1H), 3.57 (s, 2H), 1.78 (s, 3H), 1.65 (s, 3H), 1.55 (s, 3H).

^{13}C NMR (150 MHz, $CDCl_3$) δ 137.4, 130.5, 129.9, 128.7, 126.3, 122.9, 39.4, 21.0, 20.3, 18.3.

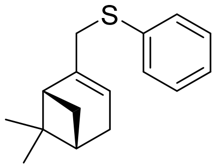

32

(((1*R*,5*S*)-6,6-Dimethylbicyclo[3.1.1]hept-2-en-2-yl)methyl)(phenyl)sulfane (32)

Prepared according to the general procedure B. The crude mixture was purified by silica gel chromatography (dichloromethane/petroleum ether = 1:200) to afford the product **32** as a colorless oil (11.7 mg, 48% yield). $[\alpha]_D^{23}$ = − 10.4 (c = 0.36, $CHCl_3$).

^{1}H NMR (600 MHz, $CDCl_3$) δ 7.33 – 7.29 (m, 2H), 7.26 – 7.24 (m, 2H), 7.17 – 7.13 (m, 1H), 5.44 – 5.41 (m, 1H), 3.57 – 3.49 (m, 2H), 2.39 – 2.36 (m, 1H), 2.27 – 2.15 (m, 3H), 2.07 – 2.04 (m, 1H), 1.28 (s, 3H), 1.08 (d, *J* = 8.6 Hz, 1H), 0.77 (s, 3H).

^{13}C NMR (150 MHz, $CDCl_3$) δ 143.1, 137.1, 129.4, 128.8, 125.8, 120.7, 45.4, 40.7, 40.6, 38.3, 31.8, 31.4, 26.3, 21.3.

[illegible]R-EI-MS (m/z): calcd. for $C_{16}H_{20}S$ [M]: 244.1286, found 244.1283.

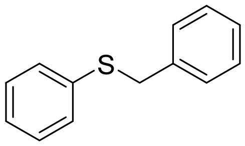

33

Benzyl(phenyl)sulfane (33)[26]

Prepared according to the general procedure B. The crude mixture was purified by silica gel chromatography (dichloromethane/petroleum ether = 1:200) to afford the product **33** as a yellow solid (14.0 mg, 70% yield).

^{1}H NMR (600 MHz, $CDCl_3$) δ 7.35 – 7.24 (m, 9H), 7.23 – 7.18 (m, 1H), 4.15 (s, 2H).

^{13}C NMR (150 MHz, $CDCl_3$) δ 137.6, 136.5, 130.0, 129.0, 128.6, 127.3, 126.5, 39.2.

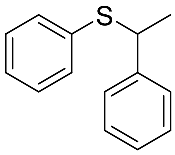

34

Phenyl(1-phenylethyl)sulfane (34)[26]

Prepared according to the general procedure B. The crude mixture was purified by silica gel chromatography (dichloromethane/petroleum ether = 1:200) to afford the product **34** as a colorless oil (12.6 mg, 59% yield).

^{1}H NMR (600 MHz, $CDCl_3$) δ 7.35 – 7.26 (m, 6H), 7.25 – 7.18 (m, 4H), 4.36 (q, *J* = 7.0 Hz, 1H), 1.64 (d, *J* = 7.0 Hz, 3H).

^{13}C NMR (150 MHz, $CDCl_3$) δ 143.4, 135.3, 132.6, 128.8, 128.5, 127.4, 127.3, 127.2, 48.1, 22.5.

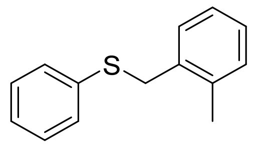

35

(2-Methylbenzyl)(phenyl)sulfane (35)[27]

Prepared according to the general procedure B. The crude mixture was purified by silica gel chromatography (dichloromethane/petroleum ether = 1:200) to afford the product **35** as a colorless oil (15.2 mg, 71% yield).

^{1}H NMR (600 MHz, $CDCl_3$) δ 7.36 – 7.32 (m, 2H), 7.31 – 7.27 (m, 2H), 7.24 – 7.20 (m, 1H), 7.20 – 7.14 (m, 3H), 7.14 – 7.08 (m, 1H), 4.13 (s, 2H), 2.42 (s, 3H).

^{13}C NMR (150 MHz, $CDCl_3$) δ 136.9, 136.8, 135.2, 130.6, 130.4, 129.9, 129.0, 127.6, 126.6, 126.1, 37.6, 19.3.

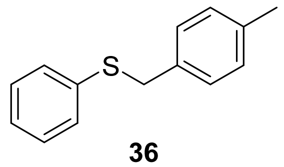

36

(4-Methylbenzyl)(phenyl)sulfane (36)[26]

Prepared according to the general procedure B. The crude mixture was purified by silica gel chromatography (dichloromethane/petroleum ether = 1:200) to afford the product **36** as a colorless oil (14.4 mg, 67% yield).

^{1}H NMR (600 MHz, $CDCl_3$) δ 7.31 (d, J = 7.6 Hz, 2H), 7.25 (m, 2H), 7.21 – 7.15 (m, 3H), 7.09 (d, J = 7.7 Hz, 2H), 4.10 (s, 2H), 2.32 (s, 3H).

^{13}C NMR (150 MHz, $CDCl_3$) δ 137.0, 136.8, 134.5, 129.8, 129.3, 129.0, 128.9, 126.4, 77.4, 77.2, 76.9, 38.9, 21.2.

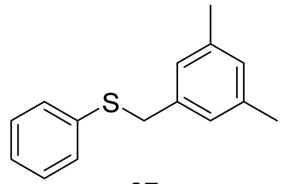

37

(3,5-Dimethylbenzyl)(phenyl)sulfane (37)[26]

Prepared according to the general procedure B. The crude mixture was purified by silica gel chromatography (dichloromethane/petroleum ether = 1:200) to afford the product **37** as a yellow solid (17.6 mg, 77% yield).

^{1}H NMR (600 MHz, $CDCl_3$) δ 7.36 – 7.32 (m, 2H), 7.30 – 7.27 (m, 2H), 7.22 – 7.18 (m, 1H), 6.95 (s, 2H), 6.90 (s, 1H), 4.08 (s, 2H), 2.30 (s, 6H).

^{13}C NMR (150 MHz, $CDCl_3$) δ 138.2, 137.2, 137.0, 129.6, 129.0, 128.9, 126.8, 126.3, 39.1, 21.3.

HR-ESI-MS (m/z): calcd. for $C_{15}H_{16}SNa$ $[M + Na]^+$: 251.0865, found 251.0862.

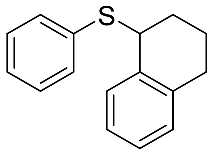

38

Phenyl(1,2,3,4-tetrahydronaphthalen-1-yl)sulfane (38)[28]

Prepared according to the general procedure B. The crude mixture was purified by silica gel chromatography (dichloromethane/petroleum ether = 1:200) to afford the product **38** as a colorless oil (12.7 mg, 53% yield).

^{1}H NMR (600 MHz, $CDCl_3$) δ 7.51 – 7.47 (m, 2H), 7.45 – 7.42 (m, 1H), 7.36 – 7.33 (m, 2H), 7.30 – 7.26 (m, 1H), 7.20 –7.15(m, 2H), 7.14 – 7.10 (m, 1H), 4.60 (t, *J* = 4.1 Hz, 1H), 2.89 – 2.85 (m, 1H), 2.80 – 2.75 (m, 1H), 2.29 – 2.22 (m, 1H), 2.09 – 2.05 (m, 1H), 2.03 – 1.97 (m, 1H), 1.81 – 1.76 (m, 1H).

^{13}C NMR (150 MHz, $CDCl_3$) δ 137.7, 136.3, 135.6, 132.0, 132.0, 130.7, 130.6, 129.4, 129.1, 127.2, 127.1, 125.8, 47.8, 29.2, 28.7, 18.8.

HR-ESI-MS (m/z): calcd. for $C_{16}H_{16}SNa$ $[M + Na]^+$: 263.0865, found 251.0869.

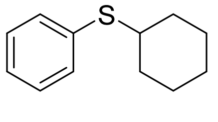

39

Cyclohexyl(phenyl)sulfane (39)[29]

Prepared according to the general procedure B. The crude mixture was purified by

silica gel chromatography (dichloromethane/petroleum ether = 1:200) to afford the product **39** as a colorless oil (4.8 mg, 25% yield).

^{1}H NMR (600 MHz, $CDCl_3$) δ 7.40 (d, *J* = 6.9 Hz, 2H), 7.32 – 7.24 (m, 2H), 7.24 – 7.16 (m, 1H), 3.13 – 3.08(m, 1H), 2.03 – 1.94 (m, 2H), 1.79 – 1.75 (m, 2H), 1.63 – 1.59 (m, 1H), 1.43 – 1.20 (m, 6H).

^{13}C NMR (150 MHz, $CDCl_3$) δ 135.4, 132.0, 128.9, 126.7, 46.7, 33.5, 26.2, 25.9.

2-(Benzylthio)-1-methyl-1*H*-benzo[*d*]imidazole (41)[30]

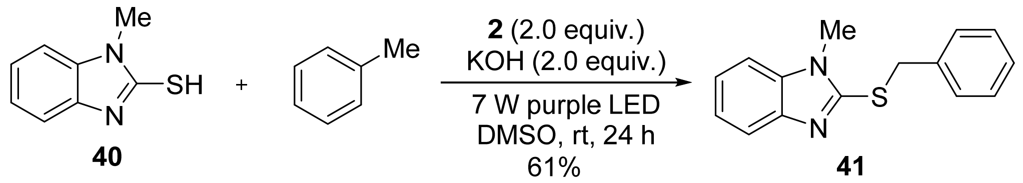

To an oven dried 10 mL glass tube with a magnetic stirring bar was added 1-methyl-1*H*-benzo[*d*]imidazole-2-thiol **40** (16.4 mg, 0.10 mmol), toluene (372 μL, 3.00 mmol), KOH (11.2 mg, 0.20 mmol), 5-(*t*ert-butyl)-2-iodo-1,3-dimethylbenzene **2** (57.6 mg, 0.20 mmol) and DMSO (1.0 mL) under an argon atmosphere in a glove box. The reaction mixture was stirred under two 7 W 405 nm light emitting diode (LED) lamps (the distance was about 10 cm) irradiation at room temperature. After 24 hours, the reaction was quenched by water (15 mL), extracted with ethyl acetate (15 mL × 3). The combined organic layers were washed with brine, dried over anhydrous Na_2SO_4 and concentrated. The crude product was subjected to column chromatography (ethyl acetate/petroleum ether = 1:10) on silica gel to afford 15.6 mg of product **41** in 61% yield as a colorless oil. ^{1}H NMR (600 MHz, $CDCl_3$) δ 7.73 – 7.68 (m, 1H), 7.41 – 7.36 (m, 2H), 7.29 (t, *J* = 7.7 Hz, 2H), 7.25 – 7.20 (m, 4H), 4.59 (s, 2H), 3.57 (s, 3H).
^{13}C NMR (150 MHz, $CDCl_3$) δ 151.7, 143.5, 136.9, 136.8, 129.2, 128.8, 127.8, 122.1, 122.0, 118.5, 108.7, 37.4, 30.1.

References

1. Ye, Z.-P.; Xia, P.-J.; Liu, F.; Hu, Y.-Z.; Song, D.; Xiao, J.-A.; Huang, P.; Xiang, H.-Y.; Chen, X.-Q.; Yang, H., Visible-Light-Induced, Catalyst-Free Radical Cross-Coupling Cyclization of N-Allylbromodifluoroacetamides with Disulfides or Diselenides. *J. Org. Chem.* **2020,** *85*, 5670.
2. Liu, D.; Liu, C.; Li, H.; Lei, A., Copper-catalysed oxidative C–H/C–H coupling between olefins and simple ethers. *Chem. Commun.* **2014,** *50*, 3623.
3. Liu, Z.; Li, M.; Deng, G.; Wei, W.; Feng, P.; Zi, Q.; Li, T.; Zhang, H.; Yang, X.; Walsh, P. J., Transition-metal-free C(sp^3)–H/C(sp^3)–H dehydrogenative coupling of saturated heterocycles with N-benzyl imines. *Chem. Sci.* **2020,** *11*, 7619.
4. Bahamonde, A.; Melchiorre, P., Mechanism of the Stereoselective α-Alkylation of Aldehydes Driven by the Photochemical Activity of Enamines. *J. Am. Chem. Soc.* **2016,** *138*, 8019.
5. Murov, S. L., *Handbook of photochemistry*. Dekker, New York ed.1973.
6. (a) Braslavsky, S. E., Glossary of terms used in photochemistry, 3rd edition (IUPAC Recommendations 2006). *Pure Appl. Chem.* **2007,** *79*, 293; (b) Sun, L.; Bolton, J. R., Determination of the Quantum Yield for the Photochemical Generation of Hydroxyl Radicals in TiO2 Suspensions. *J. Phys. Chem.* **1996,** *100*, 4127.
7. Gaussian 16, Revision C.01, M. J. Frisch, G. W. Trucks, H. B. Schlegel, G. E. Scuseria, M. A. Robb, J. R. Cheeseman, G. Scalmani, V. Barone, G. A. Petersson, H. Nakatsuji, X. Li, M. Caricato, et al., Gaussian, Inc., Wallingford CT, 2019.
8. Hehre, W. J.; Ditchfield, R.; Pople, J. A., Self—Consistent Molecular Orbital Methods. XII. Further Extensions of Gaussian—Type Basis Sets for Use in Molecular Orbital Studies of Organic Molecules. *J. Chem. Phys.* **1972,** *56*, 2257.
9. Clark, T.; Chandrasekhar, J.; Spitznagel, G. W.; Schleyer, P. V. R., Efficient diffuse function-augmented basis sets for anion calculations. III. The 3-21+G basis set for first-row elements, Li–F. *J. Comput. Chem.* **1983,** *4*, 294.
10. Hariharan, P. C.; Pople, J. A., The influence of polarization functions on molecular orbital hydrogenation energies. *Theor. Chim. Acta* **1973,** *28*, 213.
11. Hay, P. J.; Wadt, W. R., Ab initio effective core potentials for molecular calculations. Potentials for K to Au including the outermost core orbitals. *J. Chem. Phys.* **1985,** *82*, 299.
12. Marenich, A. V.; Cramer, C. J.; Truhlar, D. G., Universal Solvation Model Based on Solute Electron Density and on a Continuum Model of the Solvent Defined by the Bulk Dielectric Constant and Atomic Surface Tensions. *J. Phys. Chem. B.* **2009,** *113*, 6378.
13. Hohenstein, E. G.; Chill, S. T.; Sherrill, C. D., Assessment of the Performance of the M05−2X and M06−2X Exchange-Correlation Functionals for Noncovalent Interactions in Biomolecules. *J. Chem. Theory Comput.* **2008,** *4*, 1996.
14. Weigend, F.; Ahlrichs, R., Balanced basis sets of split valence, triple zeta valence and quadruple zeta valence quality for H to Rn: Design and assessment of accuracy. *Phys. Chem. Chem. Phys.* **2005,** *7*, 3297.
15. Boys, S. F.; Bernardi, F., The calculation of small molecular interactions by the differences of separate total energies. Some procedures with reduced errors. *Mol. Phys.* **1970,** *19*, 553.
16. Wang, G.; Chen, Z.; Xu, Z.; Wang, J.; Yang, Y.; Cai, T.; Shi, J.; Zhu, W., Stability and Characteristics of the Halogen Bonding Interaction in an Anion–Anion Complex: A Computational

Chemistry Study. *J. Phys. Chem. B* **2016,** *120*, 610.
17. GaussView, Version 6.1.1, Roy Dennington, Todd Keith, and John Millam, Semichem Inc., Shawnee Mission, KS, 2019.
18. Lu, T.; Chen, F., Multiwfn: A multifunctional wavefunction analyzer. *J. Comput. Chem.* **2012,** *33*, 580.
19. Cosgrove, D. A.; Kenny, P. W., BOOMSLANG: A program for combinatorial structure generation. *J. Mol. Graphics* **1996,** *14*, 1.
20. Zhu, X.; Xie, X.; Li, P.; Guo, J.; Wang, L., Visible-Light-Induced Direct Thiolation at α-C(sp^3)–H of Ethers with Disulfides Using Acridine Red as Photocatalyst. *Org. Lett.* **2016,** *18*, 1546.
21. Dong, Y.; Ji, P.; Zhang, Y.; Wang, C.; Meng, X.; Wang, W., Organophotoredox-Catalyzed Formation of Alkyl–Aryl and –Alkyl C–S/Se Bonds from Coupling of Redox-Active Esters with Thio/Selenosulfonates. *Org. Lett.* **2020,** *22*, 9562.
22. Sato, T.; Otera, J.; Nozaki, H., Activation and synthetic applications of thiostannanes: Thioalkoxylation of acetals. *Tetrahedron* **1989,** *45*, 1209.
23. Jin, Y.; Yang, H.; Fu, H., An N-(acetoxy)phthalimide motif as a visible-light pro-photosensitizer in photoredox decarboxylative arylthiation. *Chem. Commun.* **2016,** *52*, 12909.
24. Kazuhiro, K.; Masataka, K.; Hideki, A.; Osamu, M.; Hisatoshi, K., An Efficient Synthesis of Unsymmetrical Dithioacetals from Sulfoxides and Thiols by the Magnesium Amide-Induced Pummerer-Type Reaction. *Bull. Chem. Soc. Jpn.* **1996,** *69*, 2645.
25. Kim, J.; Kang, B.; Hong, S. H., Direct Allylic C(sp^3)–H Thiolation with Disulfides via Visible Light Photoredox Catalysis. *ACS Catal.* **2020,** *10*, 6013.
26. Sorribes, I.; Corma, A., Nanolayered cobalt–molybdenum sulphides (Co–Mo–S) catalyse borrowing hydrogen C–S bond formation reactions of thiols or H_2S with alcohols. *Chem. Sci.* **2019,** *10*, 3130.
27. Villuendas, P.; Urriolabeitia, E. P., Ru-Catalyzed Regioselective CH-Hydroarylation of Alkynes with Benzylthioethers Using Sulfur as Directing Group. *Org. Lett.* **2015,** *17*, 3178.
28. Li, F.; Wang, D.; Chen, H.; He, Z.; Zhou, L.; Zeng, Q., Transition metal-free coupling reactions of benzylic trimethylammonium salts with di(hetero)aryl disulfides and diselenides. *Chem. Commun.* **2020,** *56*, 13029.
29. Du, B.; Jin, B.; Sun, P., Syntheses of Sulfides and Selenides through Direct Oxidative Functionalization of C(sp^3)–H Bond. *Org. Lett.* **2014,** *16*, 3032.
30. Rao, S. S.; Reddy, C. V. R.; Dubey, P. K., A green approach for the synthesis of 1-methyl-2-(alkylthio)-1H-benzimidazoles. *Asian J. Chem.* **2014,** *26*, 5995.

Copies of NMR Spectra

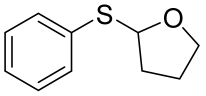

(**3**, 600 MHz, $CDCl_3$)

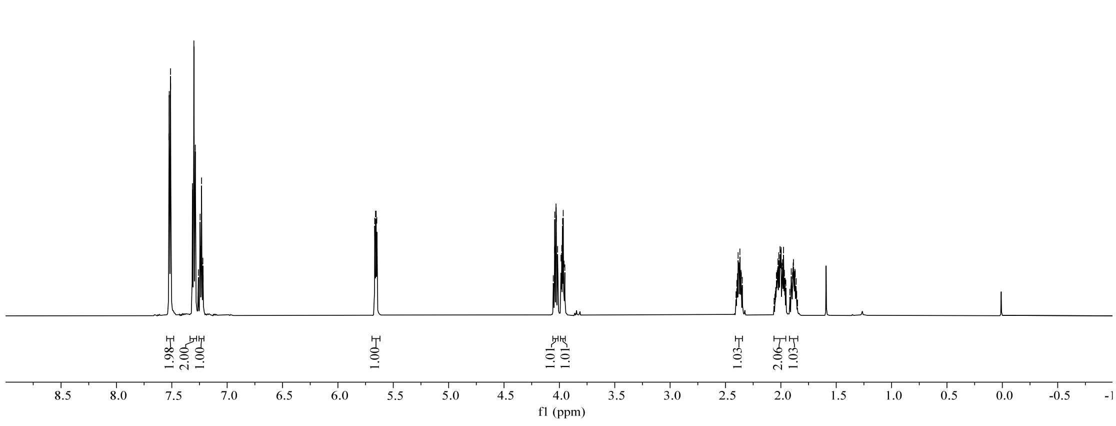

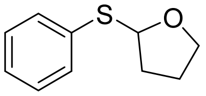

(**3**, 150 MHz, $CDCl_3$)

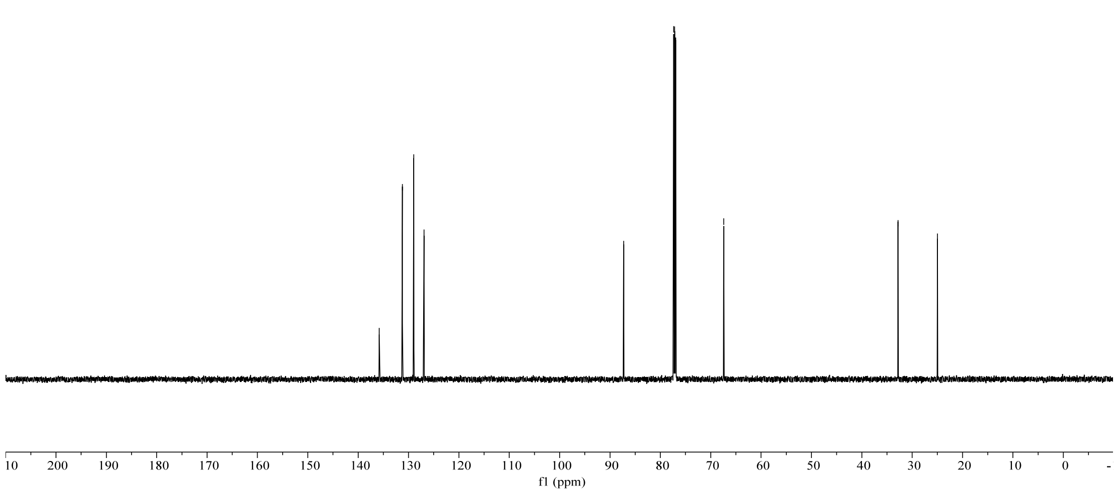

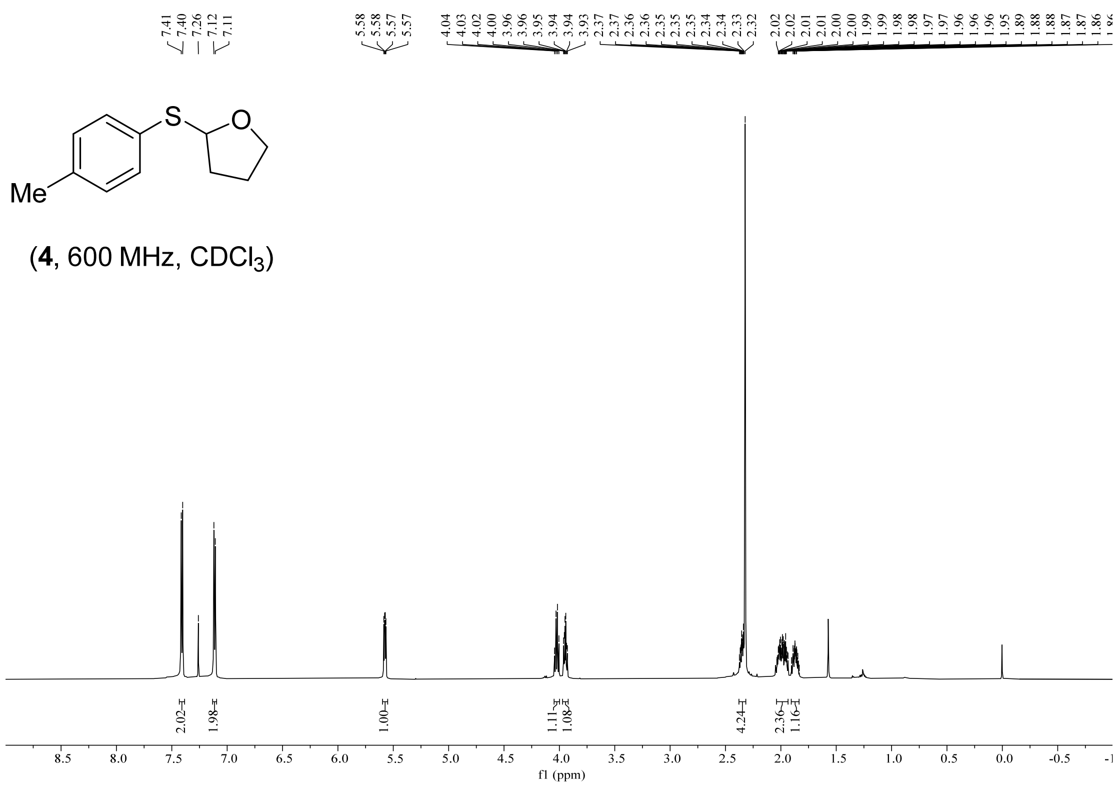
7.41
7.40
7.26
7.12
7.11
5.58
5.58
5.57
5.57
4.04
4.03
4.02
4.00
3.96
3.96
3.95
3.94
3.94
3.93
2.37
2.37
2.36
2.35
2.35
2.35
2.34
2.34
2.33
2.32
2.02
2.02
2.01
2.01
2.00
2.00
1.99
1.99
1.98
1.98
1.97
1.97
1.96
1.96
1.96
1.95
1.89
1.88
1.88
1.87
1.87
1.86
S
O
Me
(4, 600 MHz, CDCl3)
2.02
1.98
1.00
1.11
1.08
4.24
2.36
1.16
f1 (ppm)

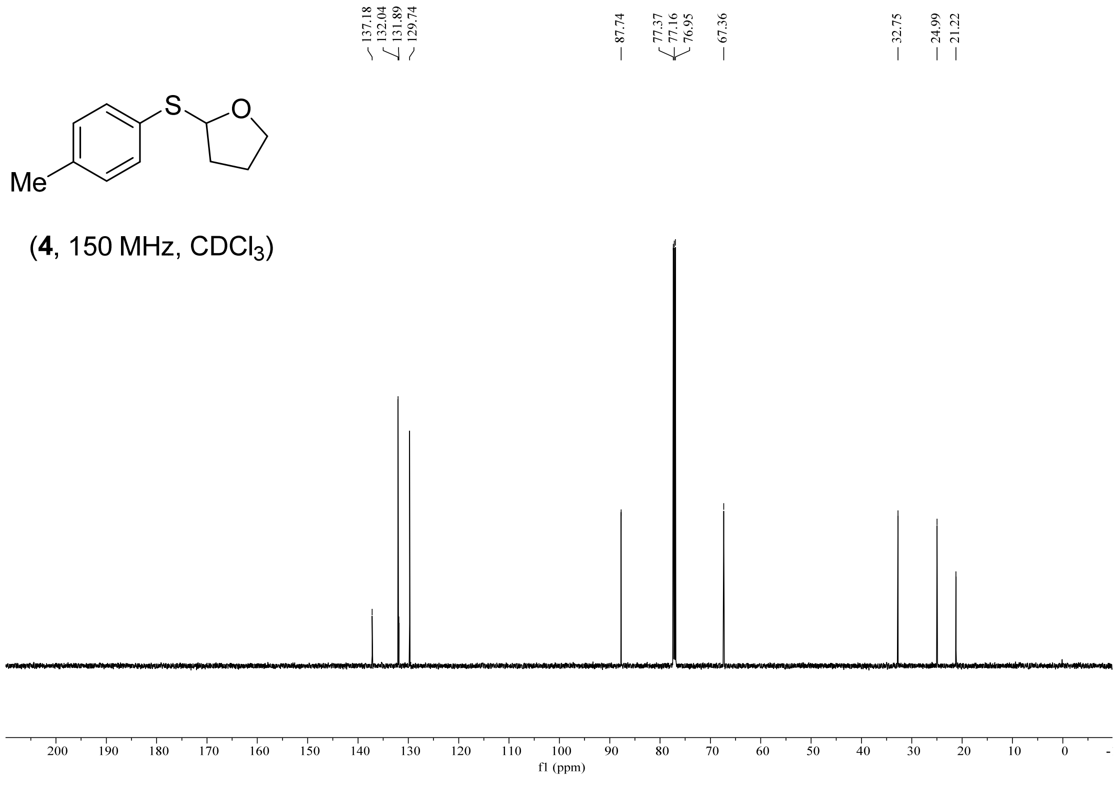
137.18
132.04
131.89
129.74
87.74
77.37
77.16
76.95
67.36
32.75
24.99
21.22
S
O
Me
(4, 150 MHz, CDCl3)
f1 (ppm)

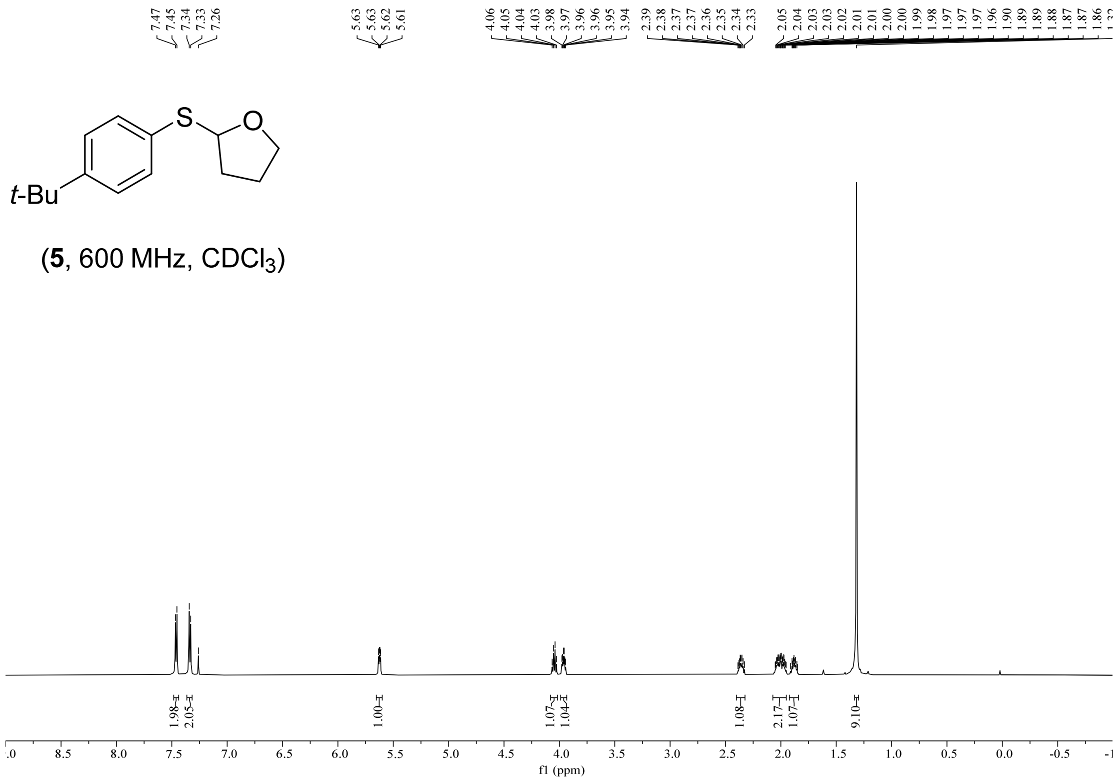
t-Bu
S
O
(5, 600 MHz, CDCl3)
f1 (ppm)

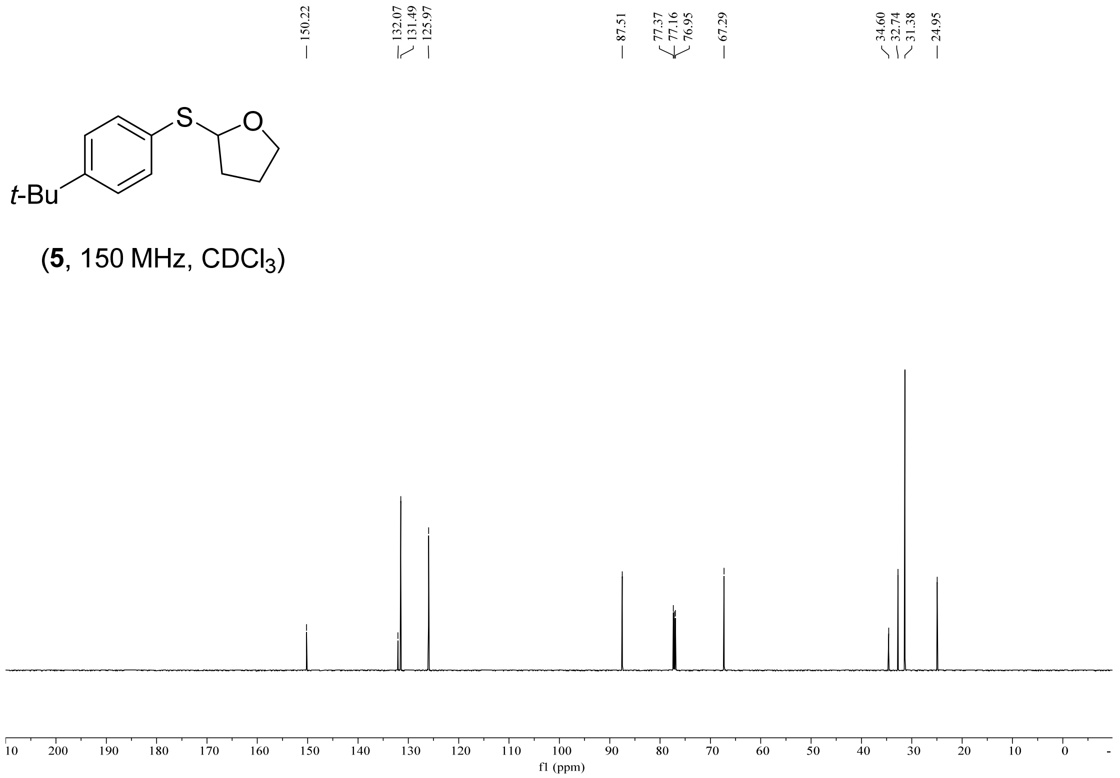
t-Bu
S
O
(5, 150 MHz, CDCl3)
f1 (ppm)

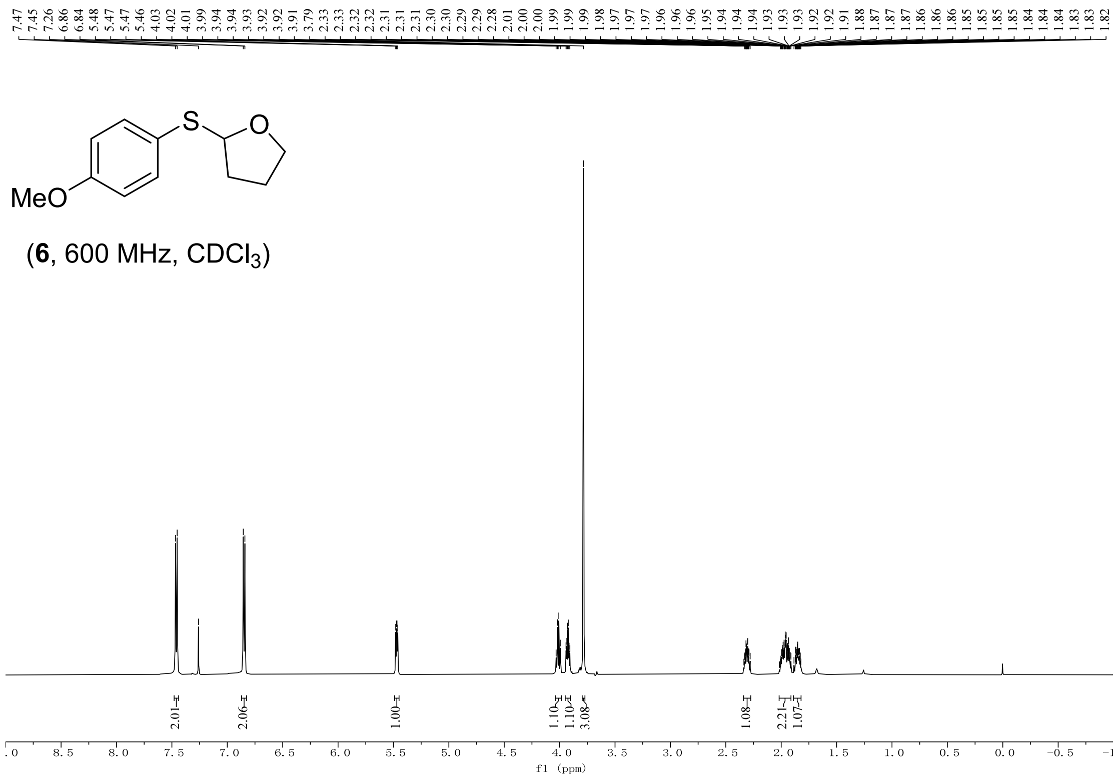
7.47 7.45 7.26 6.86 6.84 5.48 5.47 5.46 4.03 4.02 4.01 3.99 3.94 3.94 3.93 3.92 3.92 3.91 3.79 2.33 2.32 2.32 2.31 2.31 2.31 2.30 2.30 2.29 2.29 2.28 2.01 2.00 2.00 1.99 1.99 1.99 1.98 1.97 1.97 1.97 1.96 1.96 1.96 1.95 1.94 1.94 1.94 1.93 1.93 1.93 1.93 1.92 1.92 1.91 1.88 1.87 1.87 1.87 1.86 1.86 1.86 1.85 1.85 1.85 1.84 1.84 1.84 1.83 1.82
MeO
S
O
(**6**, 600 MHz, $CDCl_3$)
2.01
2.06
1.00
1.10
1.10
3.08
1.08
2.21
1.07
.0 8.5 8.0 7.5 7.0 6.5 6.0 5.5 5.0 4.5 4.0 3.5 3.0 2.5 2.0 1.5 1.0 0.5 0.0 −0.5 −1
f1 (ppm)

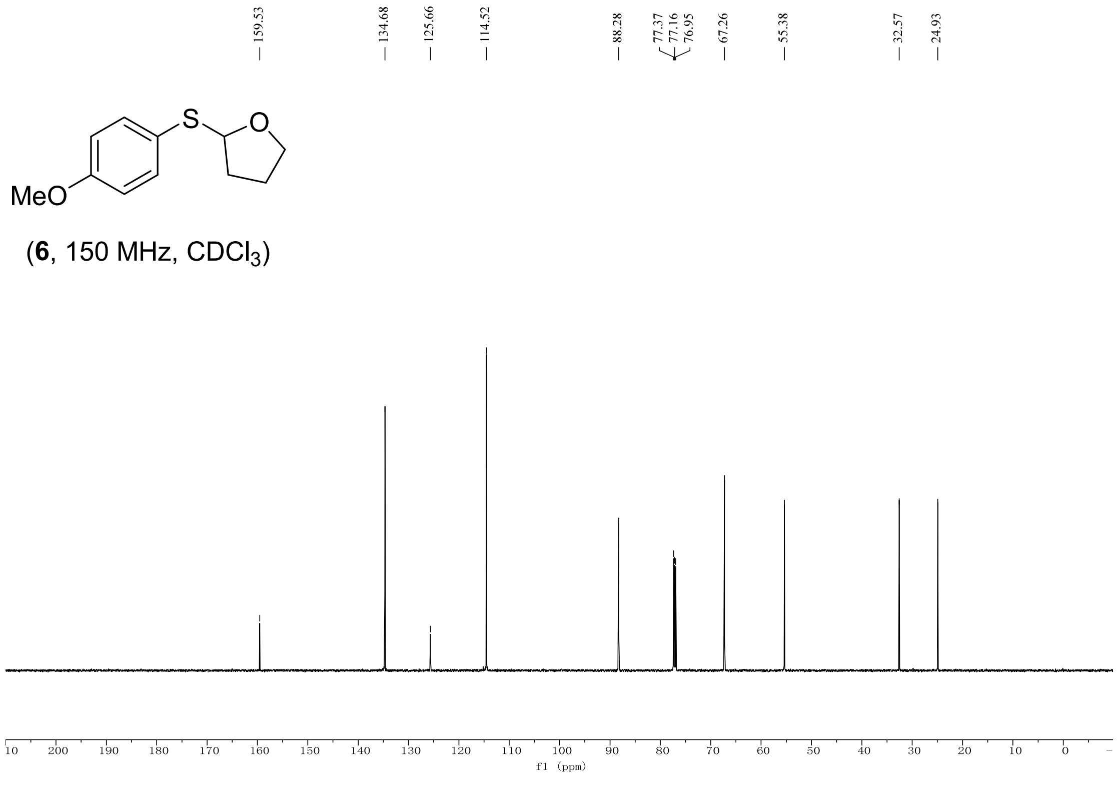
159.53
134.68
125.66
114.52
88.28
77.37
77.16
76.95
67.26
55.38
32.57
24.93
MeO
S
O
(**6**, 150 MHz, $CDCl_3$)
10 200 190 180 170 160 150 140 130 120 110 100 90 80 70 60 50 40 30 20 10 0
f1 (ppm)

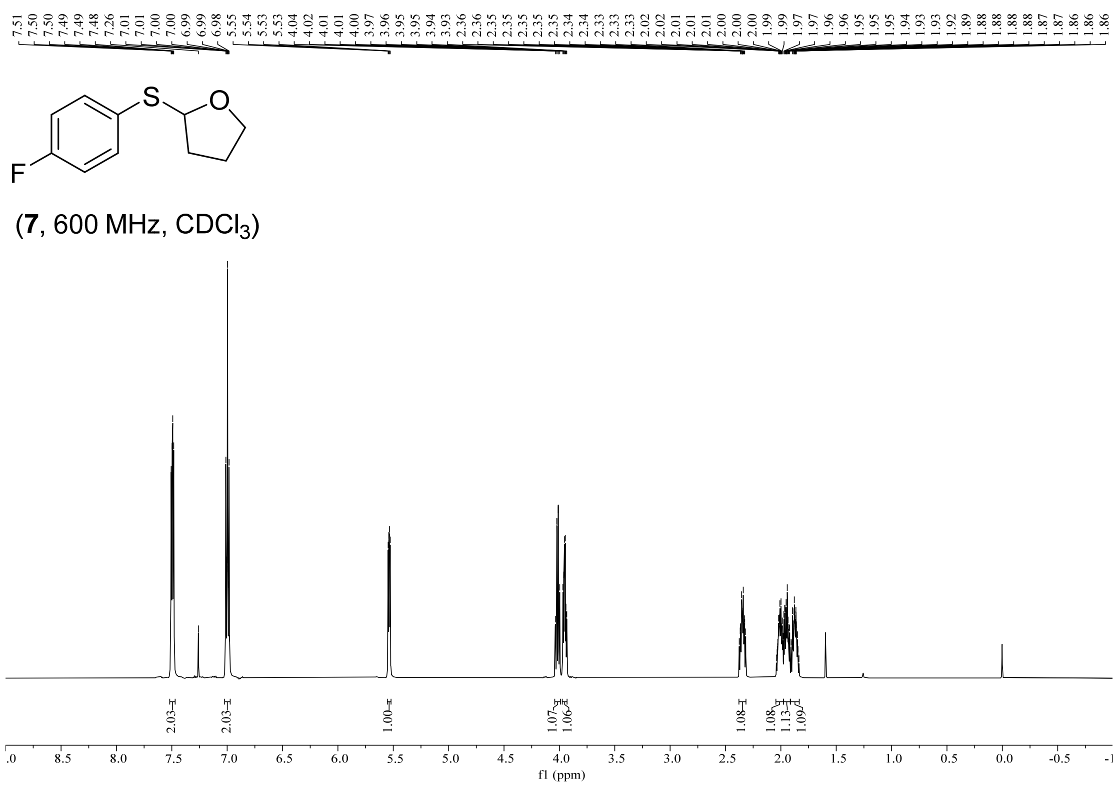

(**7**, 600 MHz, $CDCl_3$)

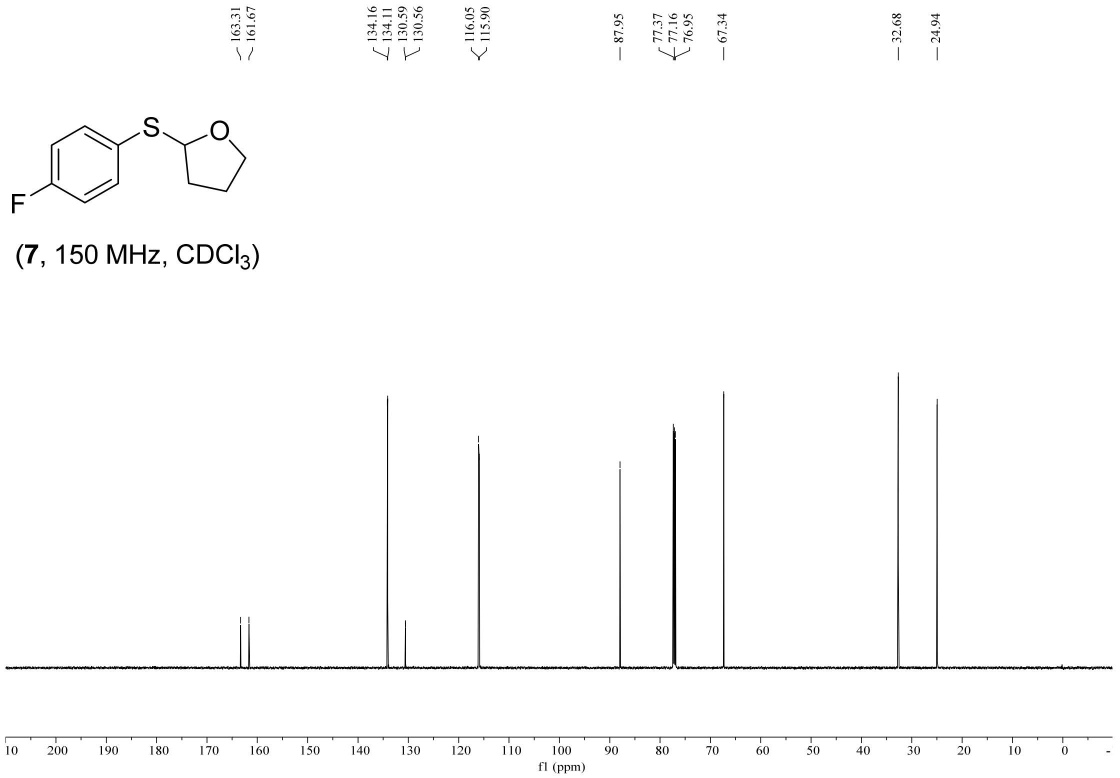

(**7**, 150 MHz, $CDCl_3$)

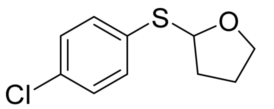

(**8**, 600 MHz, $CDCl_3$)

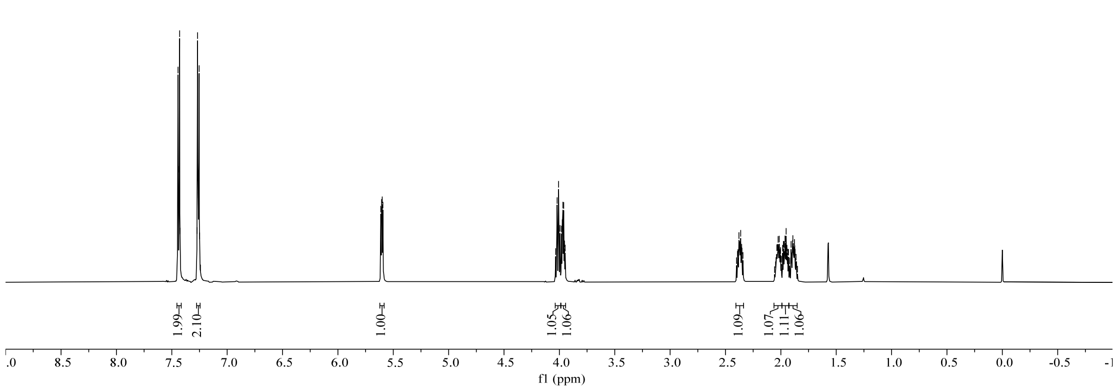

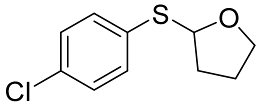

(**8**, 150 MHz, $CDCl_3$)

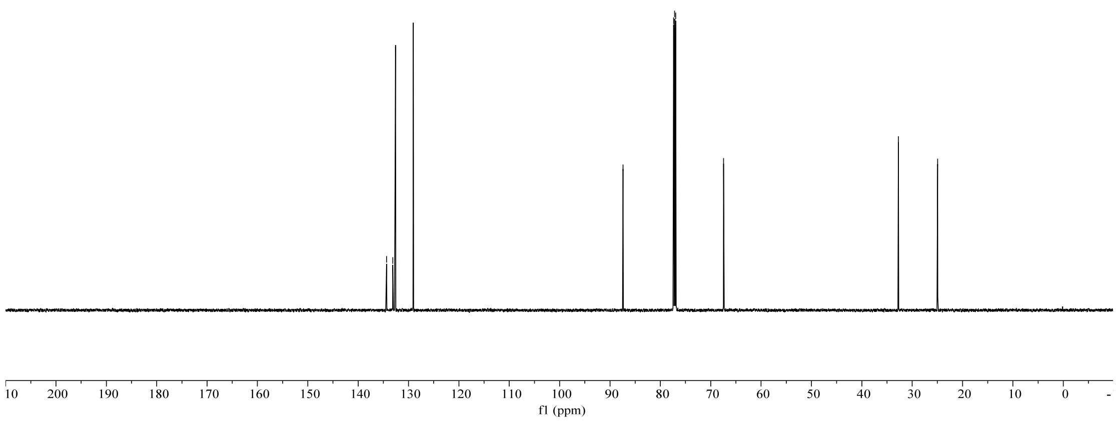

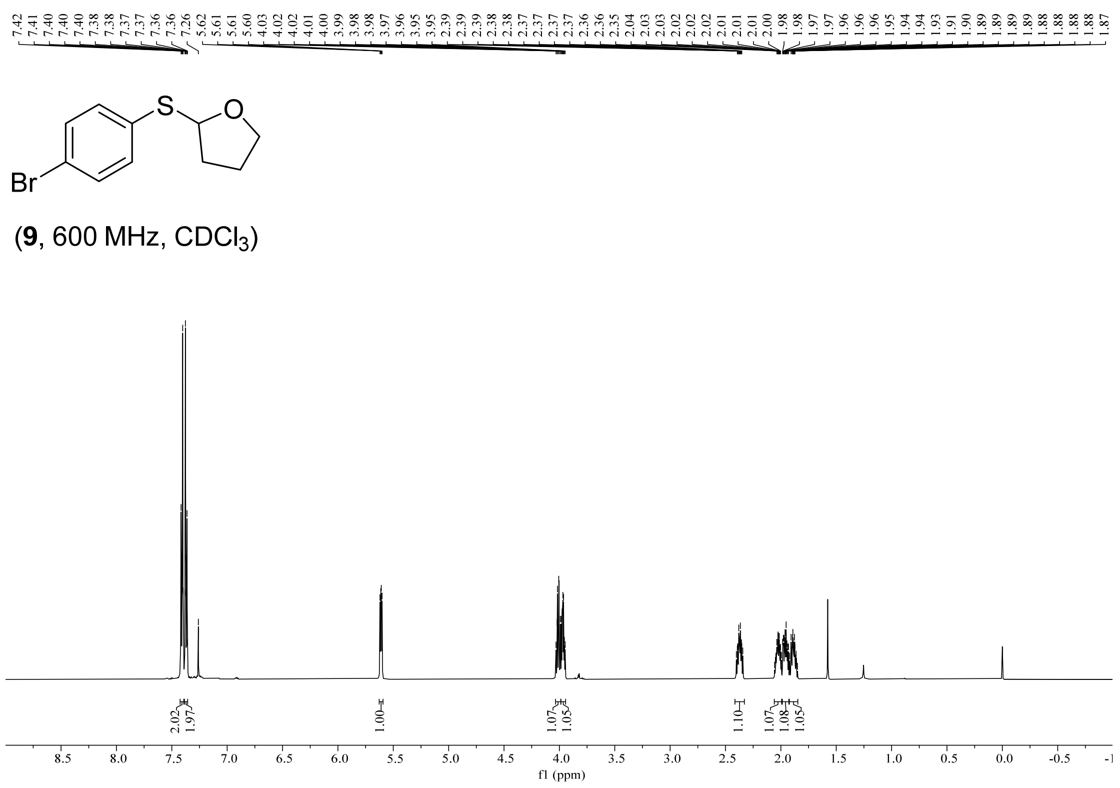

($\mathbf{9}$, 600 MHz, $CDCl_3$)

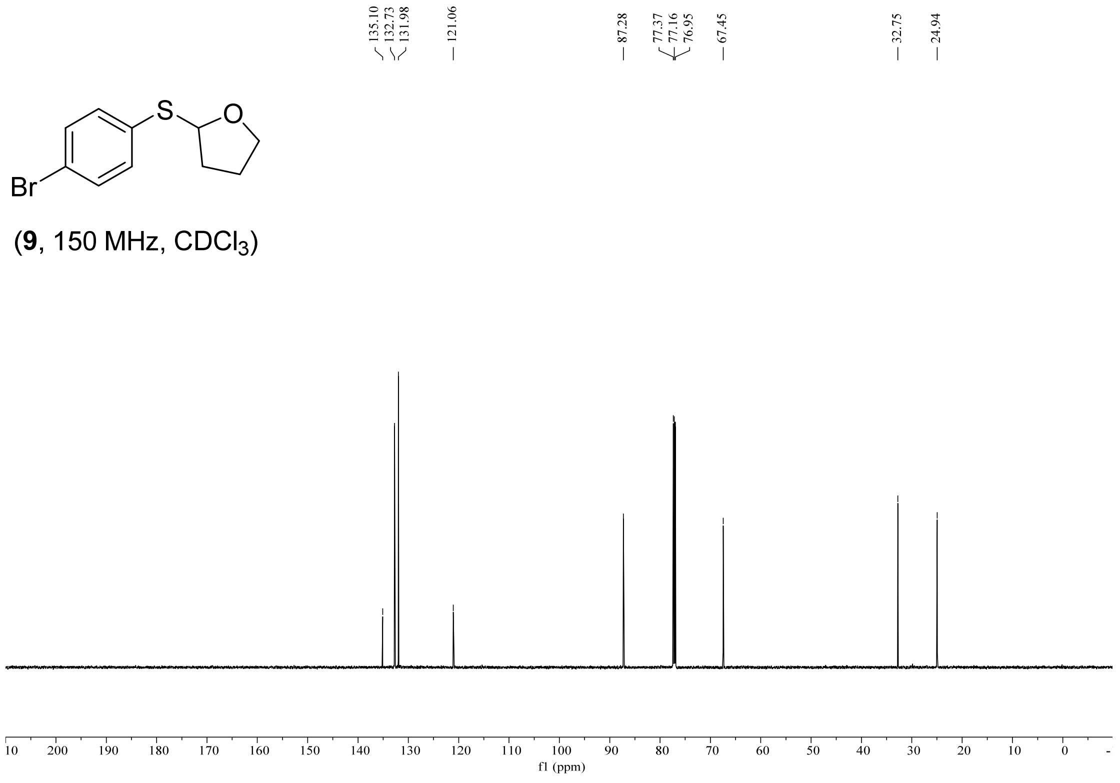

($\mathbf{9}$, 150 MHz, $CDCl_3$)

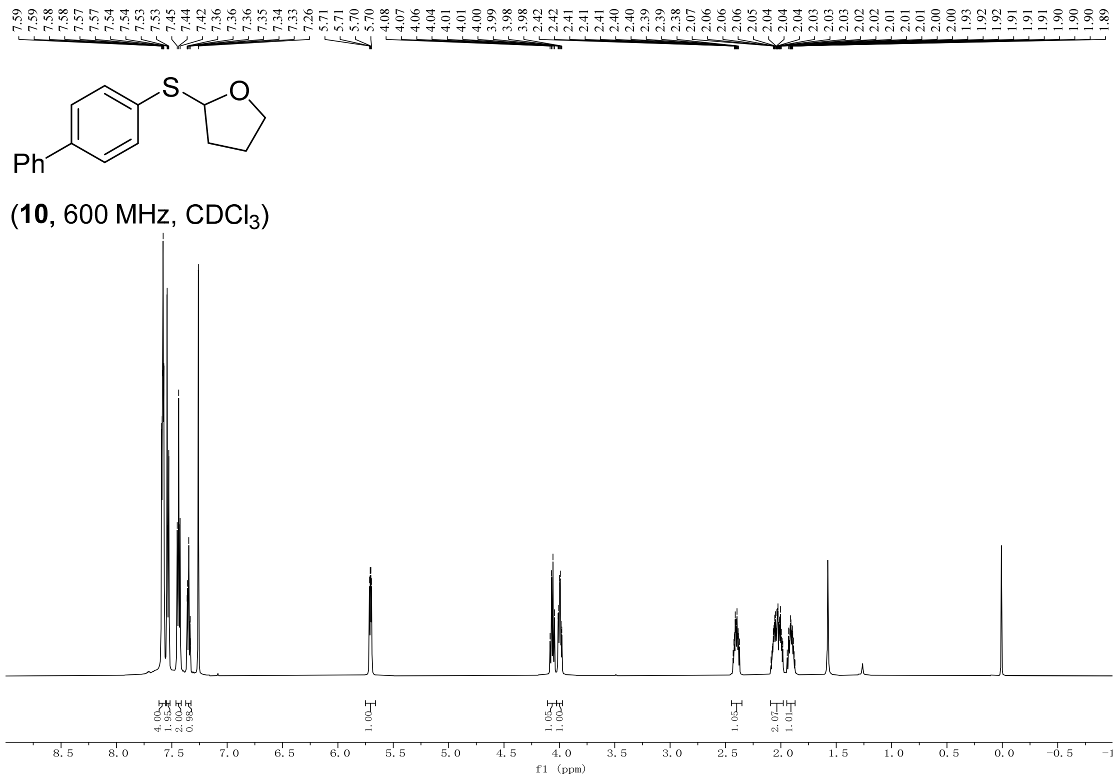

(**10**, 600 MHz, $CDCl_3$)

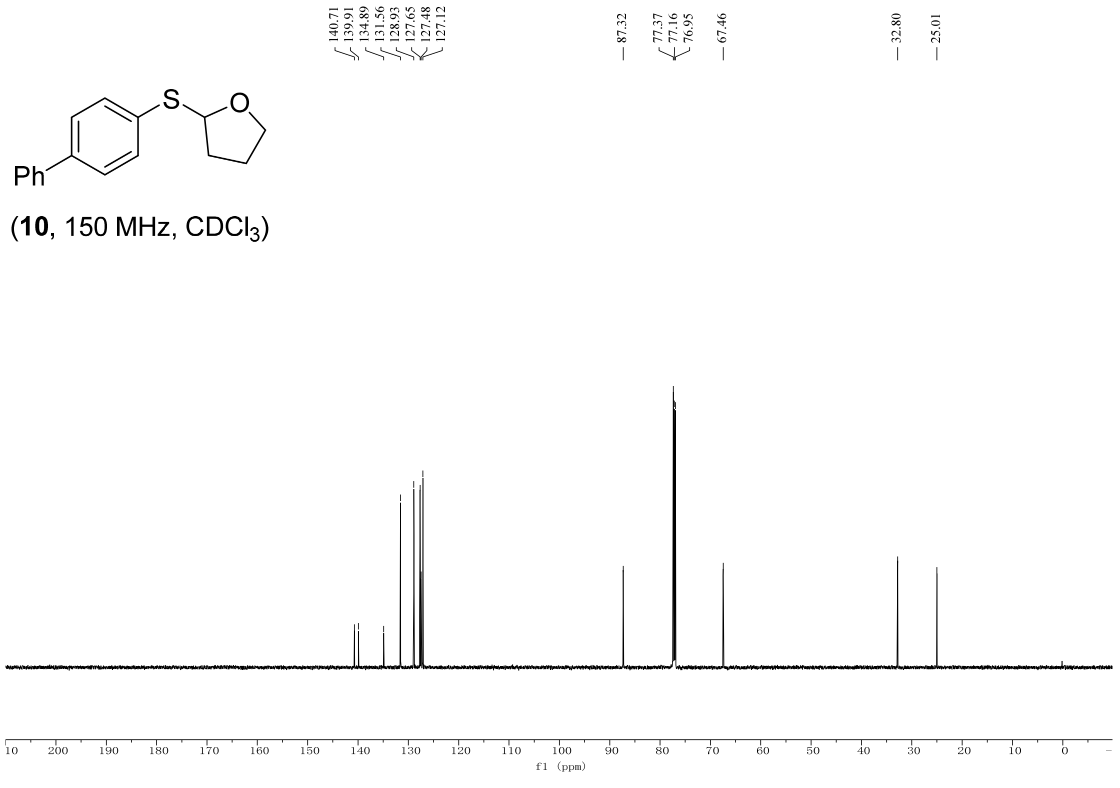

(**10**, 150 MHz, $CDCl_3$)

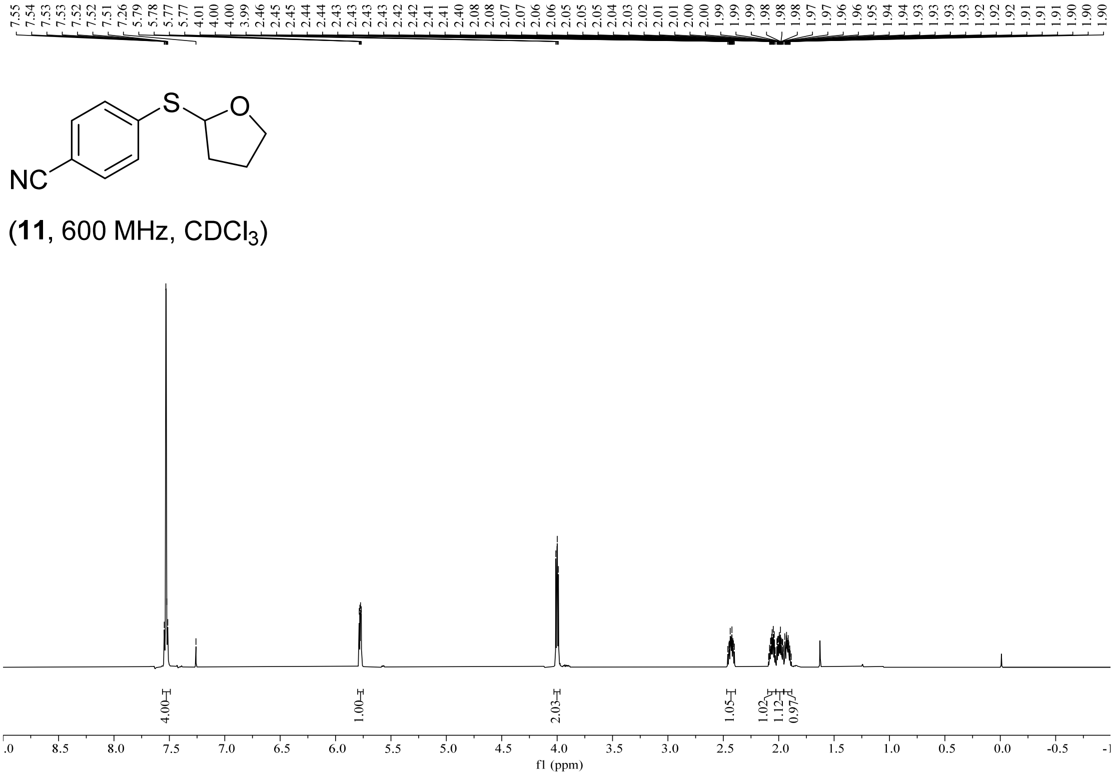

(**11**, 600 MHz, $CDCl_3$)

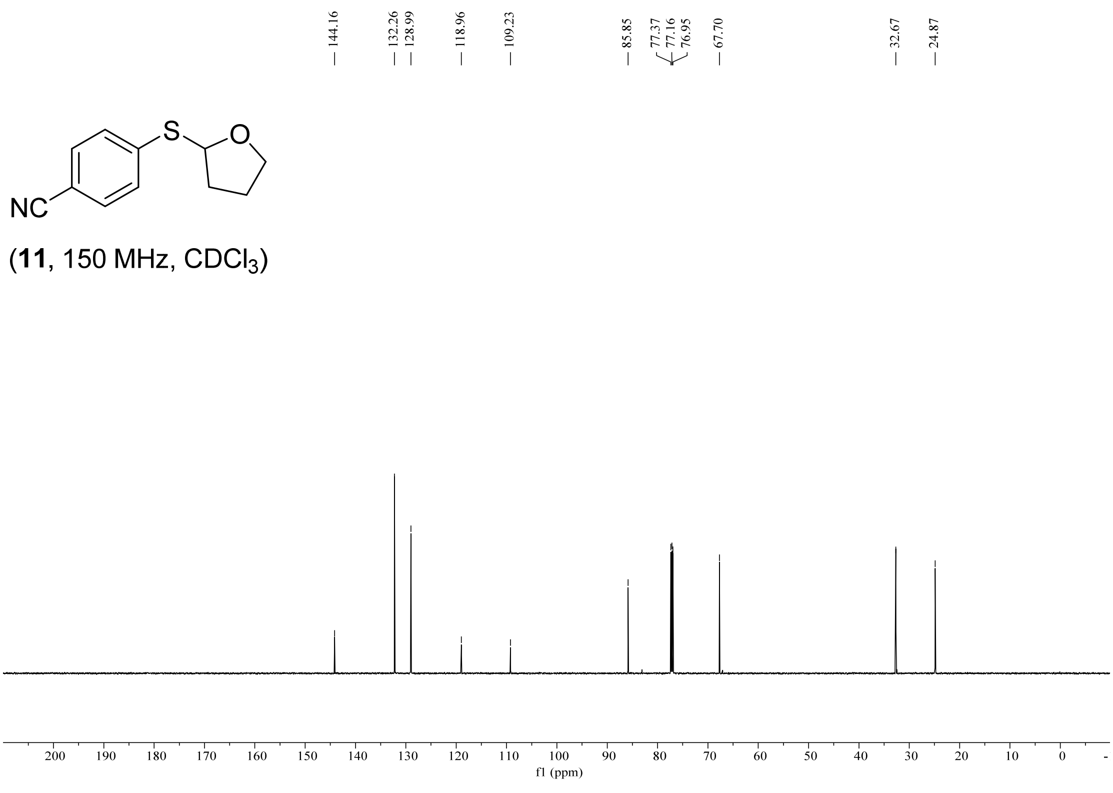

(**11**, 150 MHz, $CDCl_3$)

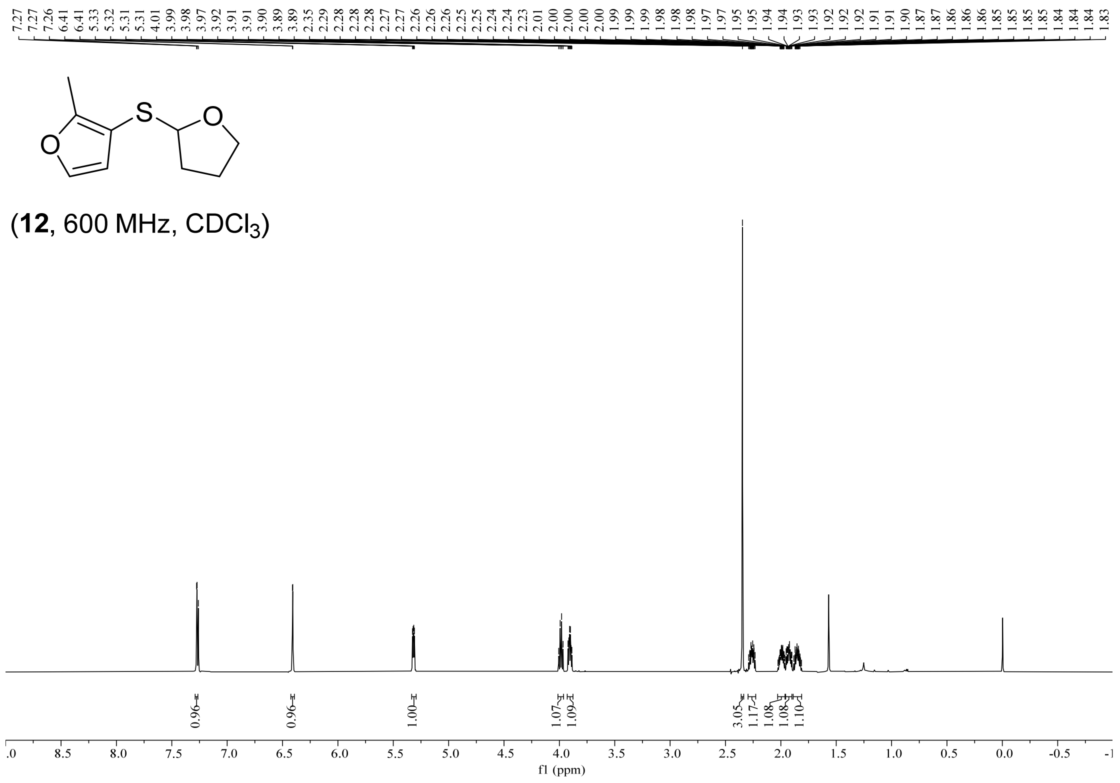

(**12**, 600 MHz, $CDCl_3$)

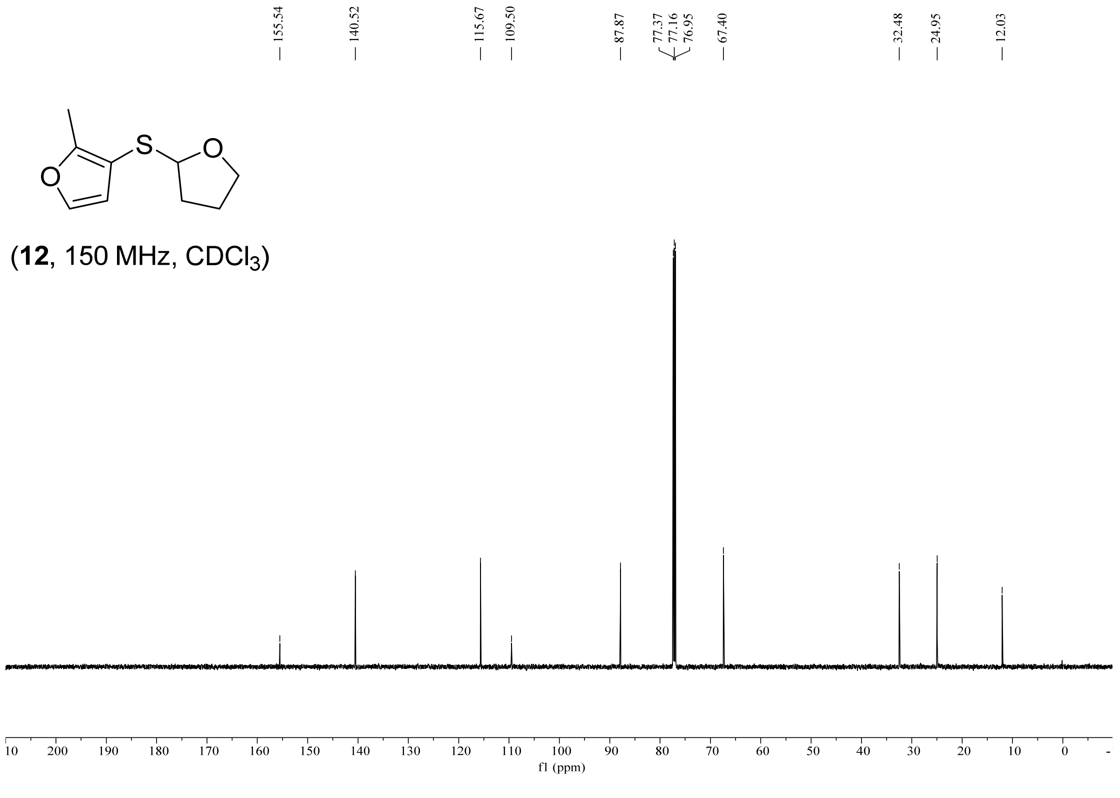

(**12**, 150 MHz, $CDCl_3$)

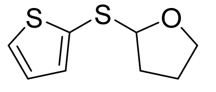

(**13**, 600 MHz, $CDCl_3$)

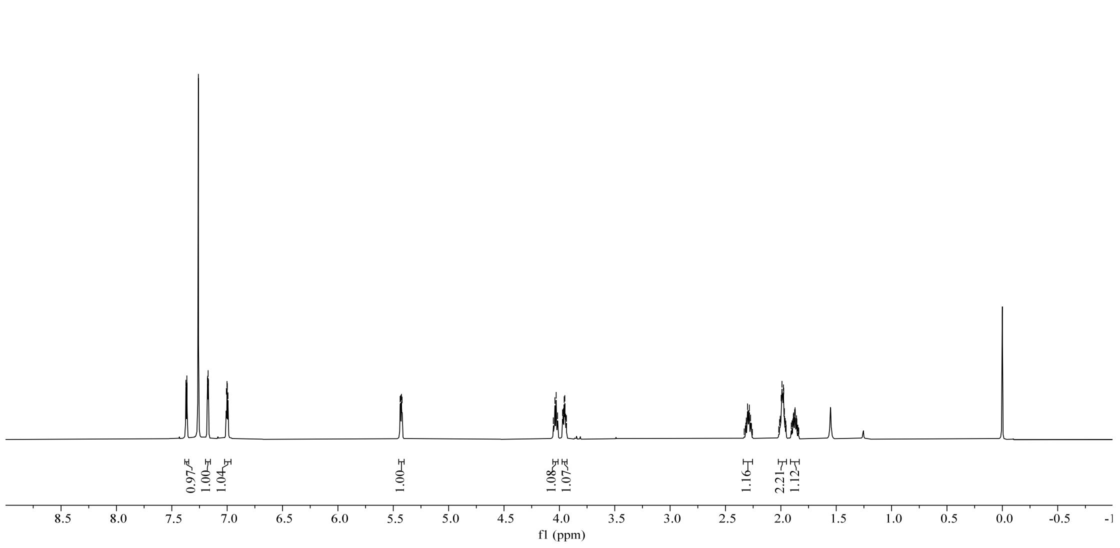

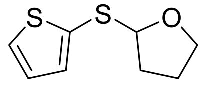

(**13**, 150 MHz, $CDCl_3$)

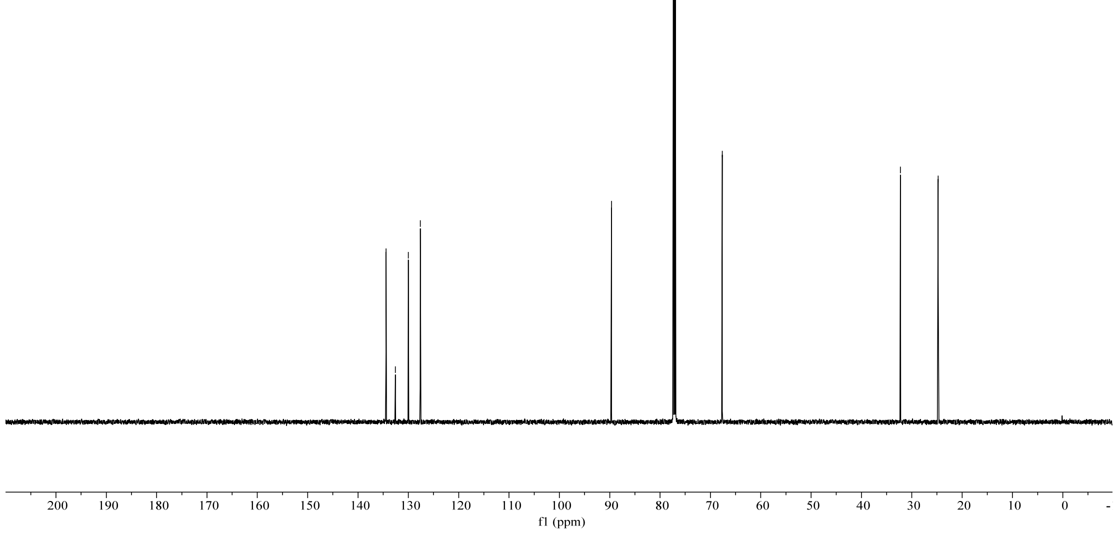

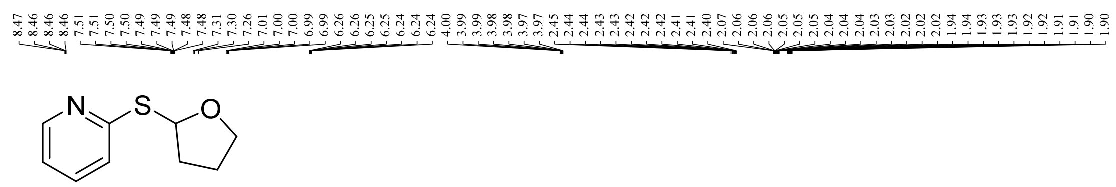

(**14**, 600 MHz, $CDCl_3$)

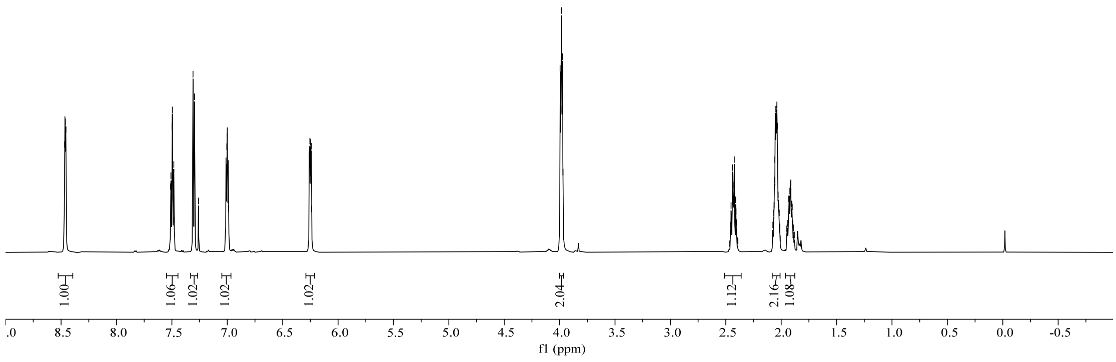

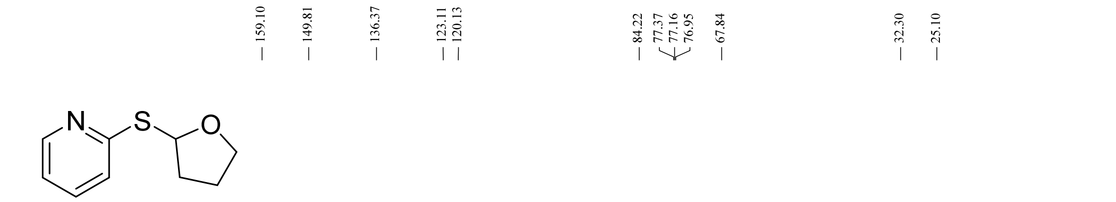

(**14**, 150 MHz, $CDCl_3$)

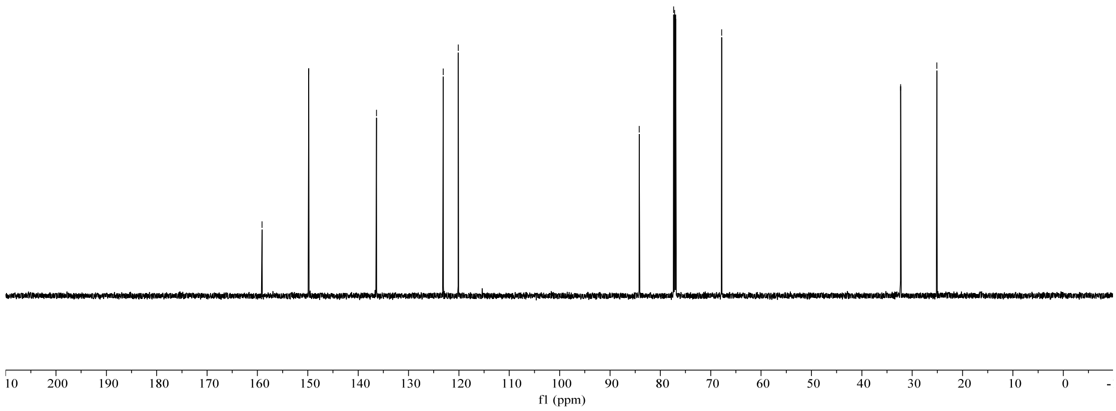

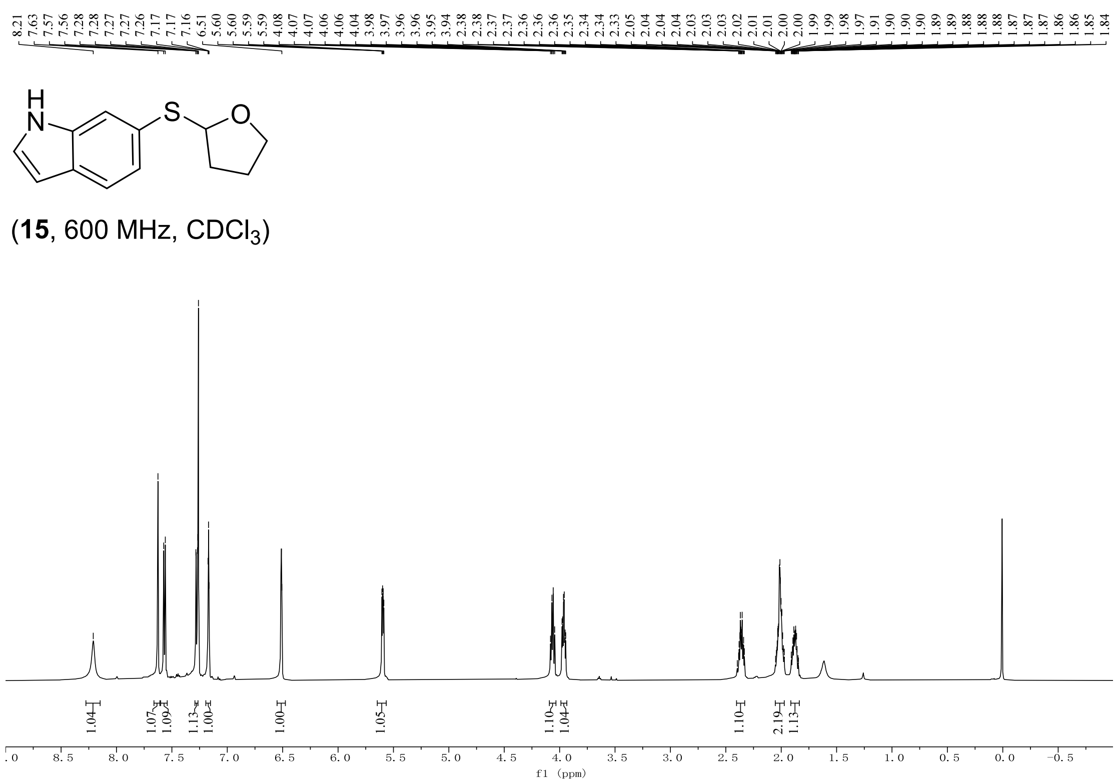

(**15**, 600 MHz, $CDCl_3$)

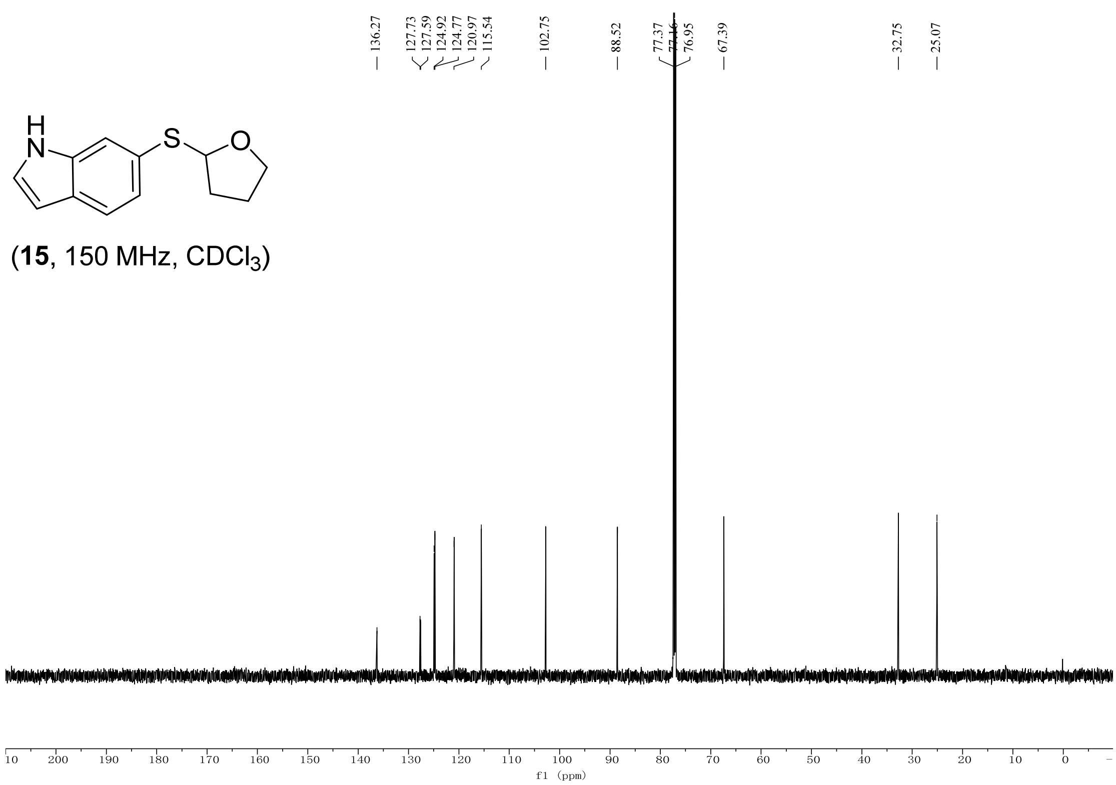

(**15**, 150 MHz, $CDCl_3$)

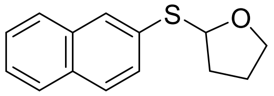

(**16**, 600 MHz, $CDCl_3$)

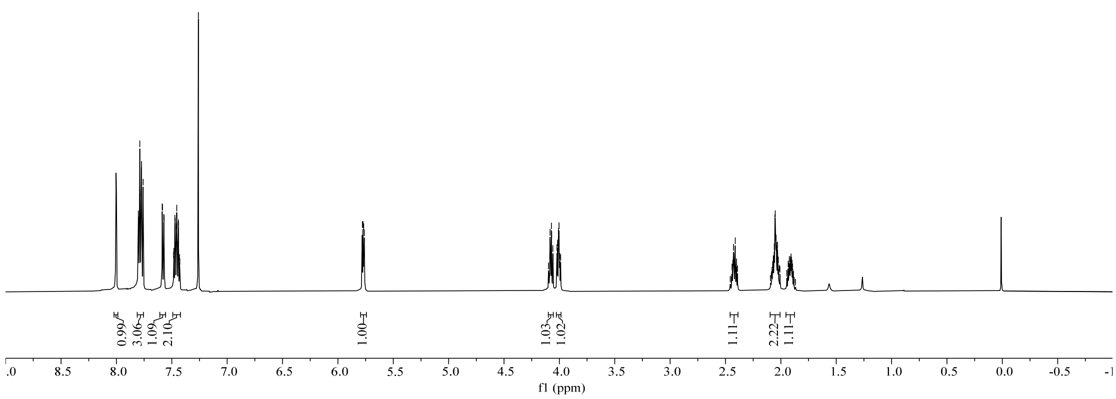

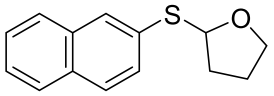

(**16**, 600 MHz, $CDCl_3$)

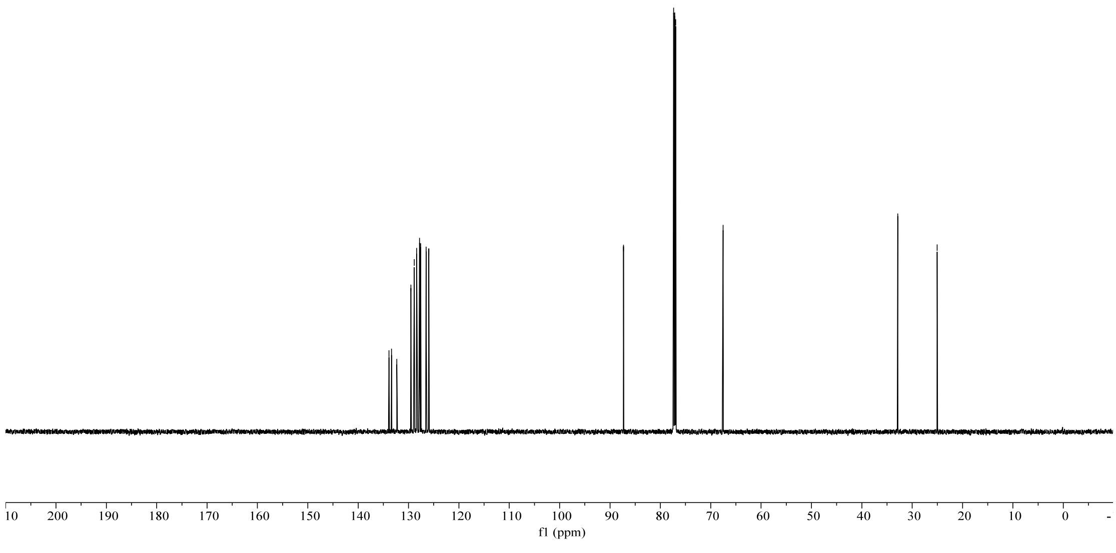

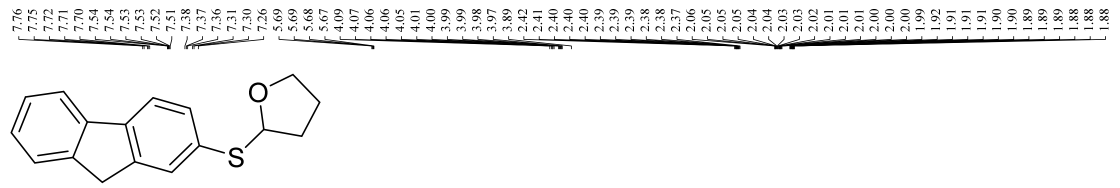

(**17**, 600 MHz, $CDCl_3$)

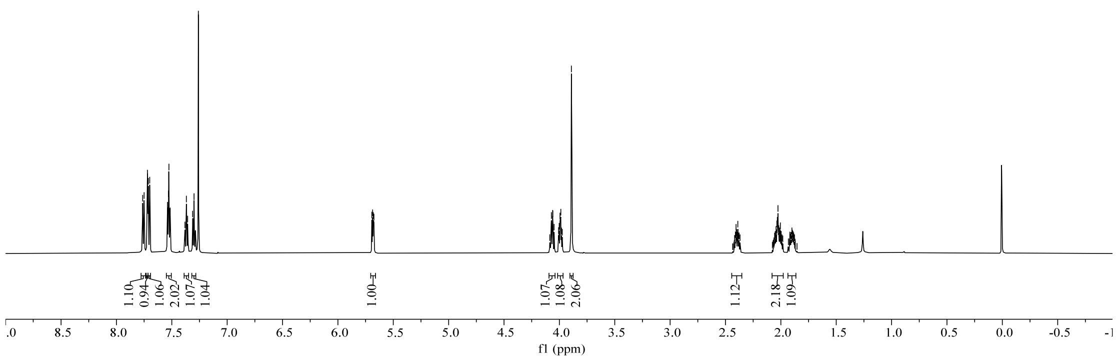

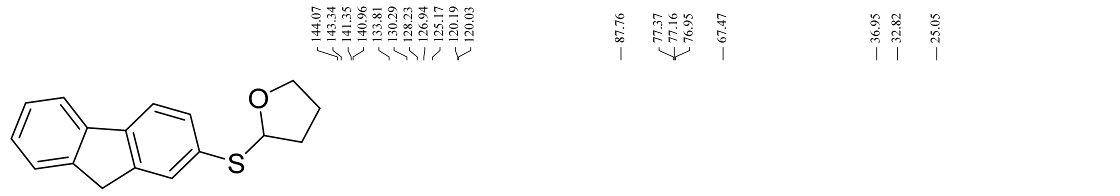

(**17**, 150 MHz, $CDCl_3$)

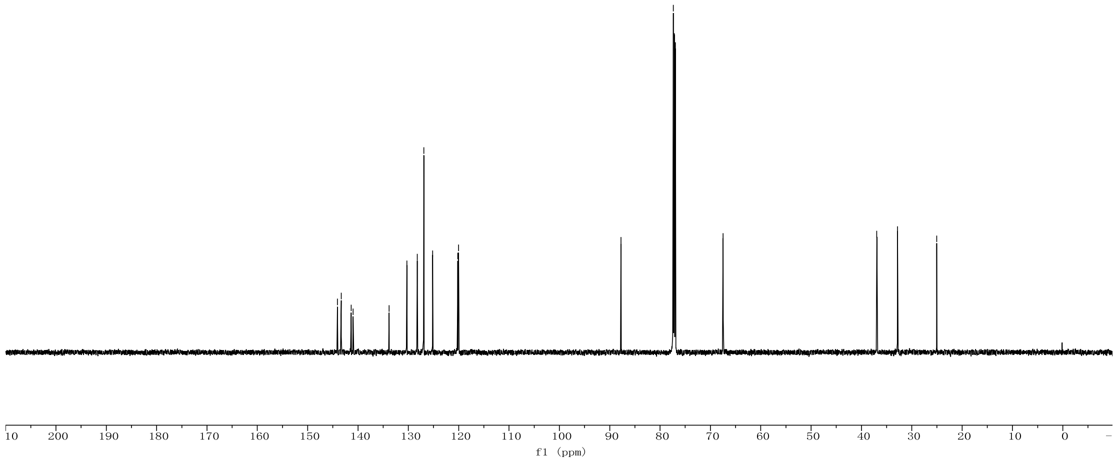

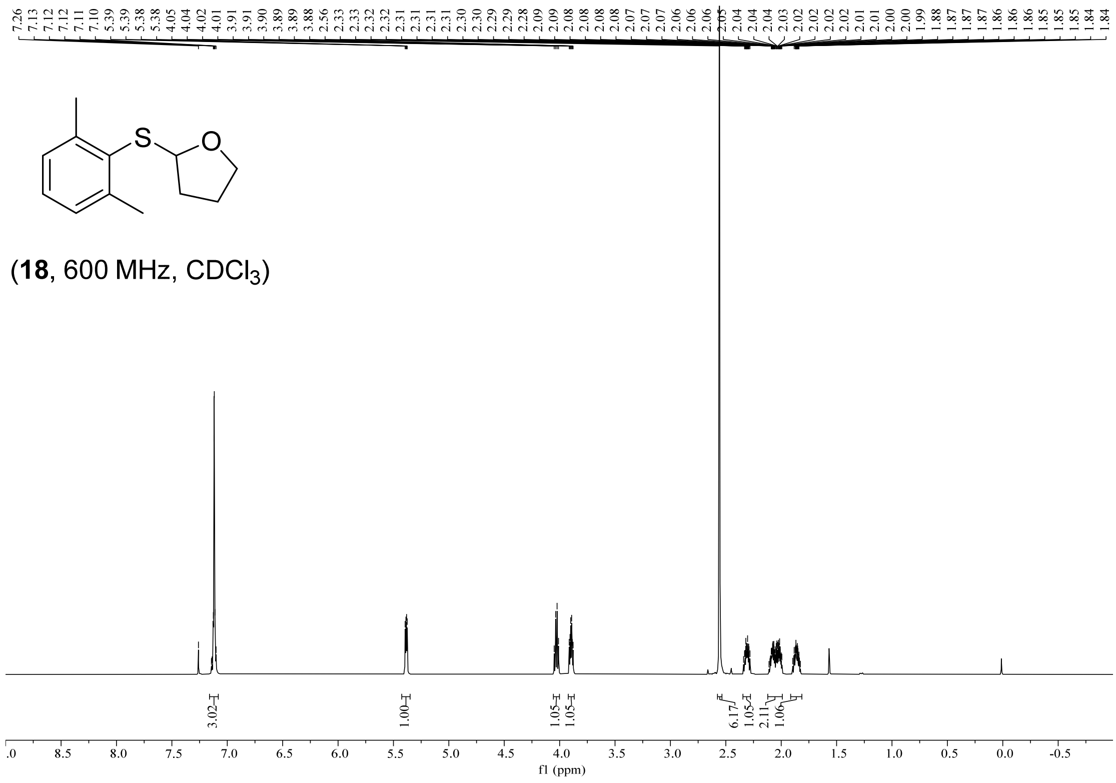

(**18**, 600 MHz, $CDCl_3$)

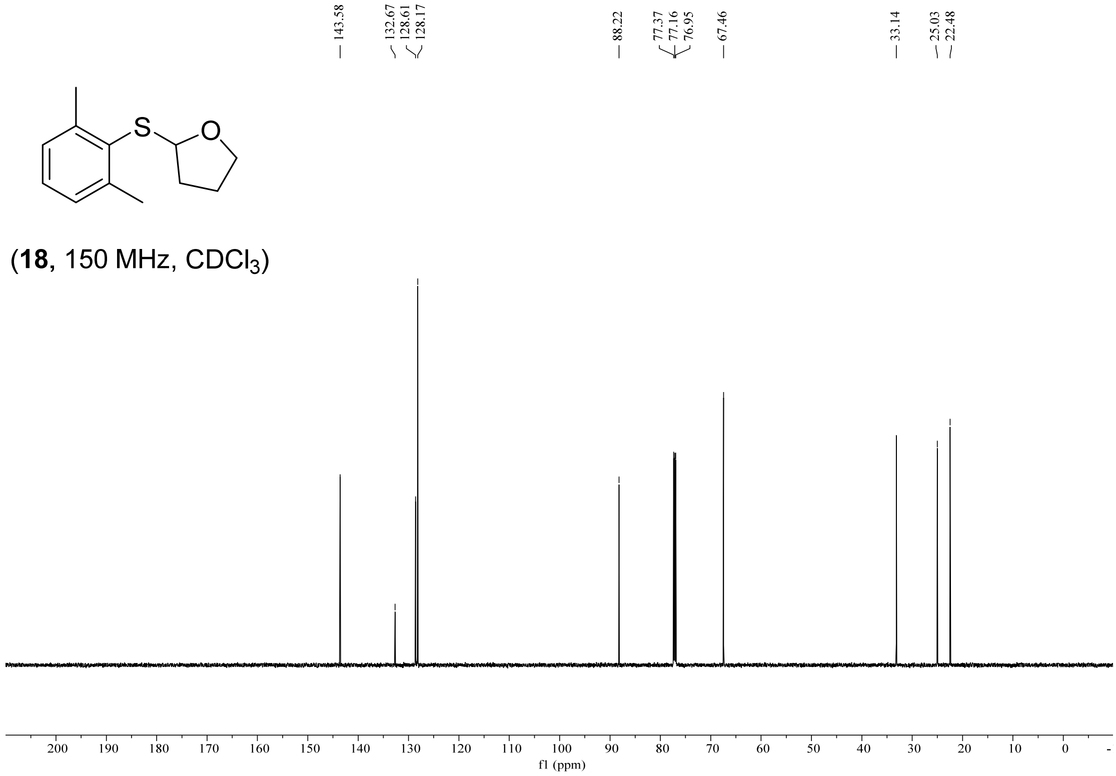

(**18**, 150 MHz, $CDCl_3$)

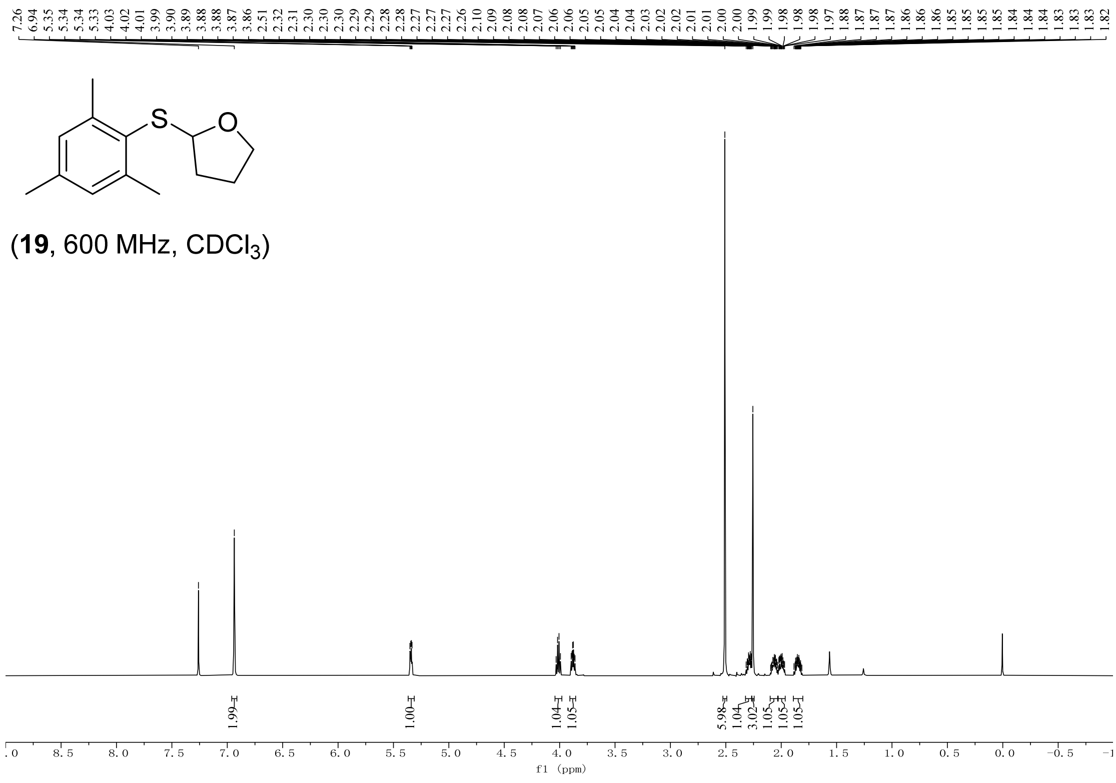

(**19**, 600 MHz, $CDCl_3$)

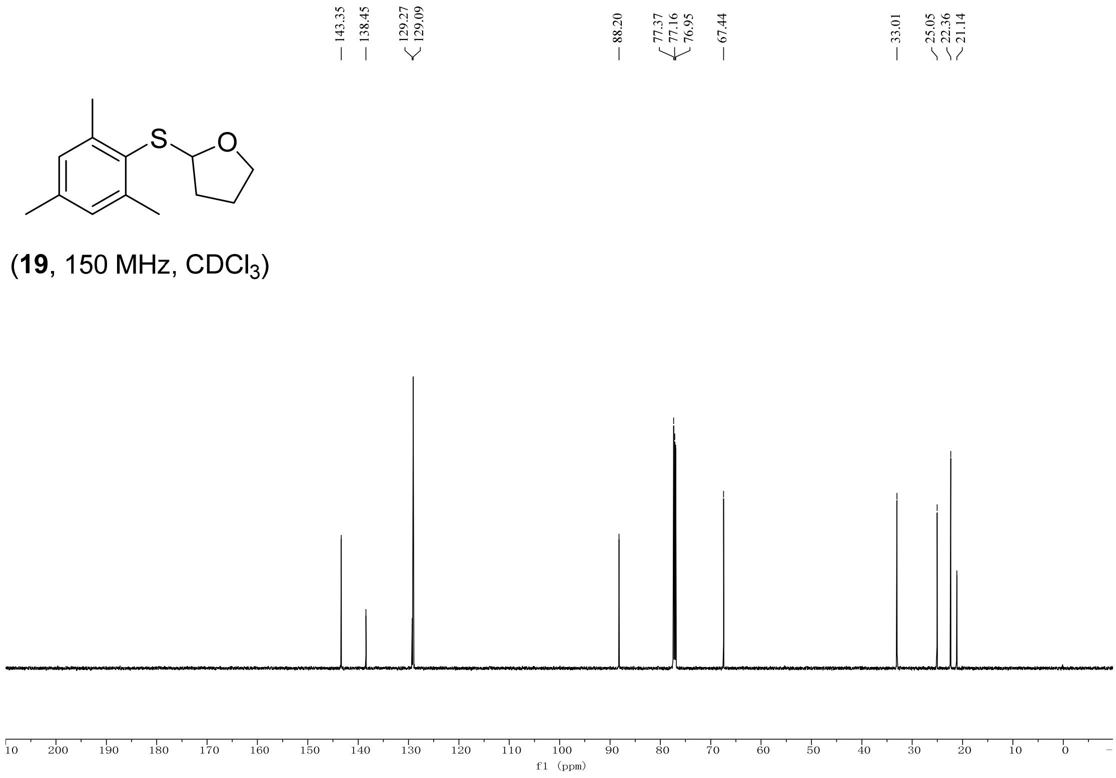

(**19**, 150 MHz, $CDCl_3$)

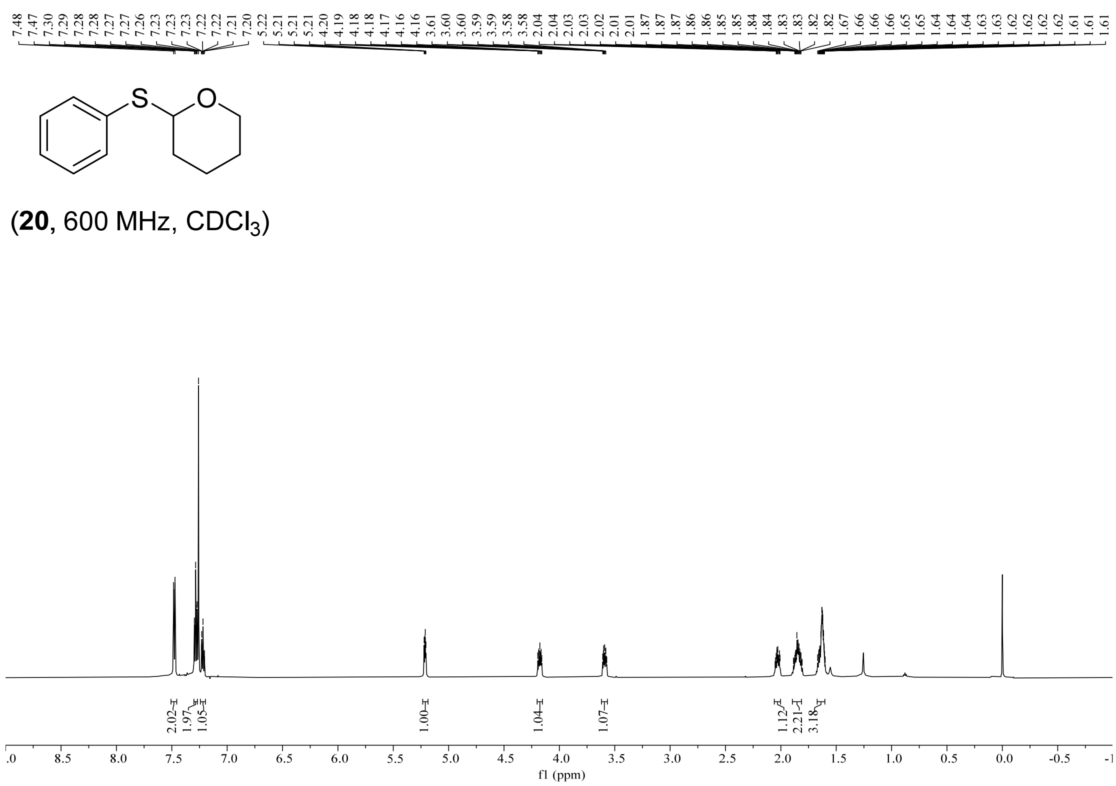

(**20**, 600 MHz, $CDCl_3$)

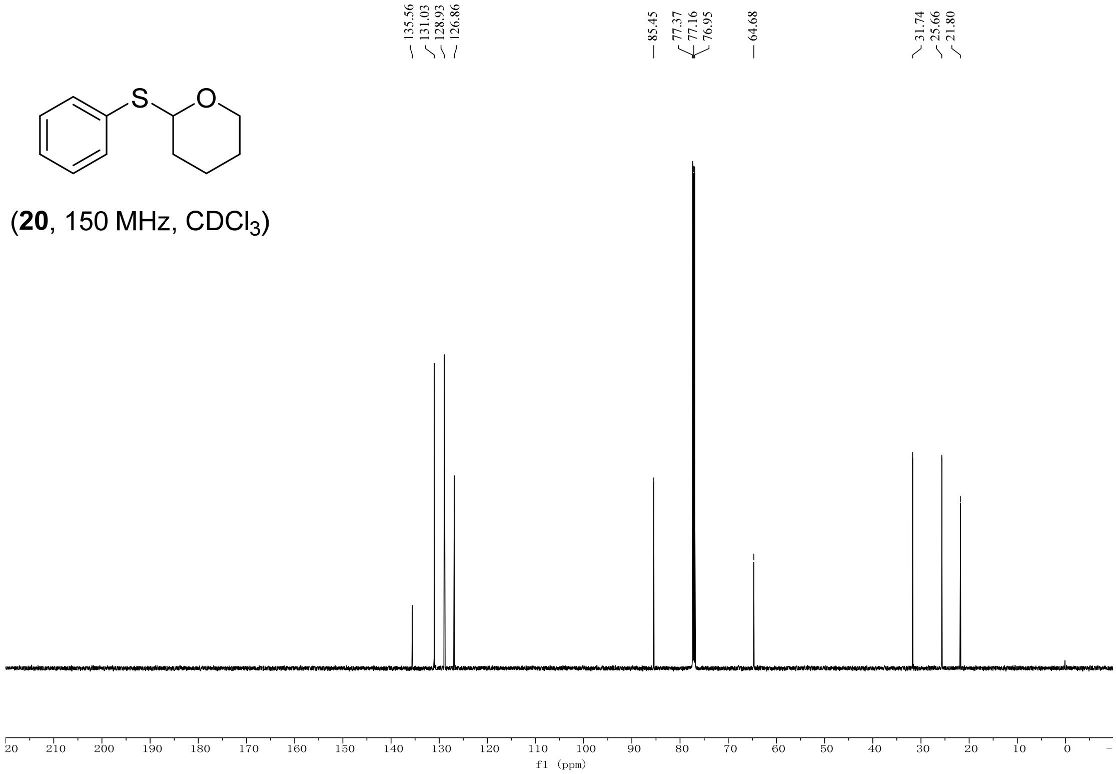

(**20**, 150 MHz, $CDCl_3$)

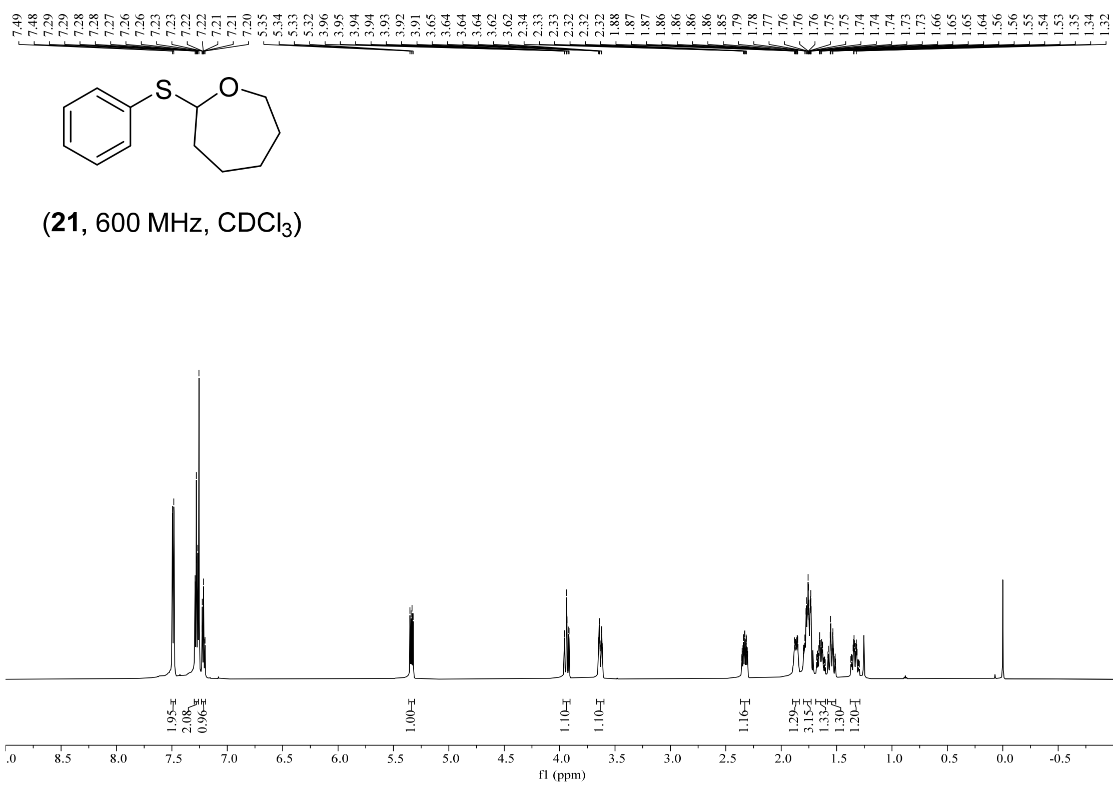

(**21**, 600 MHz, $CDCl_3$)

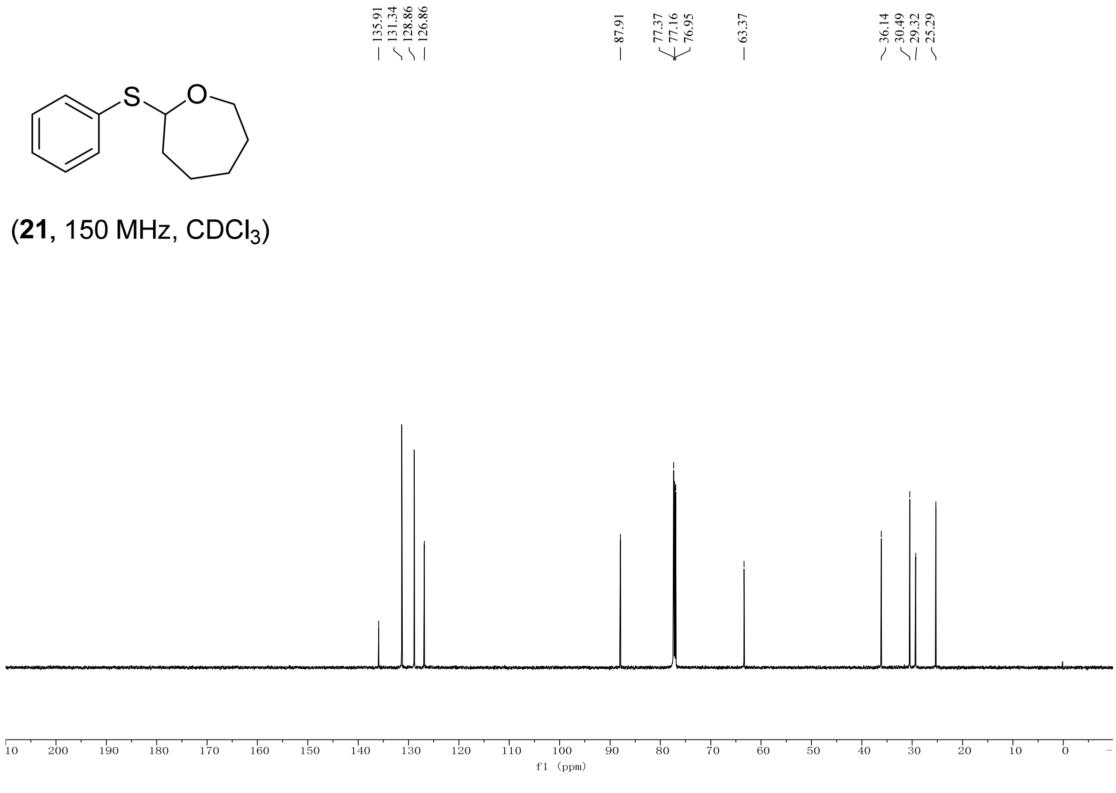

(**21,** 150 MHz, $CDCl_3$)

(**22**, 600 MHz, $CDCl_3$)

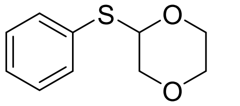

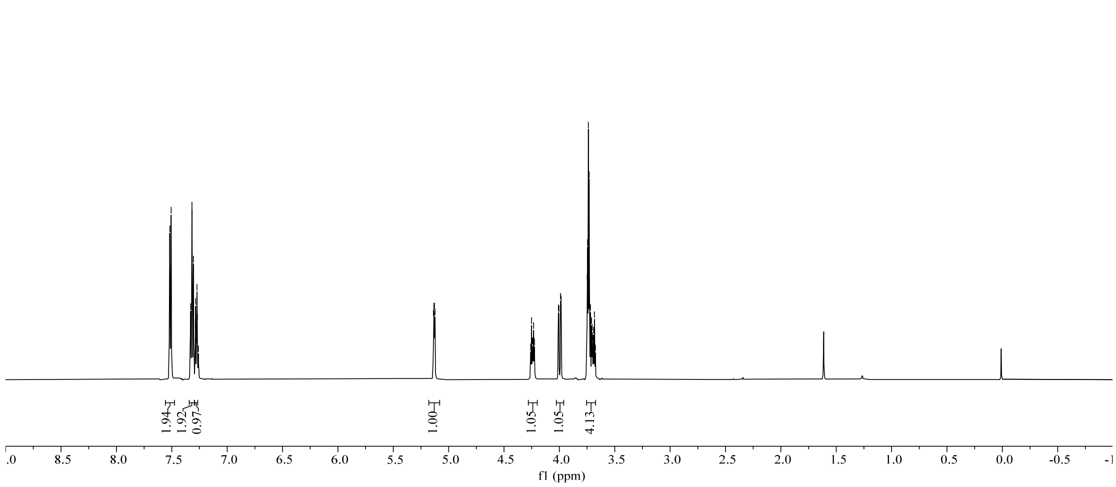

(**22**, 150 MHz, $CDCl_3$)

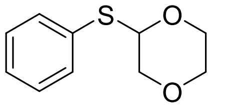

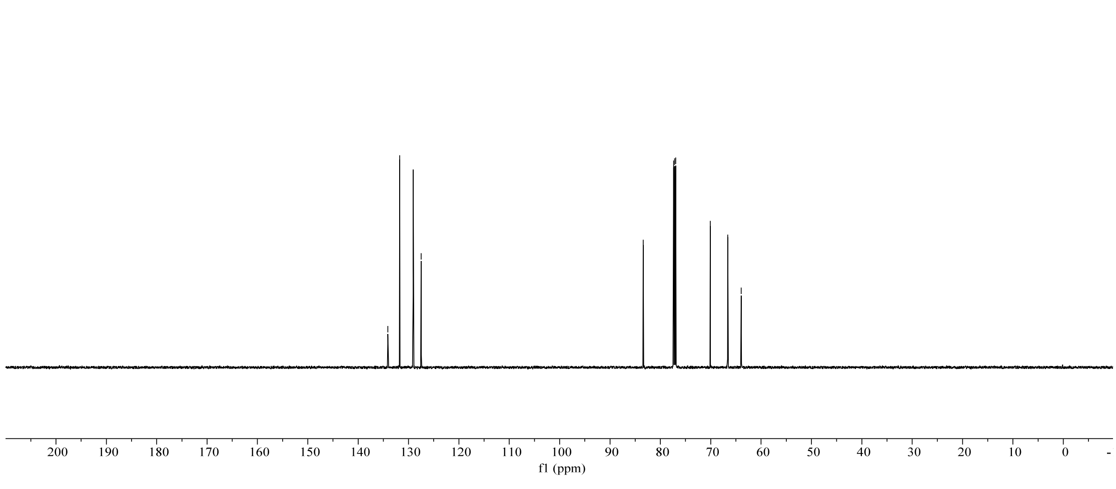

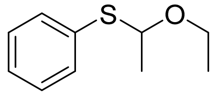

(**23**, 600 MHz, $CDCl_3$)

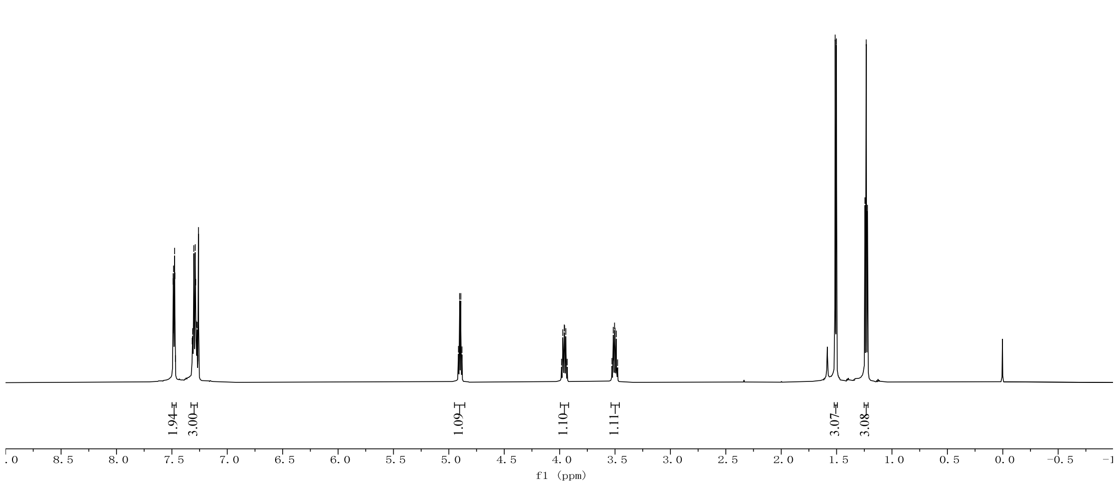

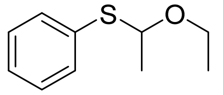

(**23**, 150 MHz, $CDCl_3$)

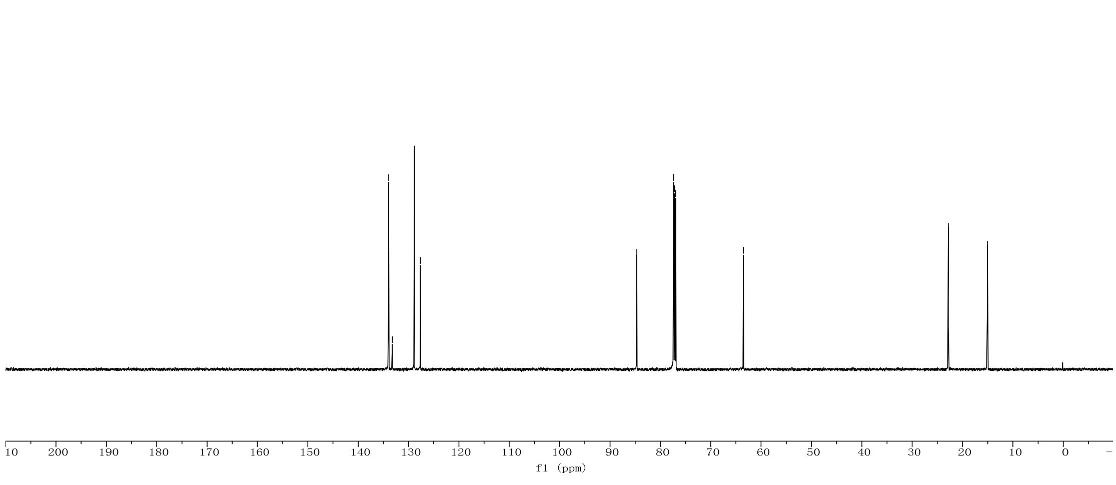

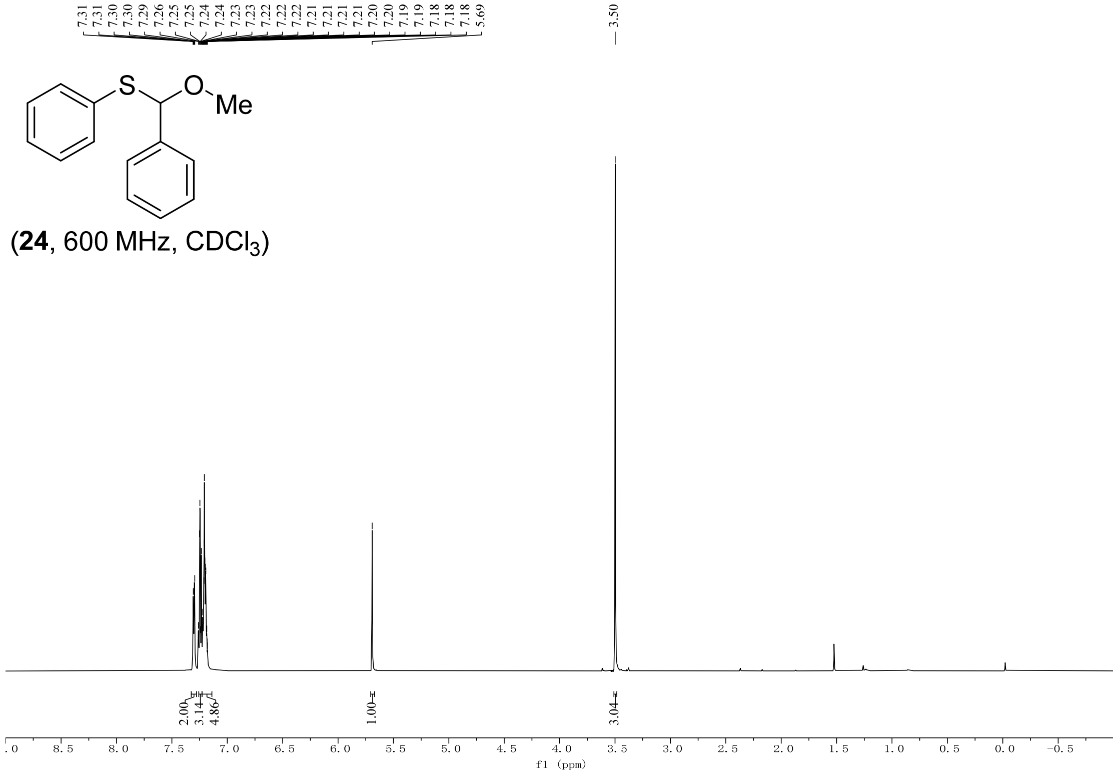

(**24**, 600 MHz, $CDCl_3$)

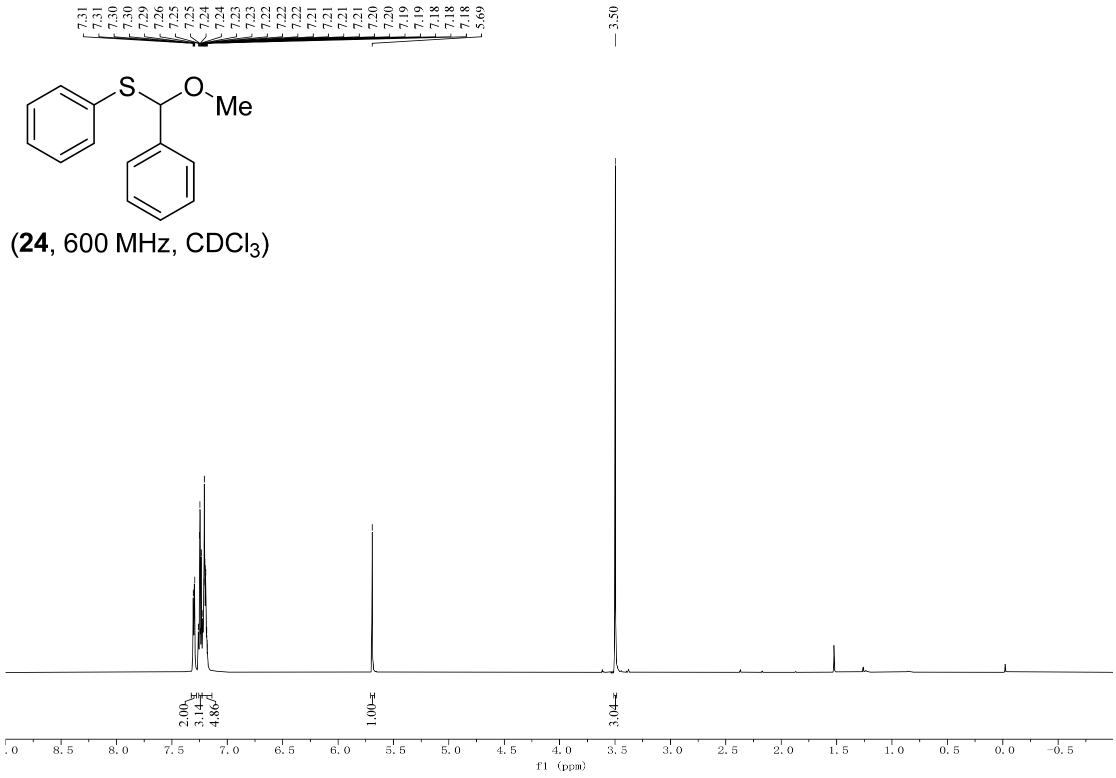

(**24**, 600 MHz, $CDCl_3$)

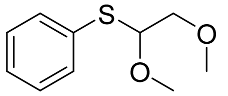

(**25**, 600 MHz, $CDCl_3$)

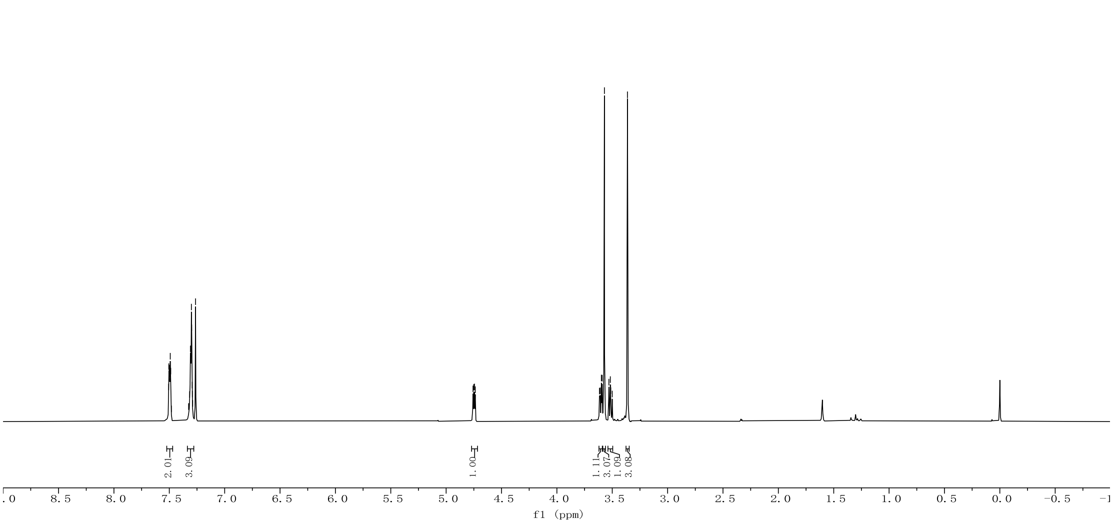

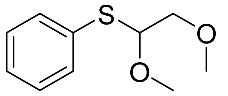

(**25**, 150 MHz, $CDCl_3$)

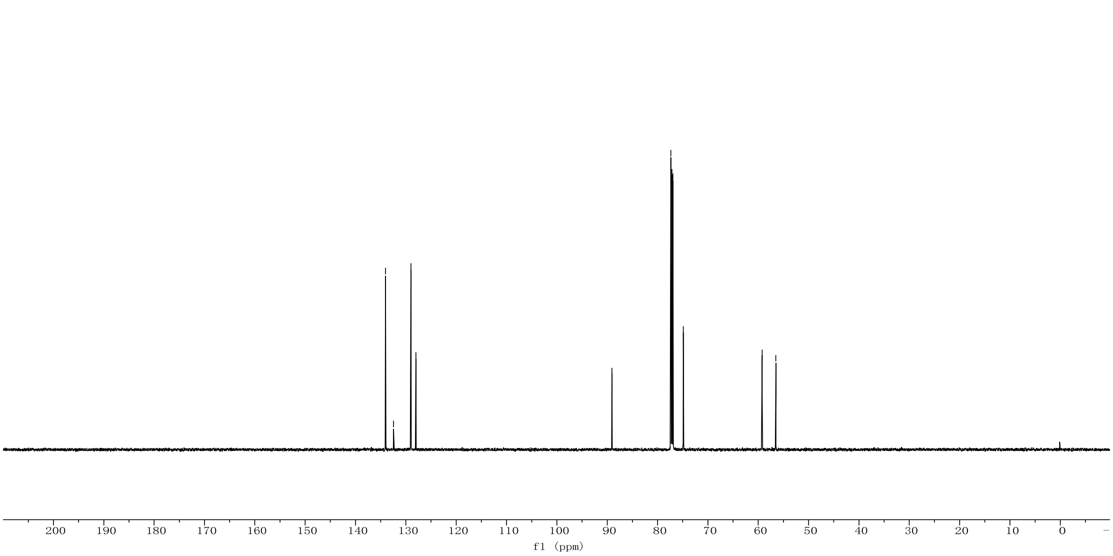

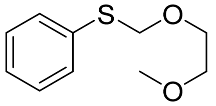

(**26**, 600 MHz, $CDCl_3$)

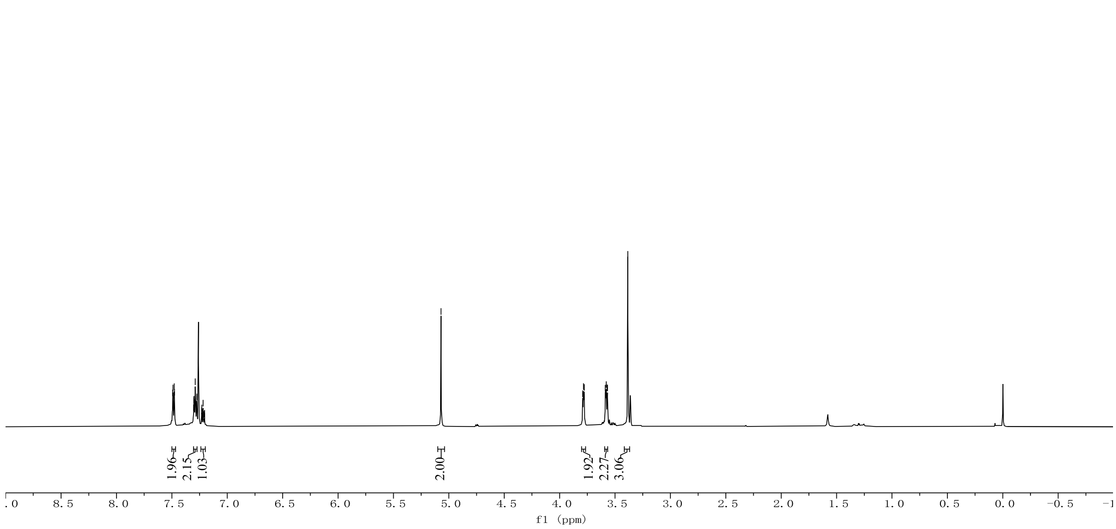

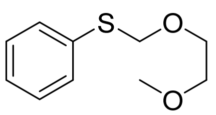

(**26**, 150 MHz, $CDCl_3$)

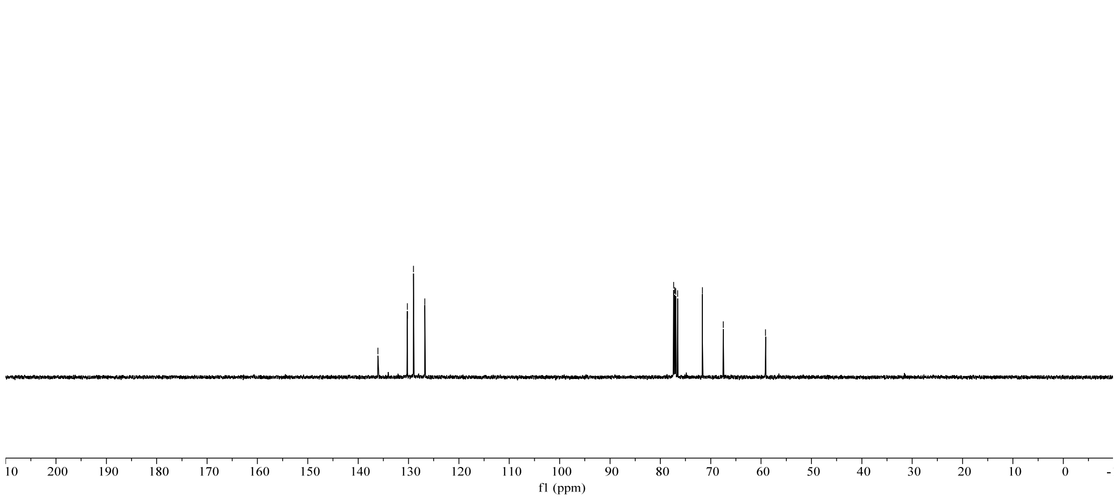

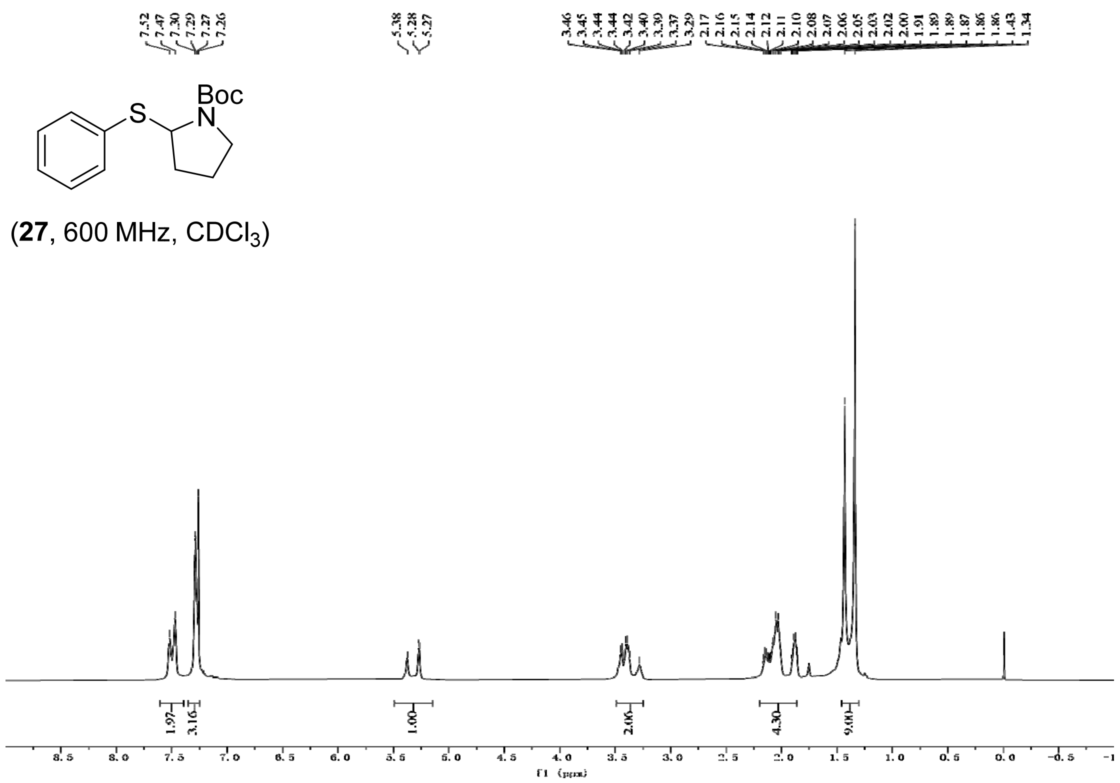
7.52
7.47
7.30
7.29
7.27
7.26
5.38
5.28
5.27
3.46
3.45
3.44
3.44
3.42
3.40
3.39
3.37
3.29
2.17
2.16
2.15
2.14
2.12
2.11
2.10
2.08
2.07
2.06
2.05
2.03
2.02
2.00
1.91
1.89
1.89
1.87
1.86
1.43
1.34
Boc
N
S
(27, 600 MHz, CDCl3)
1.97
3.16
1.00
2.06
4.30
9.00
f1 (ppm)

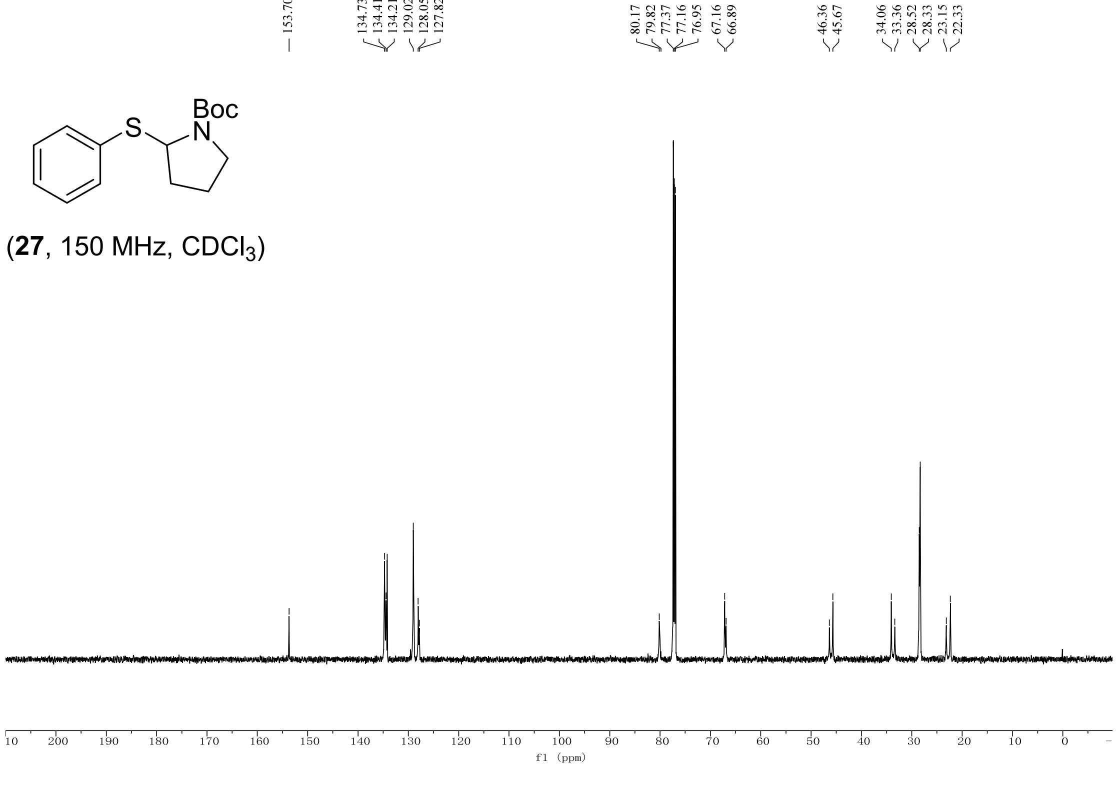
153.70
134.73
134.41
134.21
129.02
128.05
127.82
80.17
79.82
77.37
77.16
76.95
67.16
66.89
46.36
45.67
34.06
33.36
28.52
28.33
23.15
22.33
Boc
N
S
(27, 150 MHz, CDCl3)
f1 (ppm)

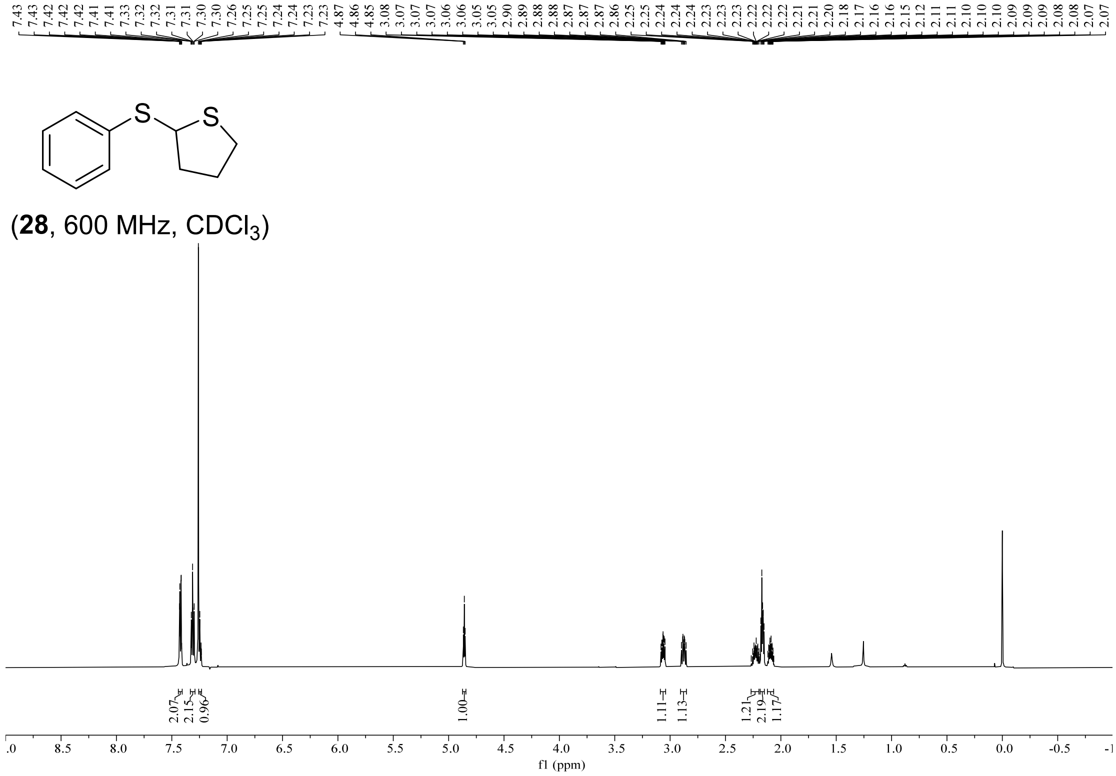

(**28**, 600 MHz, $CDCl_3$)

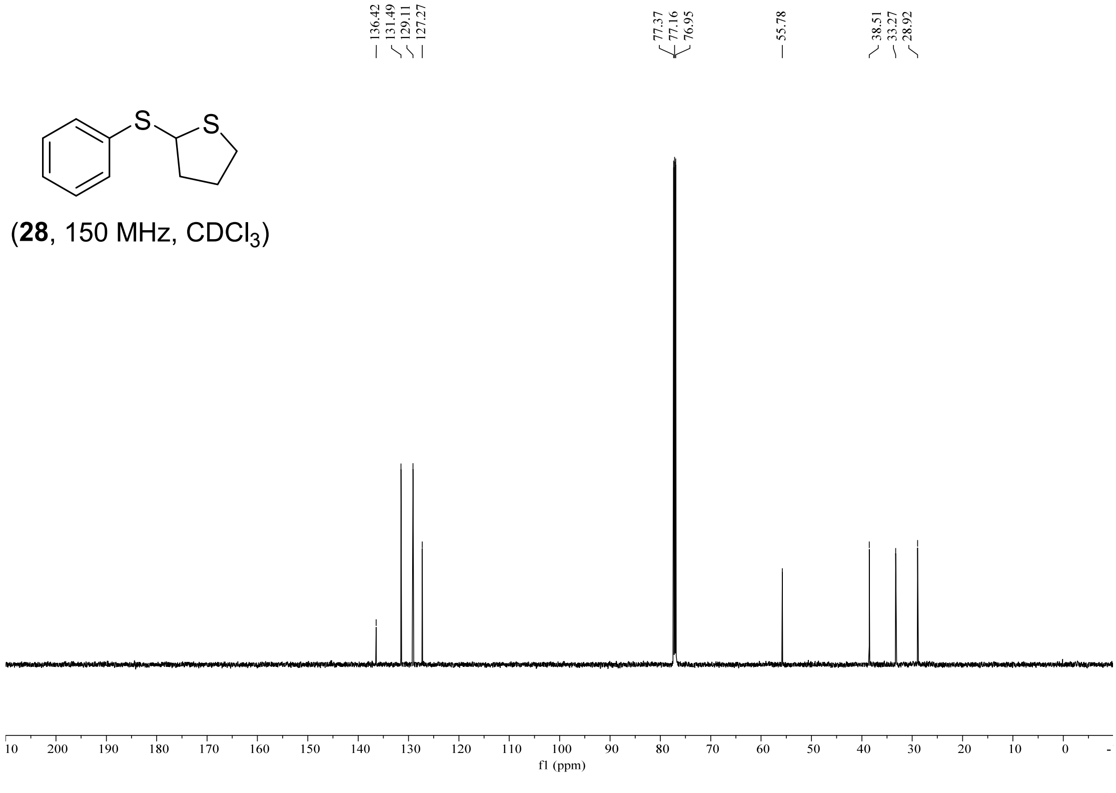

(**28**, 150 MHz, $CDCl_3$)

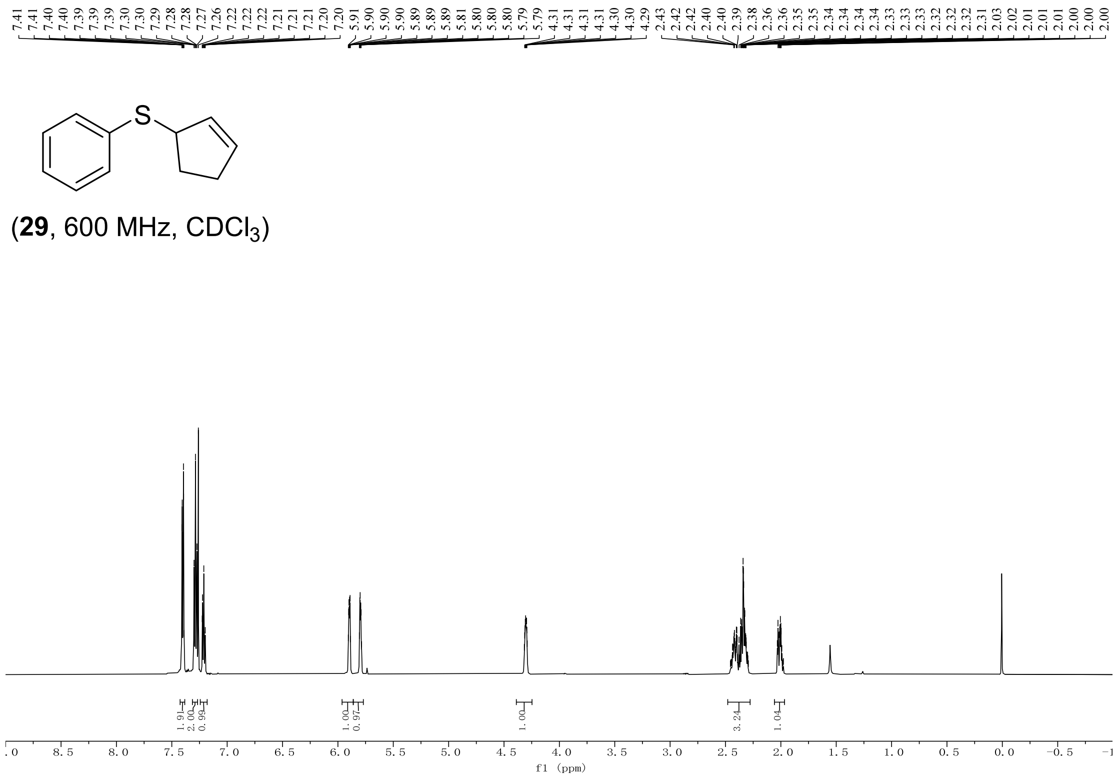

(**29**, 600 MHz, $CDCl_3$)

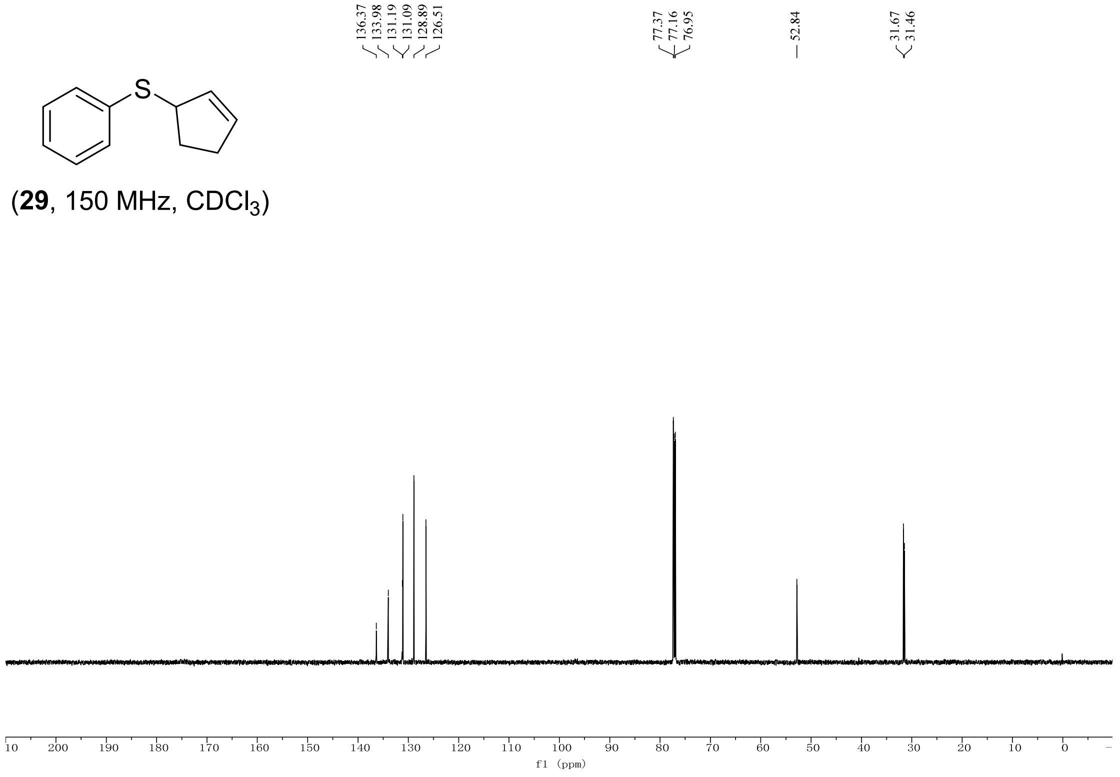

(**29**, 150 MHz, $CDCl_3$)

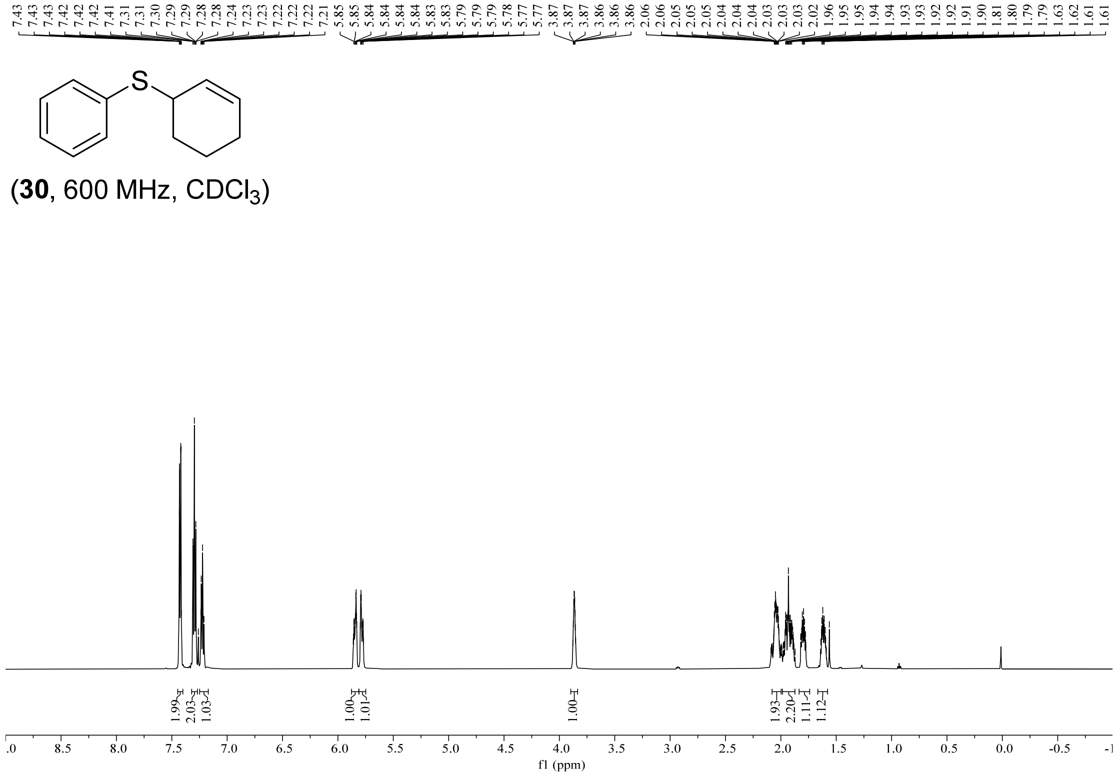

(**30**, 600 MHz, $CDCl_3$)

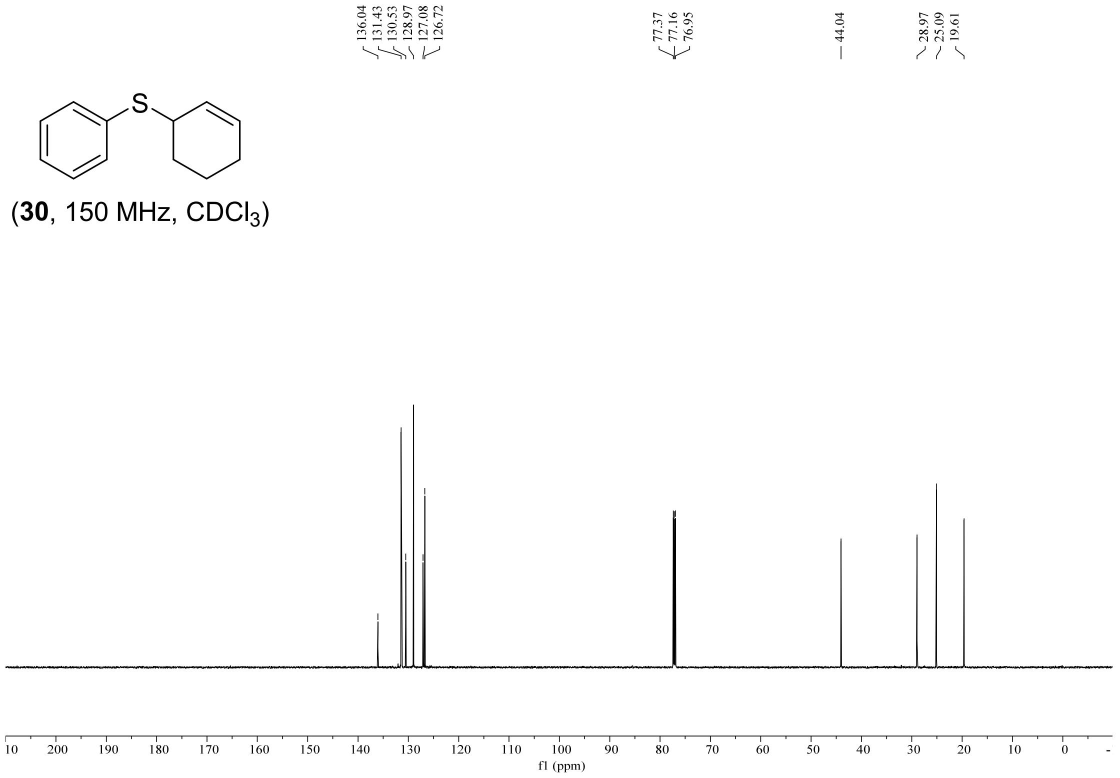

(**30**, 150 MHz, $CDCl_3$)

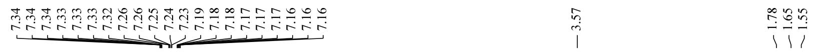

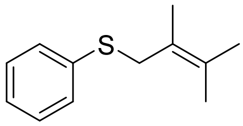

($\mathbf{31}$, 600 MHz, $CDCl_3$)

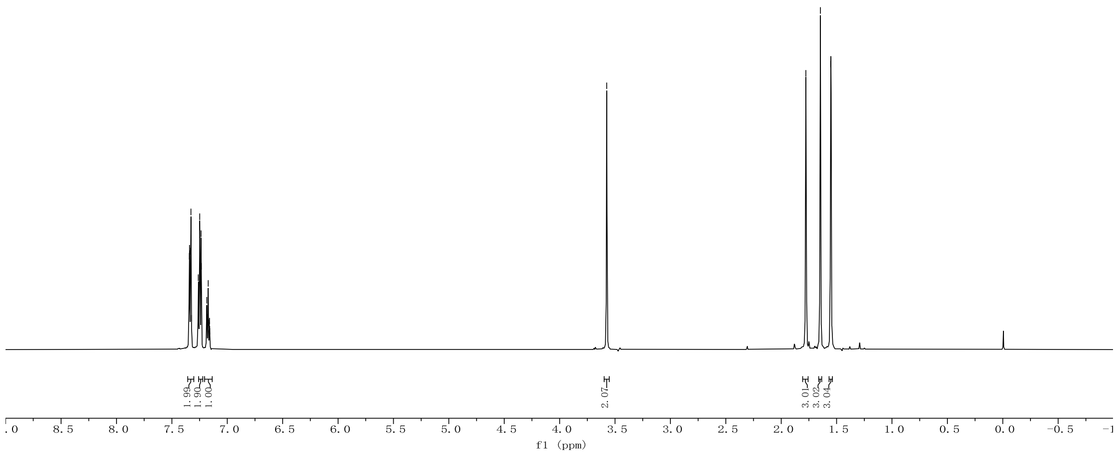

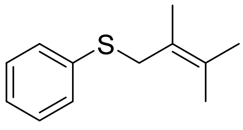

($\mathbf{31}$, 150 MHz, $CDCl_3$)

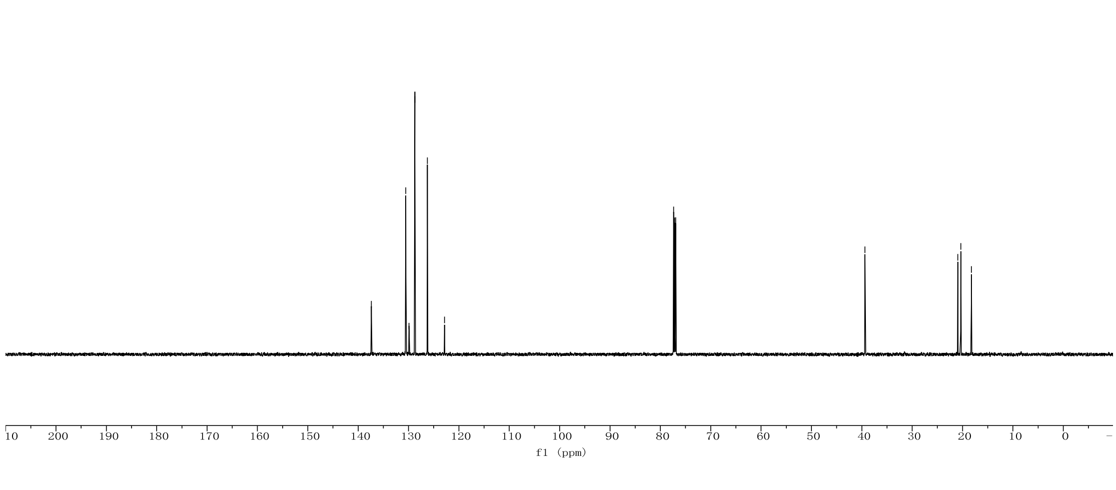

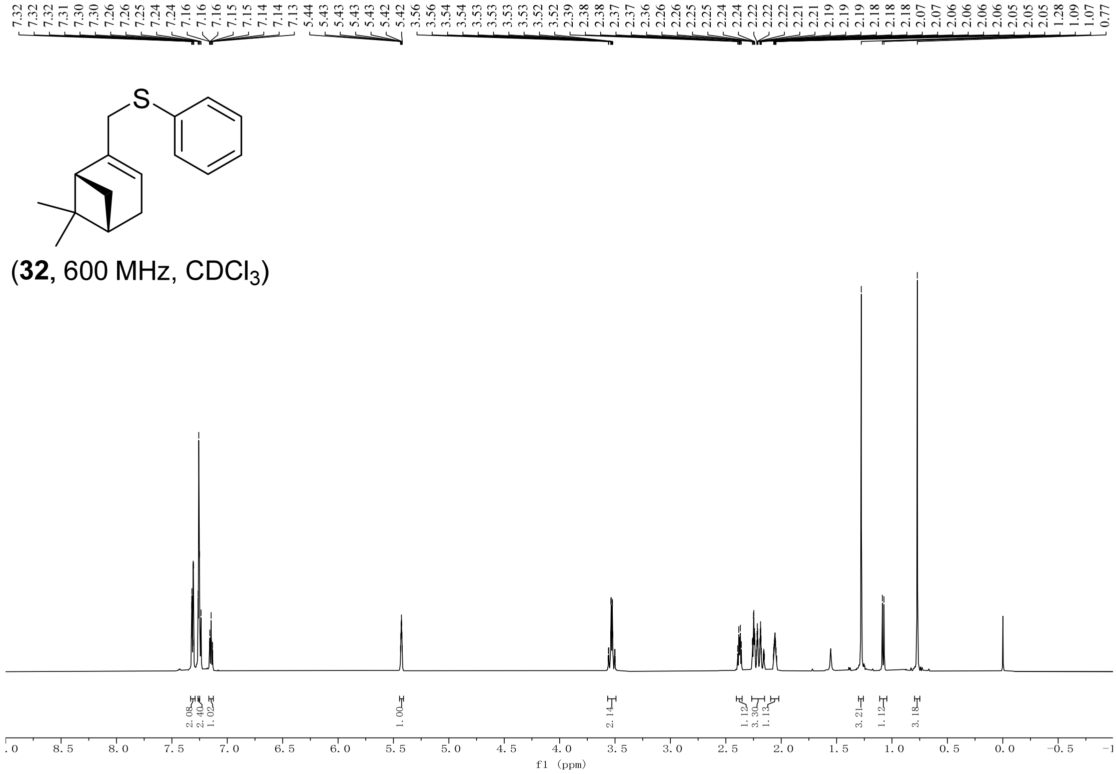
7.32 7.32 7.31 7.30 7.30 7.26 7.26 7.25 7.24 7.24 7.16 7.16 7.16 7.15 7.15 7.14 7.14 7.13 5.44 5.43 5.43 5.43 5.43 5.42 3.56 3.56 3.54 3.54 3.53 3.53 3.53 3.53 3.52 2.39 2.38 2.38 2.37 2.37 2.36 2.26 2.26 2.25 2.25 2.24 2.24 2.22 2.22 2.22 2.21 2.19 2.19 2.19 2.18 2.18 2.18 2.07 2.07 2.06 2.06 2.06 2.05 2.05 2.05 1.28 1.09 1.07 0.77
S
(32, 600 MHz, CDCl3)
2.08 2.40 1.02 1.00 2.14 1.12 3.30 1.13 3.21 1.12 3.18
f1 (ppm)

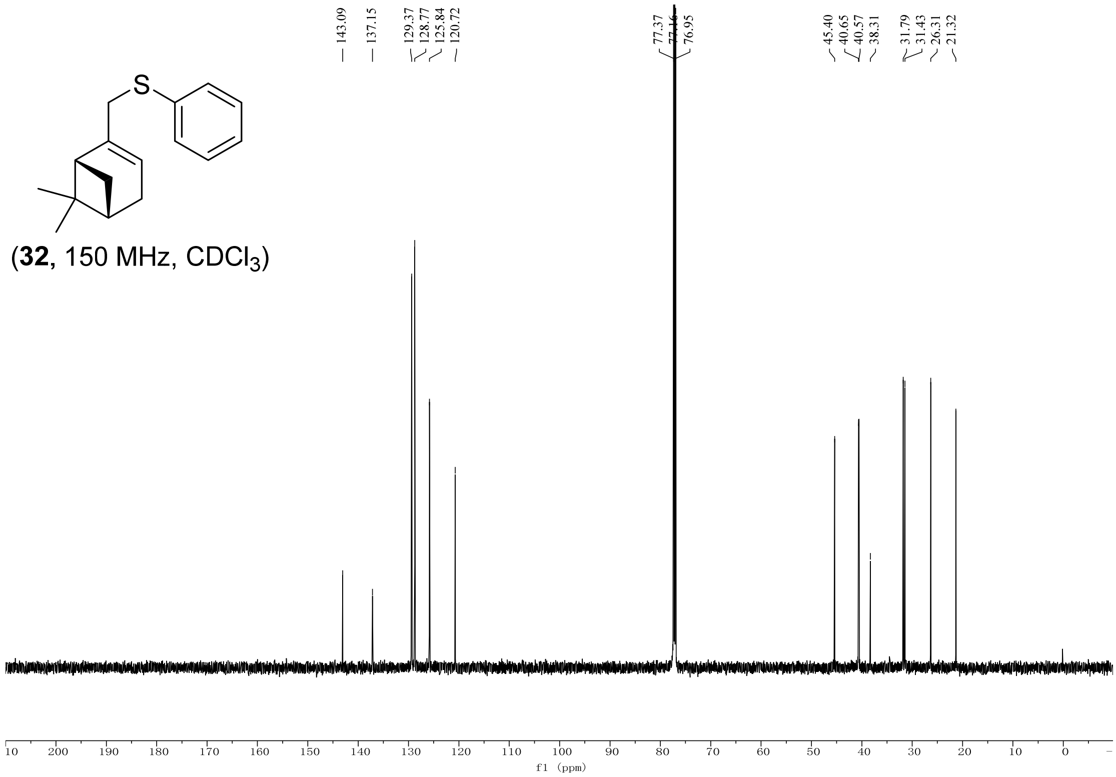
143.09 137.15 129.37 128.77 125.84 120.72 77.37 77.16 76.95 45.40 40.65 40.57 38.31 31.79 31.43 26.31 21.32
S
(32, 150 MHz, CDCl3)
f1 (ppm)

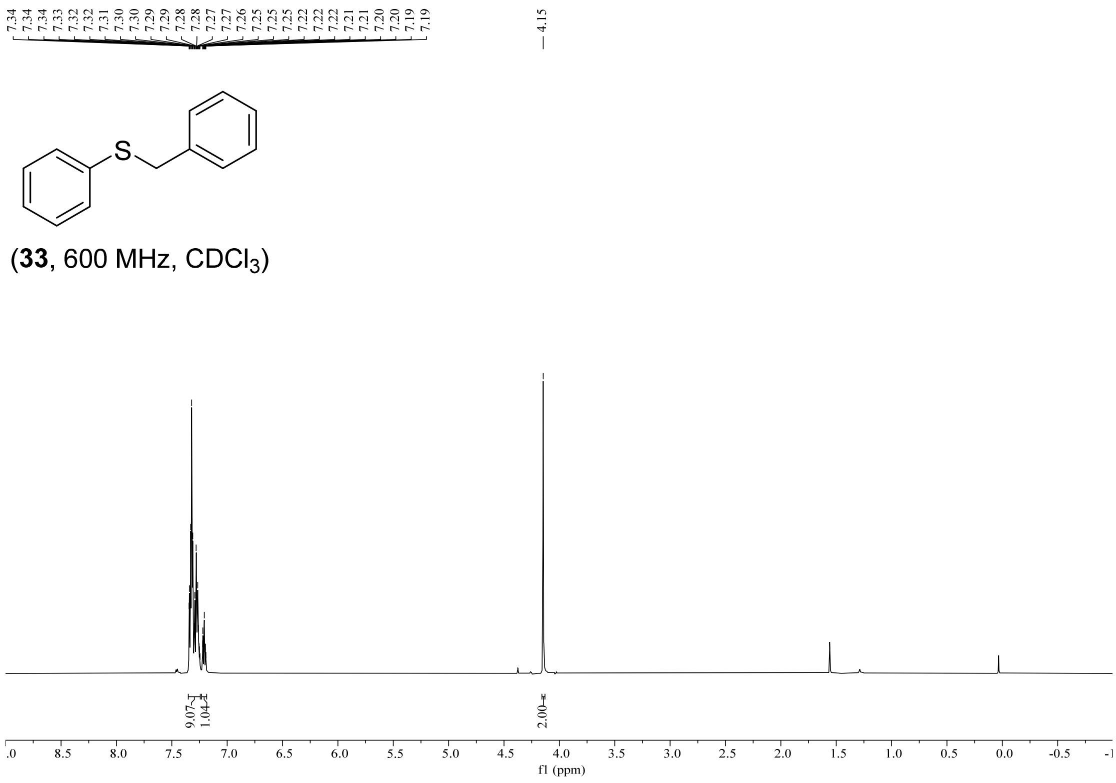

(**33**, 600 MHz, $CDCl_3$)

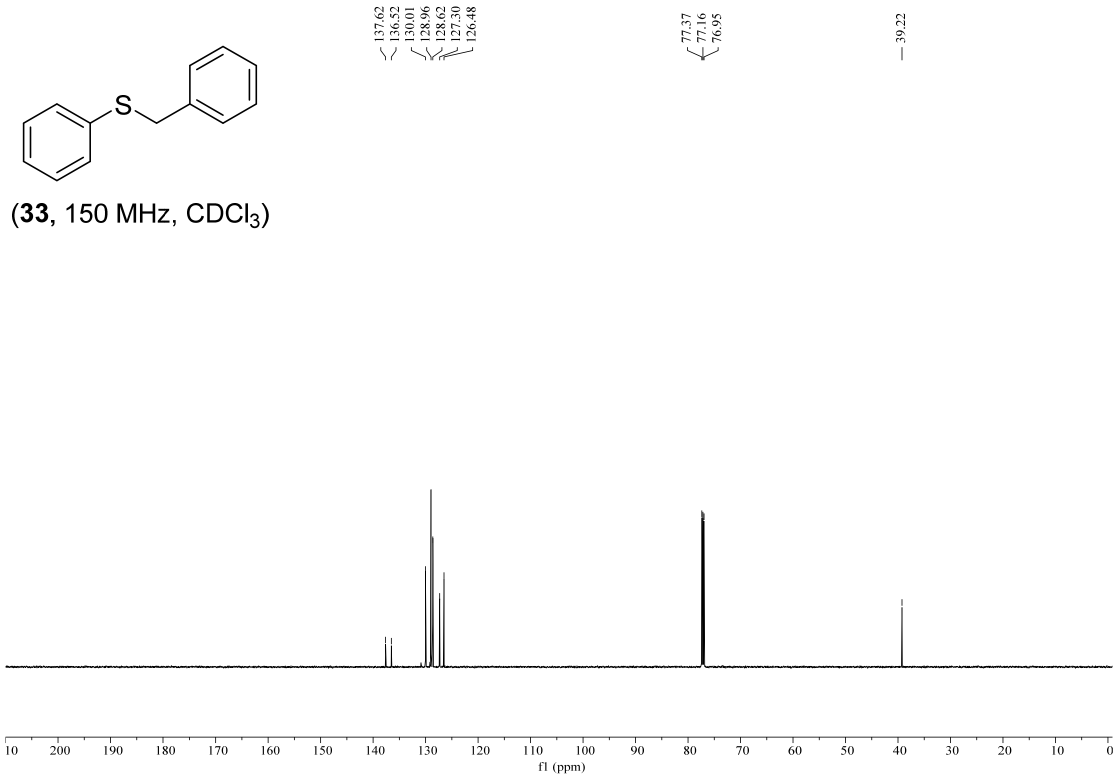

(**33**, 150 MHz, $CDCl_3$)

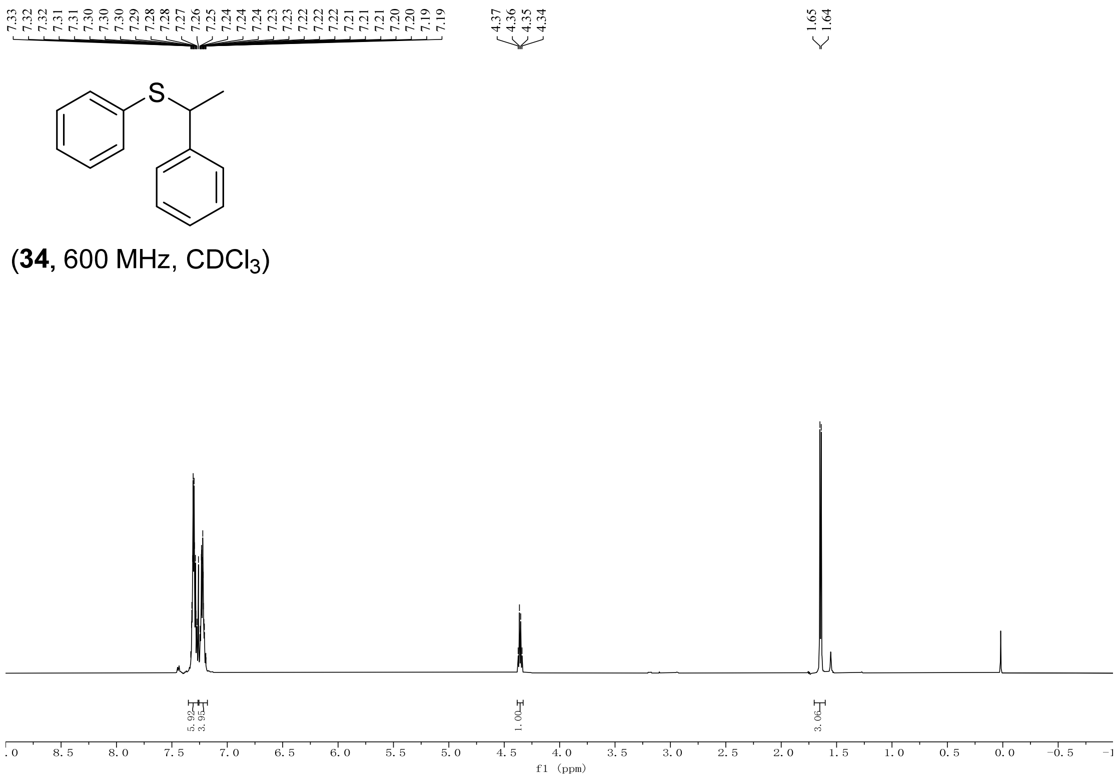

(**34**, 600 MHz, $CDCl_3$)

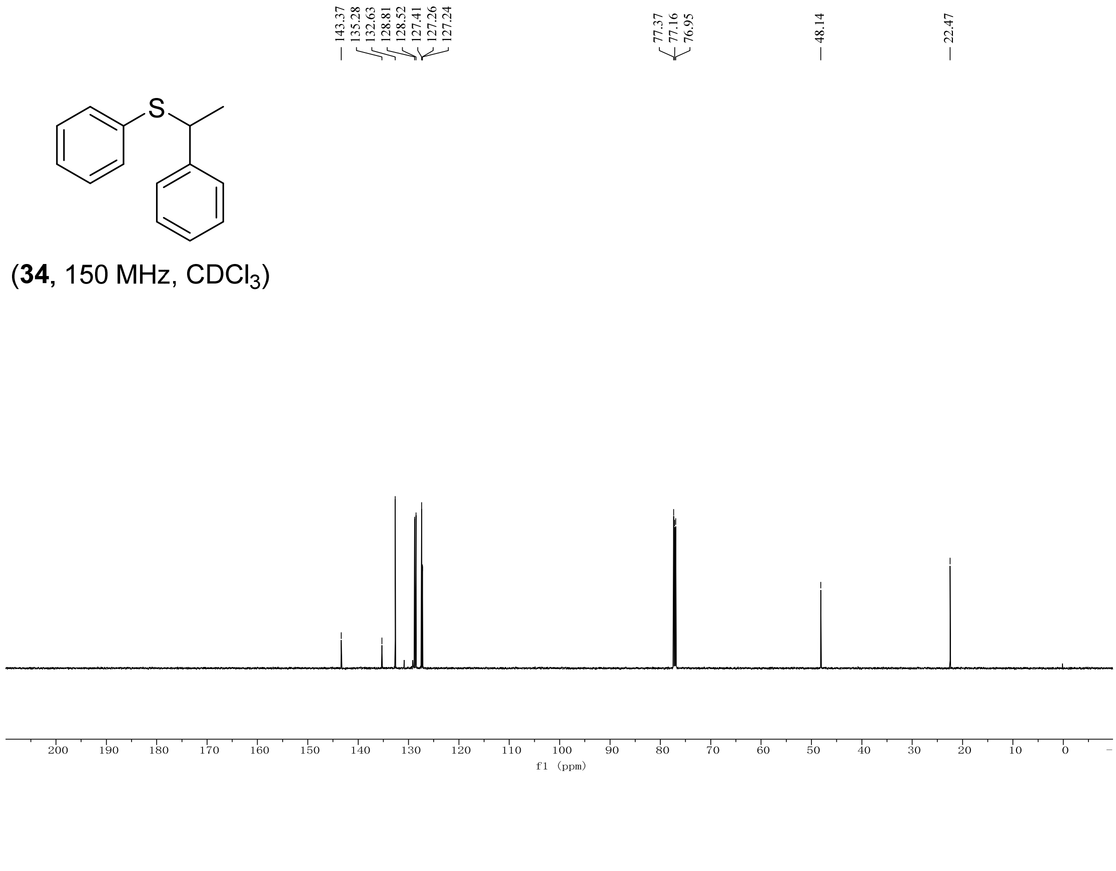

(**34**, 150 MHz, $CDCl_3$)

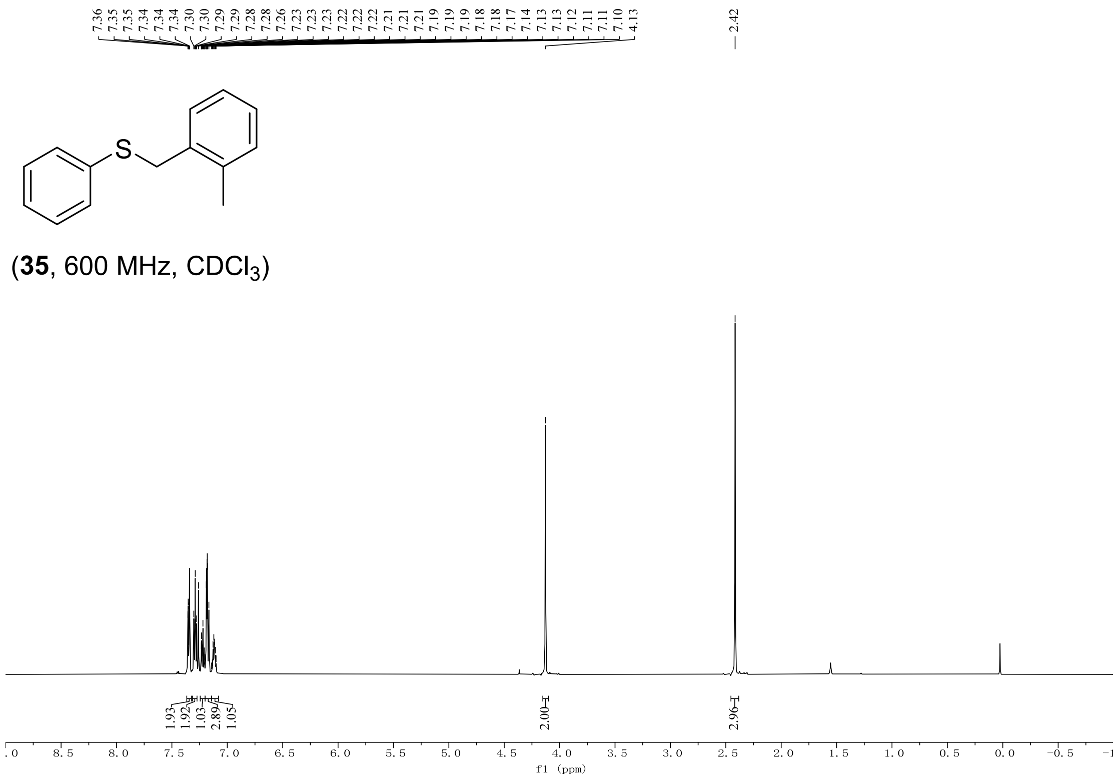
7.36 7.35 7.35 7.34 7.34 7.34 7.30 7.30 7.29 7.29 7.28 7.28 7.26 7.23 7.23 7.23 7.22 7.22 7.22 7.21 7.21 7.21 7.19 7.19 7.19 7.18 7.18 7.17 7.14 7.13 7.13 7.12 7.11 7.11 7.10 4.13
2.42
S
(**35**, 600 MHz, $CDCl_3$)
1.93 1.92 1.03 2.89 1.05
2.00
2.96
f1 (ppm)

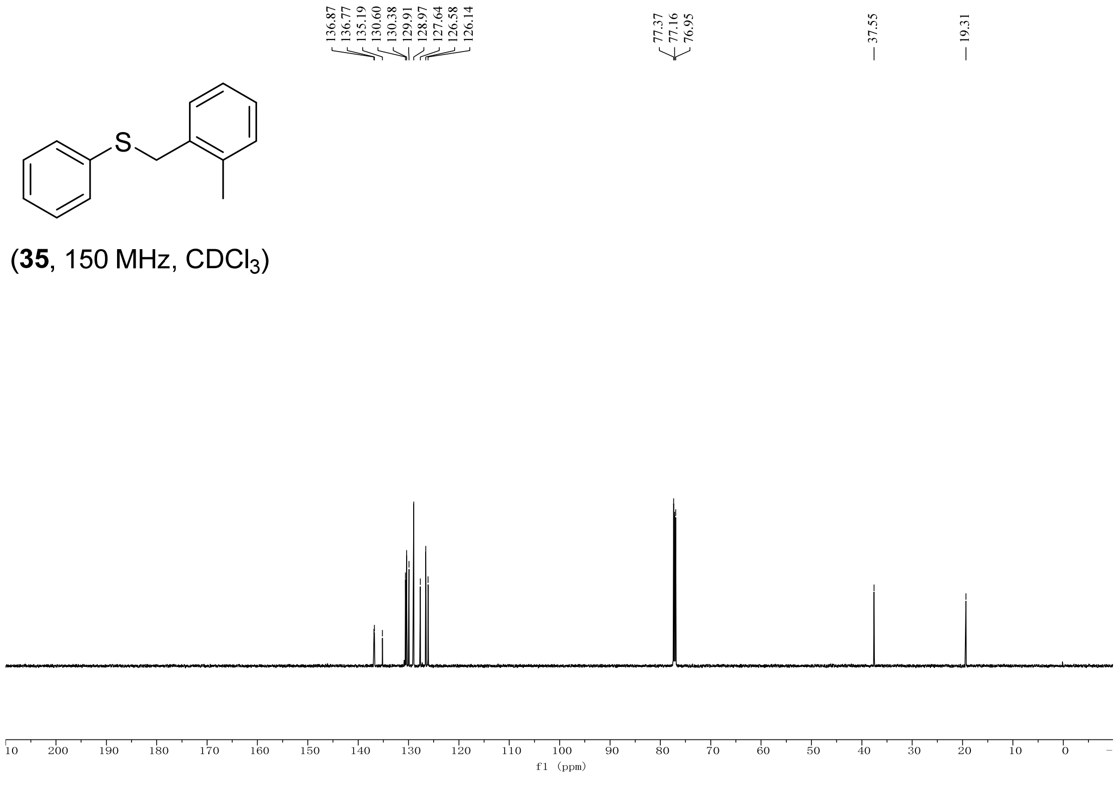
136.87 136.77 135.19 130.60 130.38 129.91 128.97 127.64 126.58 126.14
77.37 77.16 76.95
37.55
19.31
S
(**35**, 150 MHz, $CDCl_3$)
f1 (ppm)

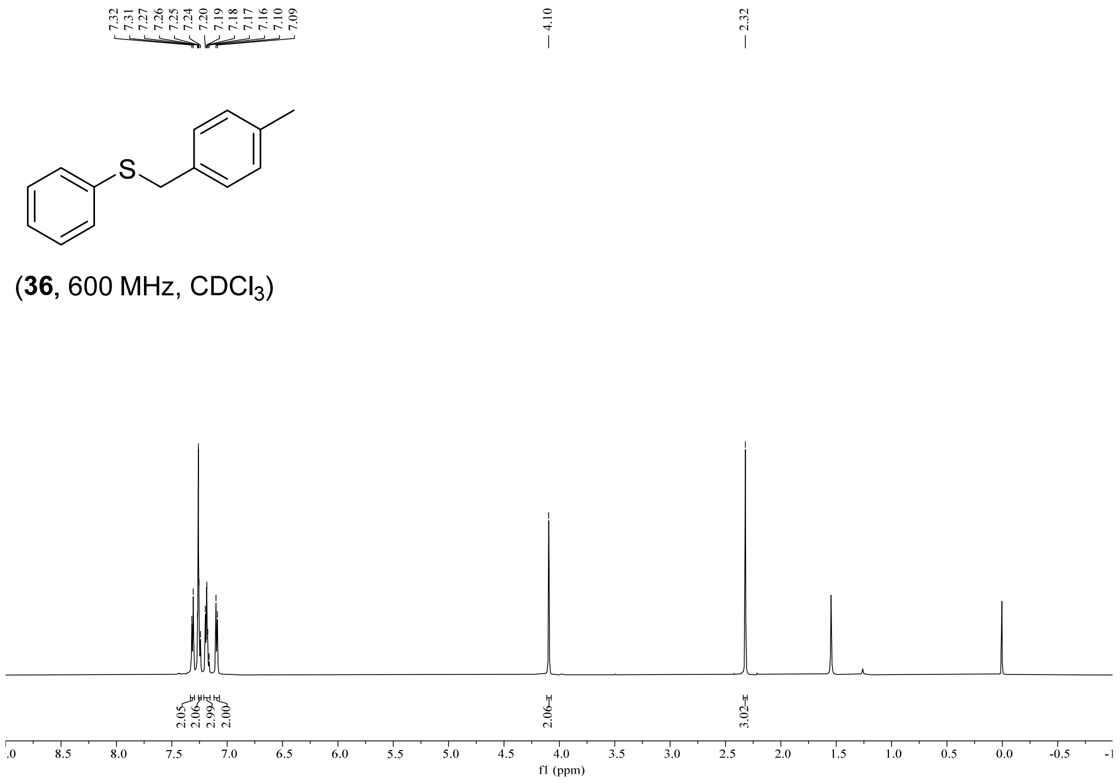
7.32
7.31
7.27
7.26
7.25
7.24
7.20
7.19
7.18
7.17
7.16
7.10
7.09
4.10
2.32
S
(36, 600 MHz, CDCl3)
2.05
2.06
2.99
2.00
2.06
3.02
f1 (ppm)

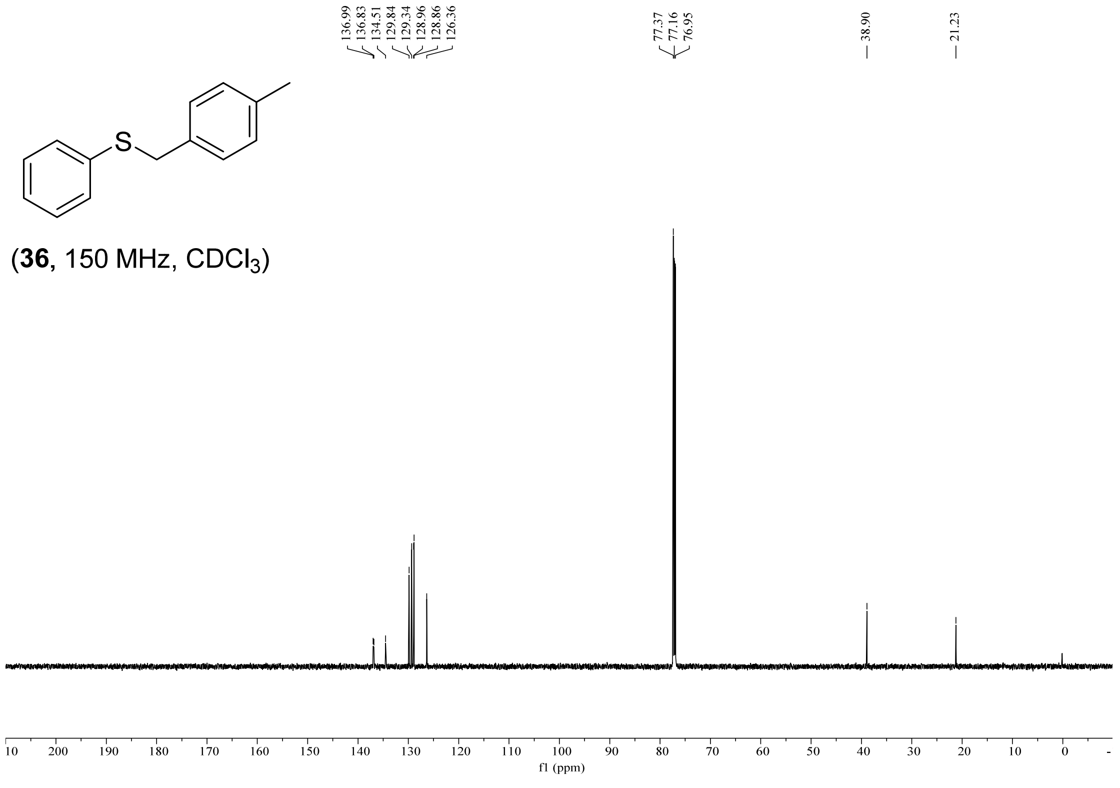
136.99
136.83
134.51
129.84
129.34
128.96
128.86
126.36
77.37
77.16
76.95
38.90
21.23
S
(36, 150 MHz, CDCl3)
f1 (ppm)

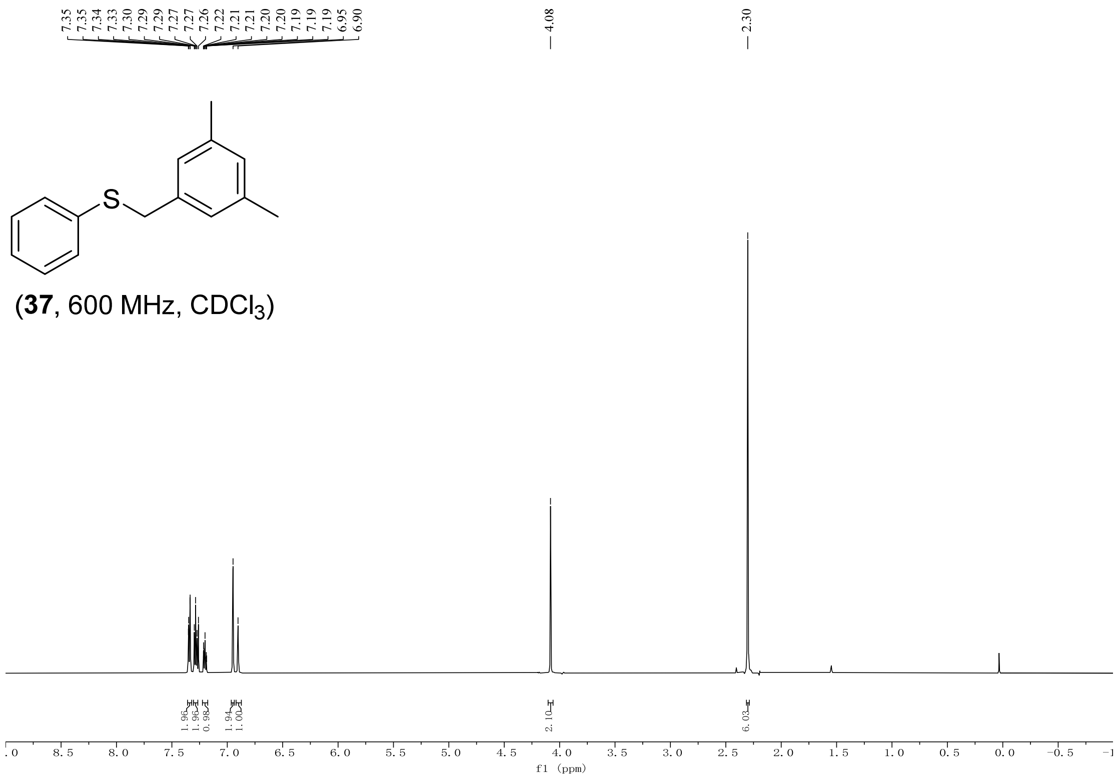

(**37**, 600 MHz, $CDCl_3$)

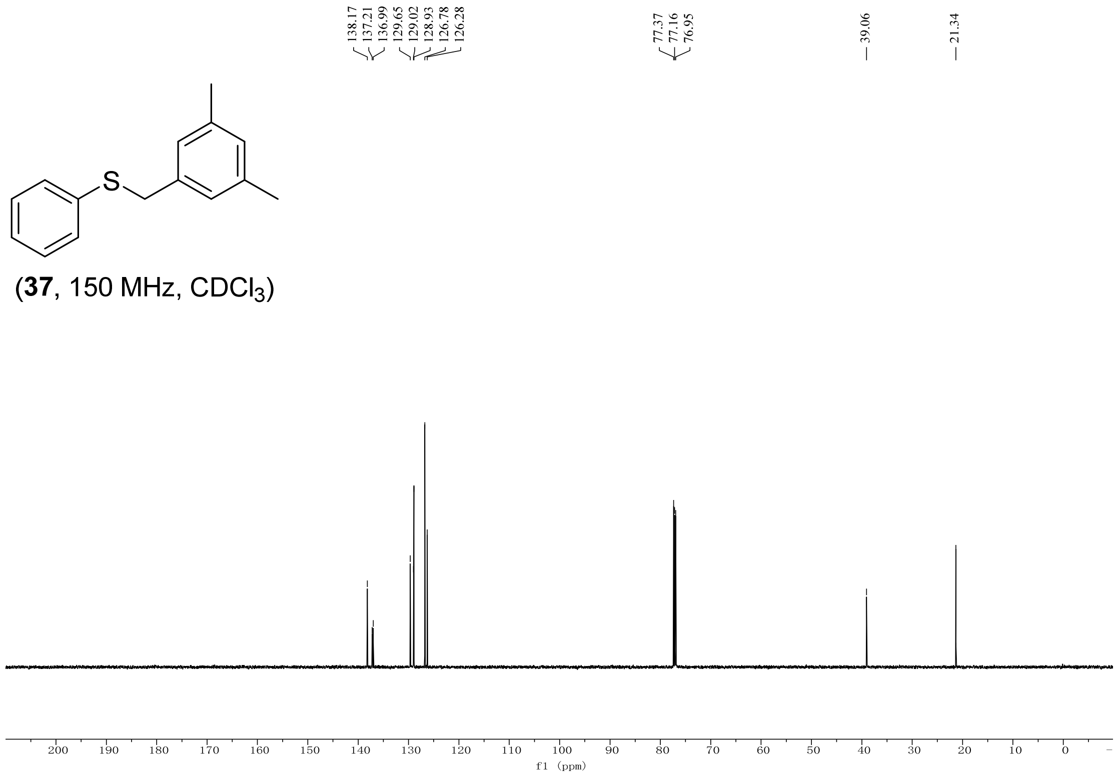

(**37**, 150 MHz, $CDCl_3$)

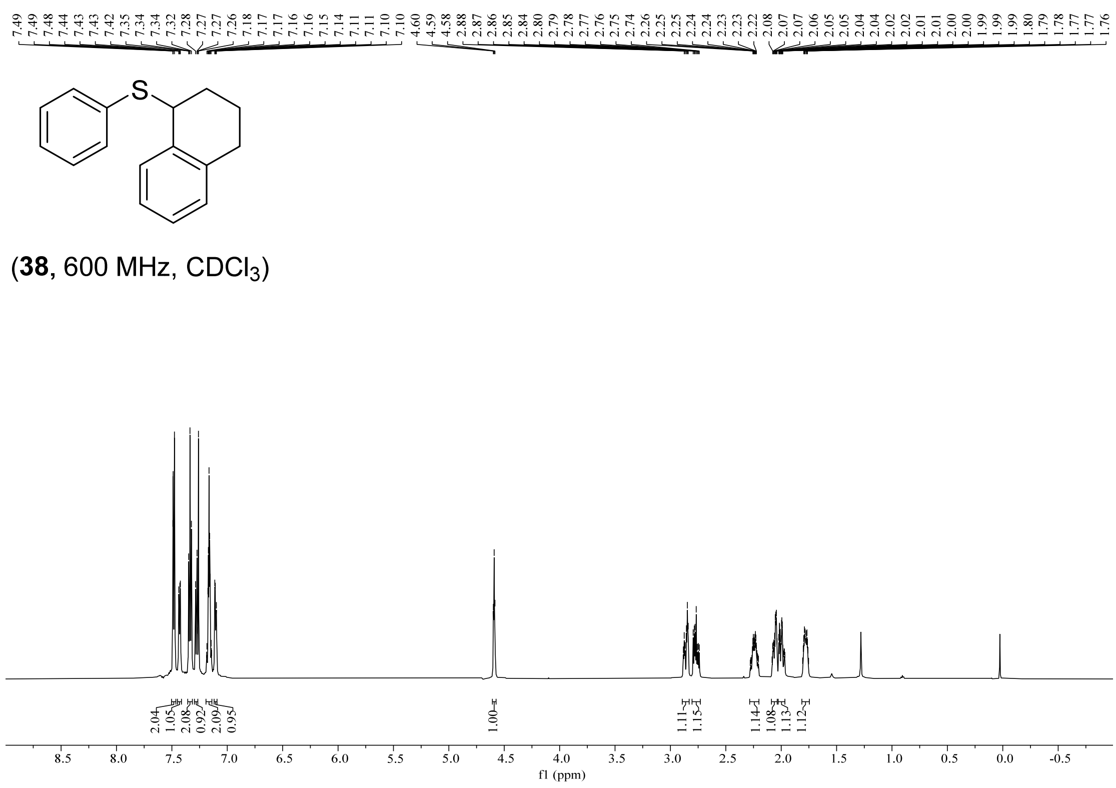

(**38**, 600 MHz, $CDCl_3$)

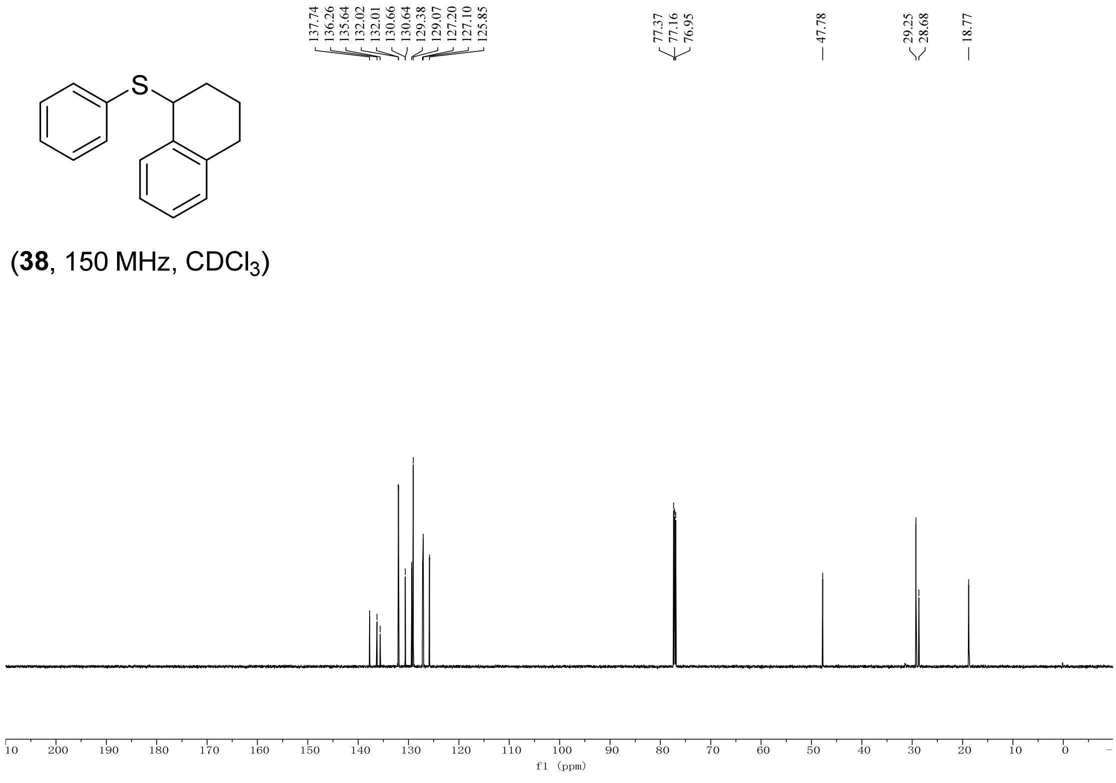

(**38**, 150 MHz, $CDCl_3$)

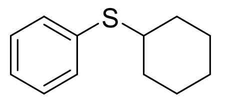

(**39**, 600 MHz, $CDCl_3$)

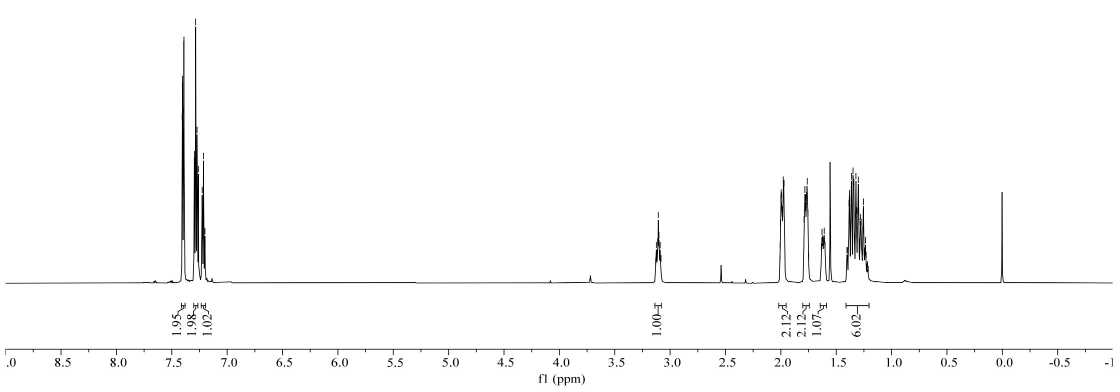

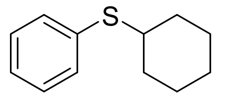

(**39**, 150 MHz, $CDCl_3$)

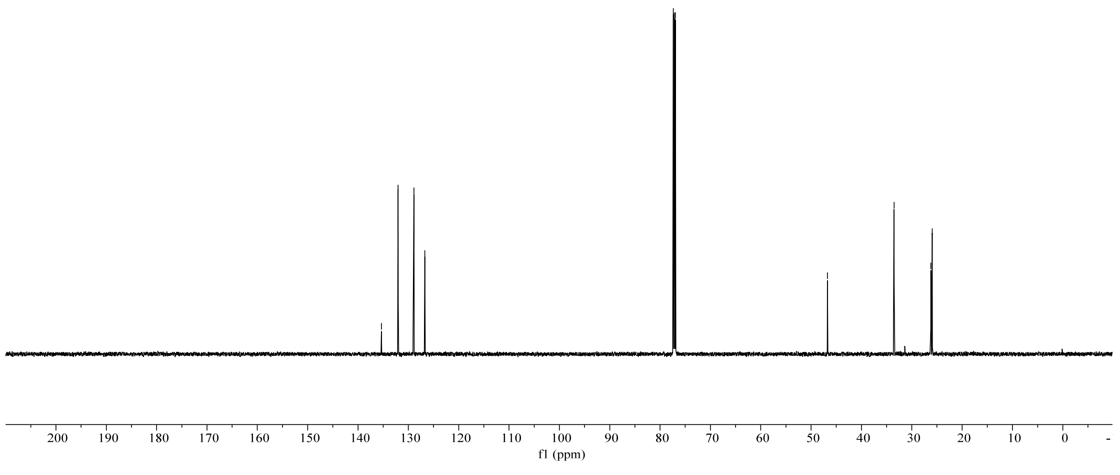

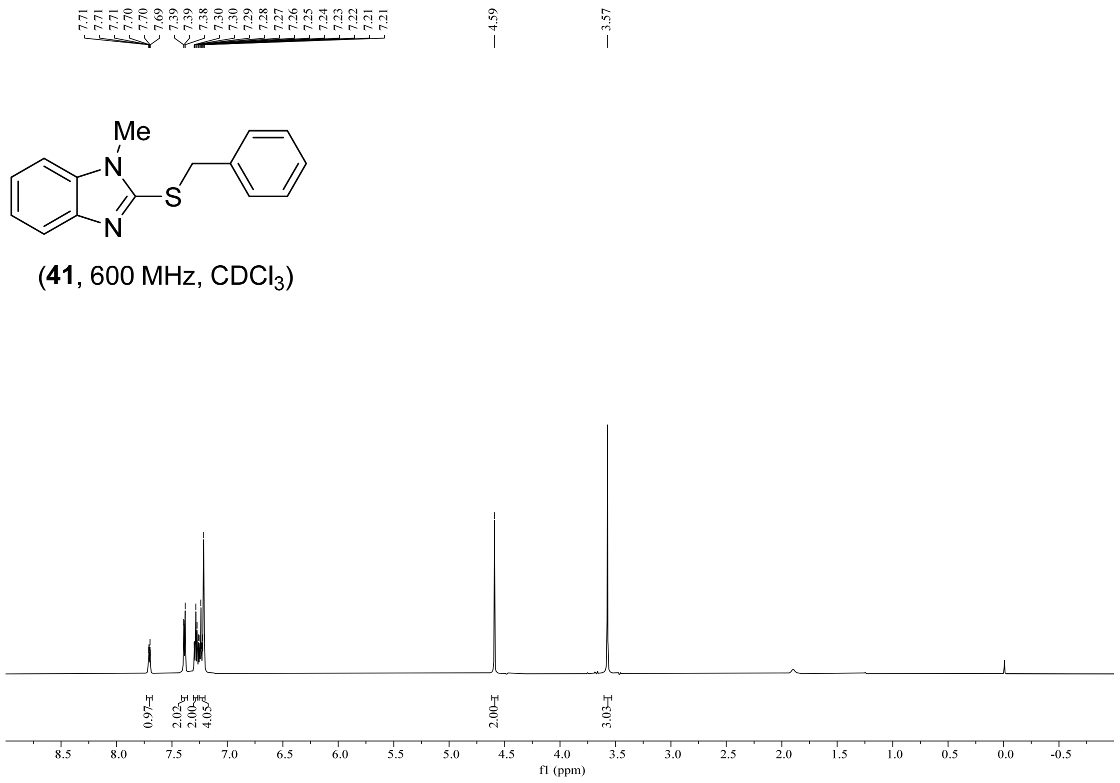

(**41**, 600 MHz, $CDCl_3$)

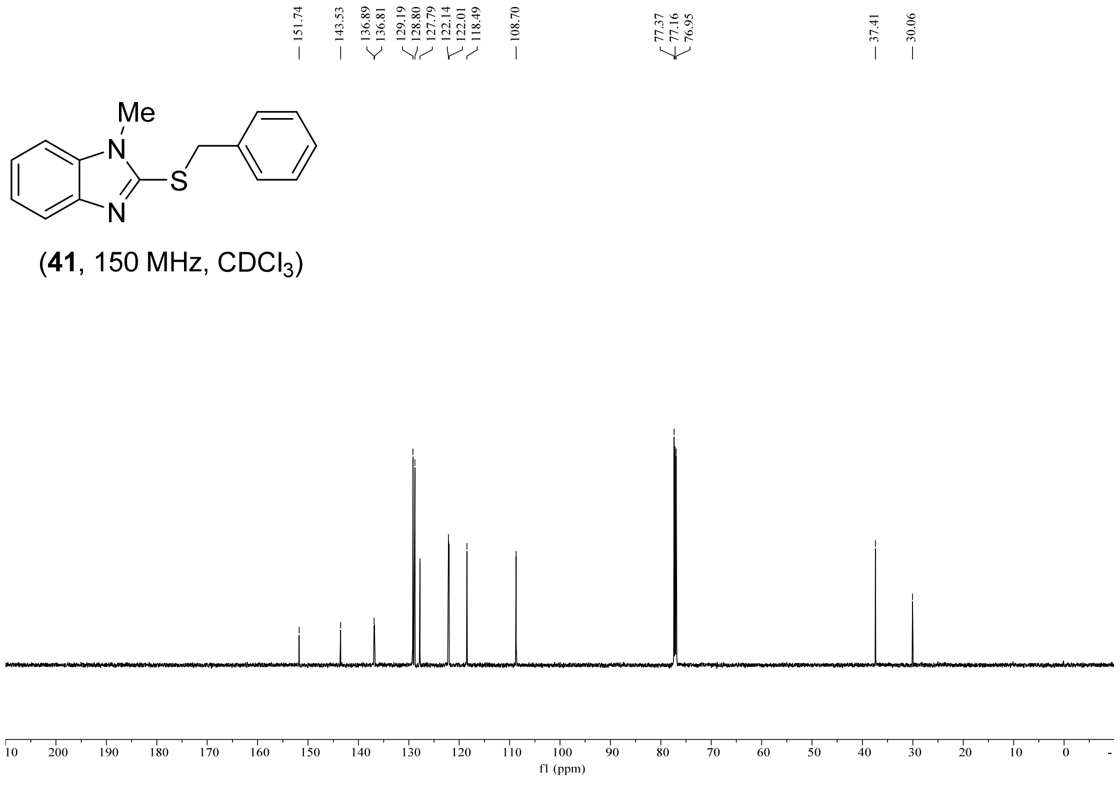

(**41**, 150 MHz, $CDCl_3$)

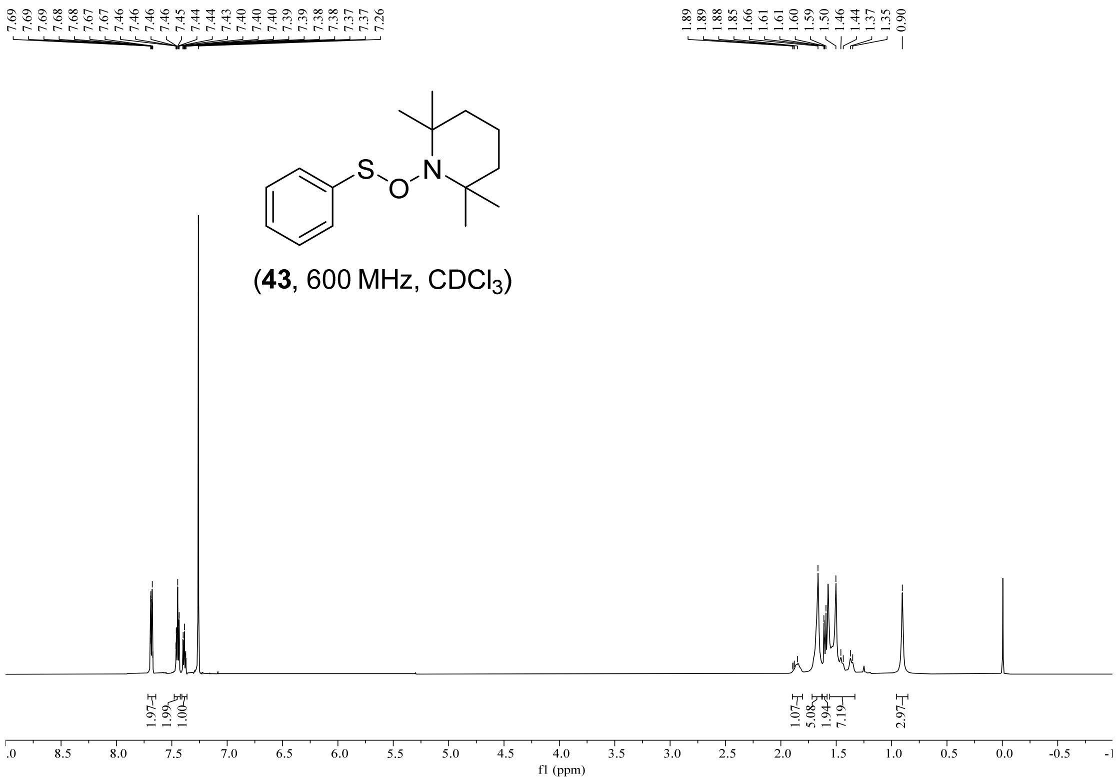
7.69 7.69 7.69 7.68 7.68 7.67 7.67 7.46 7.46 7.46 7.45 7.44 7.44 7.43 7.40 7.40 7.40 7.39 7.39 7.38 7.38 7.37 7.37 7.26
1.89 1.89 1.88 1.85 1.66 1.61 1.61 1.60 1.59 1.50 1.46 1.44 1.37 1.35 0.90
S
O
N
(43, 600 MHz, CDCl3)
1.97 1.99 1.00
1.07 5.08 1.94 7.19 2.97
f1 (ppm)

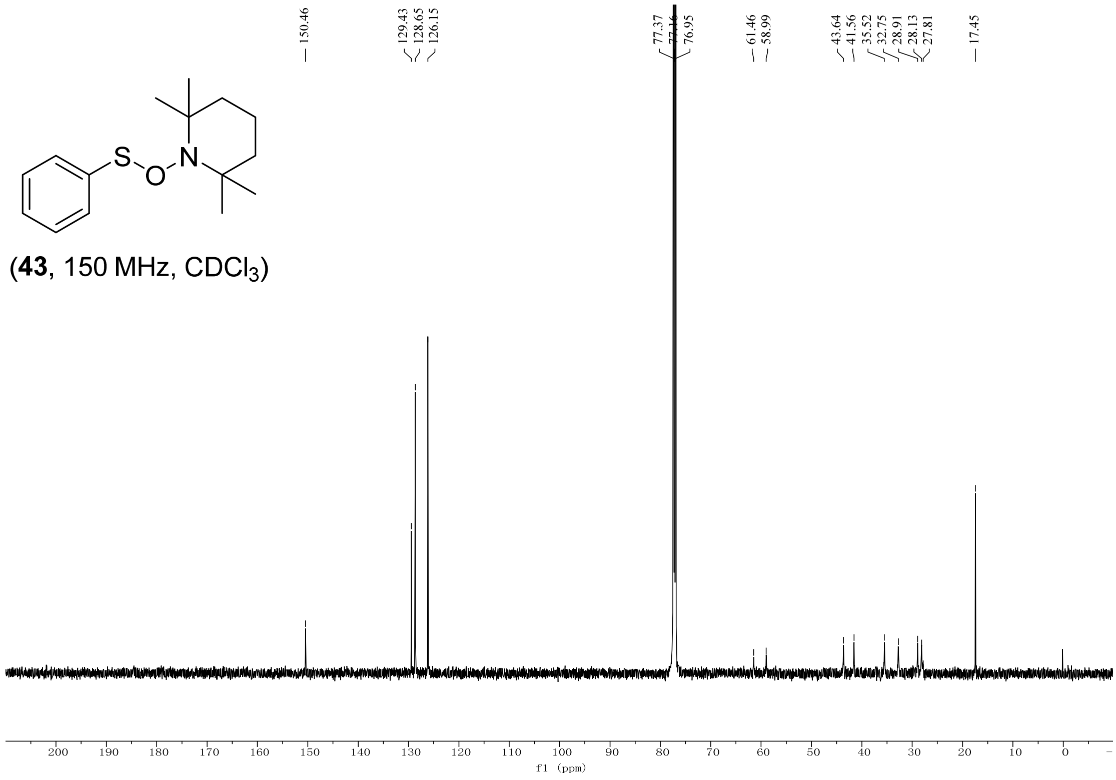
150.46
129.43 128.65 126.15
77.37 77.16 76.95
61.46 58.99
43.64 41.56 35.52 32.75 28.91 28.13 27.81
17.45
S
O
N
(43, 150 MHz, CDCl3)
f1 (ppm)

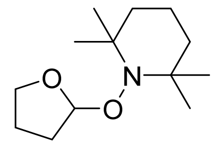

(**44**, 600 MHz, $CDCl_3$)

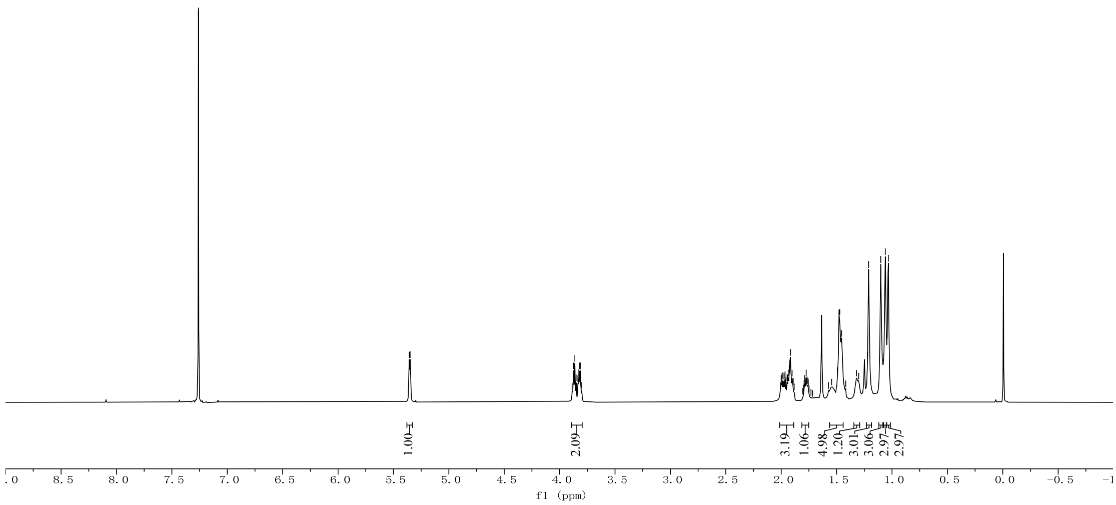

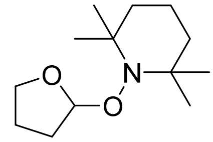

(**44**, 150 MHz, $CDCl_3$)

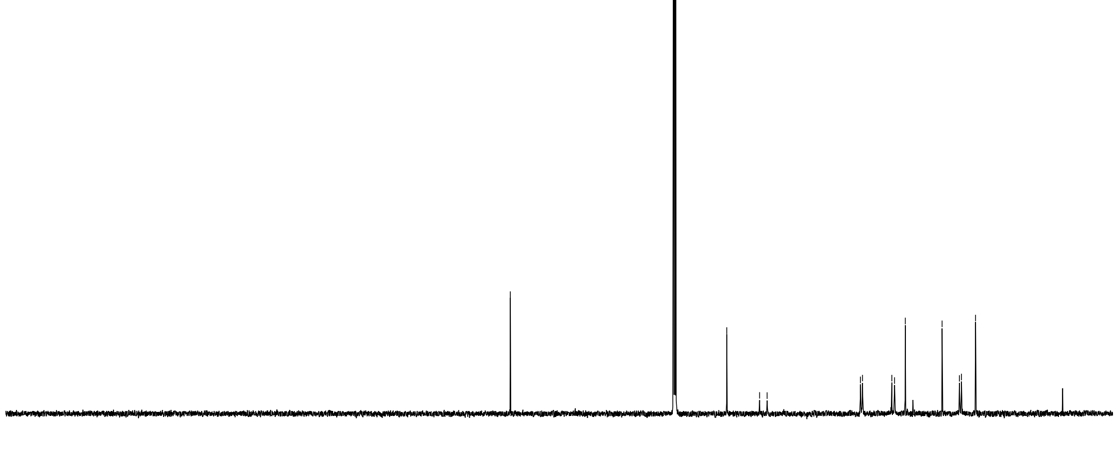

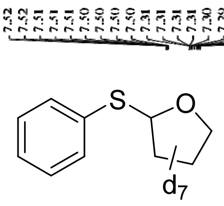

(**45**, 600 MHz, $CDCl_3$)

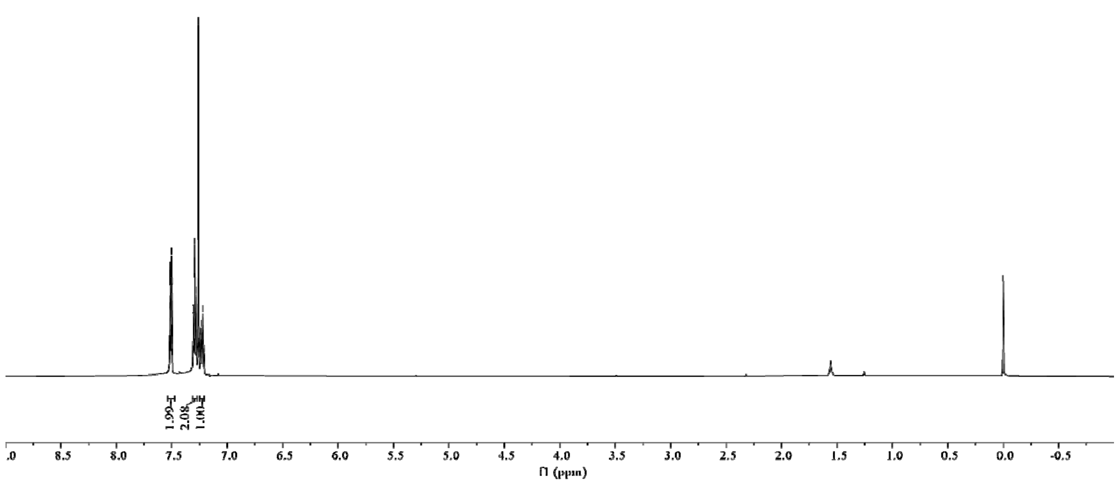

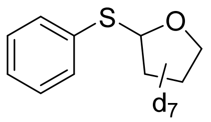

(**45**, 150 MHz, $CDCl_3$)

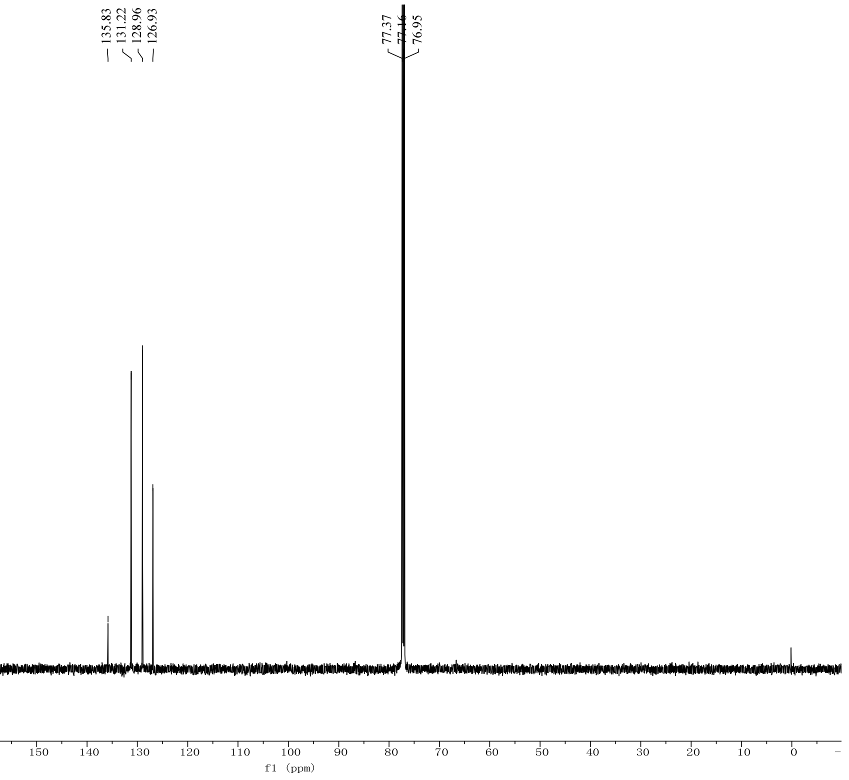

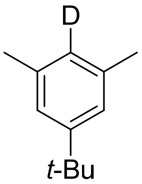

(**46**, 600 MHz, $CDCl_3$)

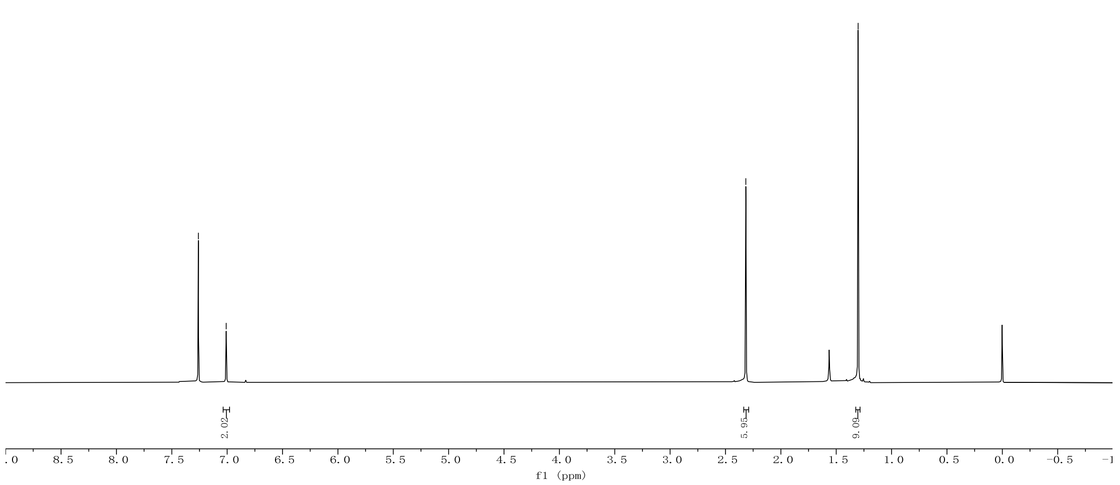

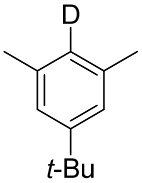

(**46**, 150 MHz, $CDCl_3$)

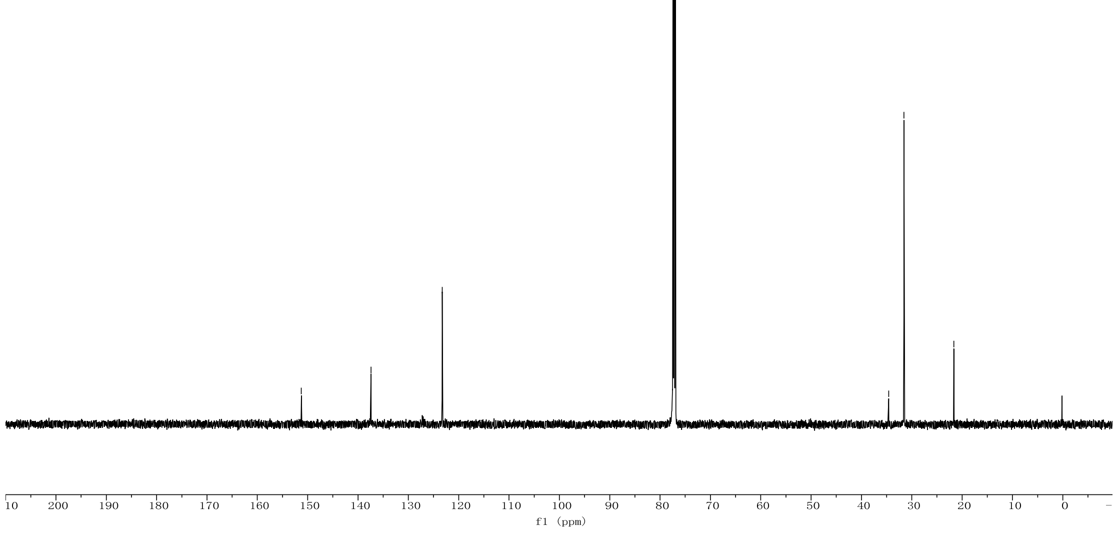

Supplement: SC-012-D1SC03667J-s001 [file SC-012-D1SC03667J-s001.pdf]
